# Thimerosal Inhibits Tumor Malignant Progression through Direct Action and Enhancing the Efficacy of PD-1-Based Immunotherapy

**DOI:** 10.32604/or.2025.071902

**Published:** 2026-01-19

**Authors:** Ping Wang, Yan-Han Chen, Ze-Tao Zhan, Jun-Xiang Zeng, Yu Chen, Yuan Lin, Tao Chen, Wei-Jie Zhou

**Affiliations:** 1 Department of General Surgery, Guangdong Provincial Key Laboratory of Precision Medicine for Gastrointestinal Tumor, Nanfang Hospital, Southern Medical University, Guangzhou, 510515, China; 2 State Key Laboratory of Organ Failure Research, Cancer Research Institute, School of Basic Medical Sciences, Southern Medical University, Guangzhou, 510515, China; 3 Department of Gastroenterology, The Seventh Affiliated Hospital of Southern Medical University, Foshan, 528244, China; 4 The Second School of Clinical Medicine, Southern Medical University, Guangzhou, 510280, China; 5 Department of Gastrointestinal and Hernia Surgery, Ganzhou Hospital-Nanfang Hospital, Southern Medical University, Ganzhou, 341000, China

**Keywords:** Thimerosal, colorectal cancer, melanoma, Janus kinase 1/signal transducer and activator of transcription 3, programmed cell death protein 1, immunotherapy, mercury ions, repurposed drug

## Abstract

**Background:**

Thimerosal is a mercury-containing preservative widely used in vaccines. This study aimed to investigate its potential antitumor effects and mechanisms in solid malignancies, particularly colorectal cancer (CRC) and melanoma.

**Methods:**

A combination of *in vitro* and *in vivo* approaches was employed. Cell proliferation, apoptosis, migration, and invasion were assessed using Cell Counting Kit-8 (CCK-8), colony formation, ATP viability, Western blotting, flow cytometry, wound-healing and Transwell assays. Subcutaneous, lung metastases, and Azoxymethane/Dextran Sulfate Sodium Salt (AOM/DSS)-induced colitis-associated CRC models were established to examine antitumor efficacy and safety. The functional role of mercury ions was validated using structural analogues. Mechanistic studies included RNA sequencing, Western blot, and immunohistochemical analysis of CD8^+^ T cell infiltration. The synergistic effect with programmed cell death protein 1 (PD-1) antibody therapy was also evaluated.

**Results:**

Thimerosal potently inhibited tumor growth (with IC_50_ values ranging from 0.1 to 1 μM *in vitro*) and significantly prolonged survival without overt toxicity *in vivo*. Mechanistically, mercury ions were identified as critical functional sites mediating Thimerosal’s antitumor effects. Specifically, Thimerosal inhibited the phosphorylation of Janus kinase 1(JAK1) and signal transducer and activator of transcription 3 (STAT3). Furthermore, it enhanced the infiltration of CD8^+^ T cells into the tumor microenvironment and synergistically augmented the efficacy of anti-PD-1 therapy.

**Conclusion:**

Thimerosal exerts dual antitumor roles by direct JAK1/STAT3 inhibition and immune modulation via CD8^+^ T cell recruitment. It represents a promising repurposed drug and immunotherapeutic adjuvant for CRC and melanoma.

## 1 Introduction

Cancer remains a significant global health challenge, with nearly 20 million new cases and 9.7 million cancer-related deaths reported in 2022 [1]. Among these, Colorectal cancer (CRC) ranks as the third most commonly diagnosed malignancy and the second leading cause of cancer-related mortality worldwide [2], posing a major clinical burden and an urgent need for improved therapeutic strategies. Despite advancements in therapeutic approaches, including immunotherapy [3–5], a substantial number of cancer patients fail to benefit [6], emphasizing the need for continued exploration of innovative treatment strategies.

In recent years, efforts to repurpose existing drugs for cancer treatment have yielded promising results. For instance, the antidiabetic drug metformin has been shown to inhibit tumor cell growth [7,8], improve the tumor microenvironment [9,10], and enhance the efficacy of radiotherapy and chemotherapy. Combining metformin with immunotherapies such as anti-programmed cell death protein 1 (PD-1) therapy has demonstrated potential to further augment therapeutic outcomes, particularly in breast cancer and other malignancies [11–13]. Similarly, aspirin, traditionally known for its anti-inflammatory, antipyretic, and analgesic properties, has been found to significantly reduce the incidence and mortality of CRC and other cancers with long-term, low-dose use [14,15]. Ongoing clinical trials are investigating its adjuvant role in cancer treatment. The immunosuppressant sirolimus (also known as rapamycin), originally used to prevent organ transplant rejection, has been repurposed for cancer therapy. It suppresses tumor cell growth and proliferation by targeting the mechanistic target of rapamycin (mTOR) signaling pathway and has shown efficacy in treating kidney cancer, soft tissue sarcoma, and cancers with abnormal mTOR pathway activation [16–18]. These examples underscore the potential of re-evaluating the multifunctionality of existing drugs to discover cost-effective and clinically translatable cancer therapies.

Thimerosal, an organic compound containing alkyl mercury, has long been used as a preservative due to its potent antibacterial properties and ability to stabilize antigens in vaccines [19,20]. Preliminary studies have suggested that Thimerosal exhibits antitumor effects in cancers such as osteosarcoma [21], leukemia [22], oral cancer [23] and gastric cancer [24]. More recently, its efficacy has been demonstrated in glioblastoma through inhibition of the thioredoxin system [25]. These effects are mediated through mechanisms involving calcium influx, activation of the p38 mitogen-activated protein kinase (MAPK) pathway, and the generation of reactive oxygen species (ROS), and specific inhibition of thioredoxin reductase (TrxR) [26]. However, existing evidence remains predominantly derived from *in vitro* models, creating a significant knowledge gap regarding its *in vivo* efficacy and safety profile that this study aims to address.

In this study, we aim to systematically evaluate the antitumor effects of Thimerosal in solid malignancies, with a focus on CRC and melanoma, using comprehensive *in vitro* and *in vivo* approaches. We hypothesized that the antitumor properties of Thimerosal are mediated by its mercury ions, which inhibit the JAK1/STAT3 signaling pathway. To test this, a key objective is to investigate whether the mercury ions in the Thimerosal structure act as critical functional sites responsible for its antitumor properties. We also seek to examine its potential to inhibit the phosphorylation of Janus kinase 1 (JAK1) and signal transducer and activator of transcription 3 (STAT3). Additionally, we further hypothesized that Thimerosal would enhance the efficacy of anti-PD-1 therapy by promoting the infiltration of CD8^+^ T cells into the tumor microenvironment, thereby providing a novel combination strategy for cancer immunotherapy.

## 2 Materials and Methods

### 2.1 Materials

Thimerosal (C_9_H_9_HgNaO_2_S, CAS: 54-64-8, purity ≥99%, S3646, Selleck, Shanghai, China) was dissolved in Phosphate Buffer Saline (PBS) to prepare a 0.1 M stock solution. This stock solution was aliquoted and stored at −80°C, with a demonstrated stability of up to 6 months. For use, aliquots were thawed on ice and diluted with PBS (pH 7.2–7.4, 0.01 M) to the desired concentrations; repeated freeze-thaw cycles were avoided.

To evaluate the role of mercury ions in Thimerosal’s antitumor activity, two structural analogues, Sodium 2-((sec-butyl) thio) benzoate (CPD1) and Sodium 2-((phenyl) thio) benzoate (CPD2), were custom-synthesized by BirdoTech (Shanghai, China). These compounds were designed to replace the mercury-containing moiety with methyl or benzene groups while maintaining molecular spatial similarity. Both compounds showed purities >95%, as determined by high-performance liquid chromatography.

### 2.2 Cell Culture

Murine CRC cell lines (MC38, RRID: CVCL_B288; CT26, RRID: CVCL_7254), murine melanoma cell line (B16F10, RRID: CVCL_0159), human intestinal epithelial cell line (NCM460, RRID: CVCL_ 0460) and human CRC cell lines (SW620, RRID: CVCL_0547; SW480, RRID: CVCL_0546; LS-174T, RRID: CVCL_1384; DLD1, RRID: CVCL_0248; HCT8, RRID: CVCL_2478; RKO, RRID: CVCL_0504; HT29, RRID: CVCL_0320; LOVO, RRID: CVCL_0399; HCT15, RRID: CVCL_0292; HCT116, RRID: CVCL_0291) were obtained from the Cell Resource Center, Shanghai Institute of Biochemistry and Cell Biology at the Chinese Academy of Sciences (Shanghai, China) and were maintained in the Laboratory of Pathology, Southern Medical University (Guangzhou, China). Murine cell lines were cultured in DMEM medium (Thermo Fisher Scientific, Waltham, MA, USA), and human cell lines in RPMI-1640 medium (Thermo Fisher Scientific, Waltham, MA, USA), both supplemented with 10% fetal bovine serum (Thermo Fisher Scientific, Waltham, MA, USA) at 37°C, 5% CO_2_. All cell lines were used within 8 passages authenticated by short tandem repeat (STR) profiling and confirmed mycoplasma-free via monthly PCR testing.

### 2.3 Cell Proliferation and Viability Assay

For cell proliferation, 500 cells per well were seeded in 96-well plates and treated with Thimerosal (MC38 0.5 μM, CT26 1 μM, B16F10 0.1 μM, NCM460 1 μM) or its analogues CPD1 and CPD2 (CT26 1 μM, B16F10 0.1 μM) for up to 5 days. Cell viability was assessed every 24 h using Cell Counting Kit 8 (CCK-8, CK04, Dojindo, Kumamoto, Japan) with 1 h incubation, and absorbance was measured at 450 nm using a microplate reader (EnVision, PerkinElmer, Waltham, MA, USA). For viability assay, 800 cells per well (including MC38, CT26, B16F10, NCM460, SW620, SW480, LS-174T, DLD1, HCT8, RKO, HT29, LOVO, HCT15, and HCT116) were seeded in 96-well plates. Cells were treated with Thimerosal at varying doses (10, 5, 2.5, 1.25, 0.625, 0.3125, 0.1562, 0.078, 0.039, and 0.02 μM), using PBS as the control, for 48 h. After CCK-8 incubation, absorbance was measured at 450 nm. The half-maximal inhibitory concentration (IC_50_) was calculated by fitting dose-response data to a nonlinear regression model [log (inhibitor) vs. normalized response (Variable slope)] using GraphPad Prism software (Version 10.1, GraphPad Software, San Diego, CA, USA).

### 2.4 Colony Formation Assay

200 Cells per well were seeded in 6-well plates. Thimerosal was initially added at a specific concentration (CT26 1 μM, B16F10 0.1 μM, DLD1 1 μM, HCT15 1 μM). After 10–14 days, colonies formed by single cells were fixed with methanol (0.791 g/mL) and were stained with 0.1% crystal violet (Sigma, St. Louis, MO, USA) for 20 min. The plates were photographed (BX53, Olympus, Tokyo, Japan), and the number of colonies was quantified.

### 2.5 Cell Wound Healing Assay

Cells (CT26, B16F10, DLD1, HCT15) were seeded into 6-well plates and grown to 90% confluence. A scratch wound was created in each well using a 10 μL pipette tip. The wells were then gently washed twice with 2 mL of PBS to remove detached cells and debris. Migration was monitored by microscope (CKX31, Olympus, Tokyo, Japan) at 0, 24, 48, and 72 h, and image were captured.

### 2.6 Transwell Assay

4 × 10^4^ cells (CT26, B16F10, DLD1, HCT15) were seeded with 200 μL serum-free medium into 8.0-mm filter transwell upper chamber (Corning, Tewksbury, MA, USA), which was inserted into 24 well transwell plate. Medium containing 10% FBS was added to the bottom chamber. After 48 h of incubation, cells that migrated to the lower chamber were fixed with methanol (0.791 g/mL), stained with 0.1% crystal violet and counted under a microscope (BX53, Olympus, Tokyo, Japan) at 20× magnification. For transwell invasion assay, the chamber was pre-coated with Matrigel, and cells were incubated for 72 h.

### 2.7 ATP Cell Viability

1 × 10^4^ cells per well were seeded in 96-well plates and treated with Thimerosal at specified concentrations (MC38 0.5 μM, CT26 1 μM, B16F10 0.1 μM, DLD1 1 μM, HCT15 1 μM). After 24 h incubation, ATP levels were measured by the CellTiter-Glo kit (G7570, Promega, Shanghai, China). Briefly, luciferin and luciferase were added to cell lysates to react with ATP. The ATP levels in treated cells were normalized to those in control cells. Luminescence was measured using a Multiscan Spectrum microplate reader (Cytation5, Bio-Tek, Agilent, Winooski, VT, USA).

### 2.8 Western Blotting

Cells (MC38, CT26, B16F10, DLD1, HCT15) were treated with Thimerosal, CPD1, or CPD2 for 48 h, then lysed using SDS lysis buffer (KeyGEN BioTECH, Nanjing, China). Protein concentration was quantified with a BCA protein assay kit (23225, Pierce, Thermo Fisher Scientific, Waltham, MA, USA) in RIPA buffer. Equal amounts of protein were separated by 10% polyacrylamide gel electrophoresis and transferred to a PVDF membrane (Merck Millipore, Burlington, MA, USA). After blocking with 5% skim milk, the membrane was incubated with primary antibodies overnight at 4°C, followed by incubation with horseradish peroxidase (HRP)-conjugated secondary antibodies. Signals were detected using SuperSignal West Femto Chemiluminescent Substrate (34096, Thermo Fisher Scientific, Waltham, MA, USA), and protein bands were visualized with Image Lab Software (Tanon 5200, Shanghai, China).

Primary antibodies included: Anti-PARP (1:1000, 9532T, RRID: AB_659884, Cell Signaling Technology, Danvers, MA, USA); anti-Cleaved Caspase-3 (1:1000, 9664T, RRID: AB_2070042, Cell Signaling Technology, Danvers, MA, USA); anti-Caspase-3 (1:1000, 9662S, RRID: AB_331439, Cell Signaling Technology, Danvers, MA, USA); anti-α-tubulin (1:10,000, HRP-66031, RRID: AB_2687491, Proteintech, Chicago, IL, USA); anti-JAK1 (1:1000, 3344T, RRID: AB_2265054, Cell Signaling Technology, Danvers, MA, USA); anti-p-JAK1 (1:200, 3331S, RRID: AB_2265057, Cell Signaling Technology, Danvers, MA, USA); anti-STAT3 (1:1000, 12640, RRID: AB_2629499, Cell Signaling Technology, Danvers, MA, USA); anti-p-STAT3 (1:1000, 9145S, RRID: AB_2491009, Cell Signaling Technology, Danvers, MA, USA); anti-GAPDH (1:10,000, 60004-1-Ig, RRID: AB_2107436, Proteintech, Chicago, IL, USA).

### 2.9 Annexin-V/PI Apoptosis Analysis

Cancer cells treated with Thimerosal (MC38 0.5 μM, CT26 1 μM, DLD1 1 μM, HCT15 1 μM) for 48 h were harvested, washed, and resuspended in PBS. The cell suspension was centrifuged (5702R, Eppendorf, Hamburg, Germany) at 1500 rpm for 5 min; this washing step was repeated twice. Apoptosis was assessed using the Annexin V-FITC assay kit (KGA107, KeyGEN BioTECH, Nanjing, China) according to the manufacturer’s instructions. Cells were stained with Annexin V-FITC and propidium iodide for 10 min in the dark and analyzed by flow cytometry (BD, Franklin Lakes, NJ, USA).

### 2.10 Mice

Female C57BL/6, BALB/c and BALB/c nude mice (Guangdong Medical Laboratory Animal Center) aged 5–6 or 8 weeks were used. Mice were group-housed (4–5 mice per cage) in individually ventilated cages under specific pathogen-free conditions, with a 12-h light/dark cycle, and had adlibitum access to autoclaved water and standard irradiated rodent chow. All animal experiments were approved by the Animal Care and Use Committee of Southern Medical University (Approval No.: NFYY-2018-031; Date: 10 February 2018) and conducted in accordance with National Institutes of Health (NIH; Bethesda, MD, USA) guidelines. Humane endpoints were strictly observed beyond the primary tumor volume (≥2000 mm^3^) and ulceration. Animals were humanely euthanized immediately if they exhibited any of the following: (1) body weight loss exceeding 20% of the initial weight; (2) lethargy, anorexia, or hunched posture for >24 h; (3) inability to access food or water normally.

### 2.11 Subcutaneous Tumor Models

Initially, all tumor cells were resuspended in PBS (pH 7.2–7.4, 0.01 M). MC38 cells (1 × 10^6^/100 μL) were injected subcutaneously into the right flank of 5–6-week-old C57BL/6 or BALB/c nude mice. CT26 cells (1 × 10^6^/100 μL) were injected into BALB/c or BALB/c nude mice of the same age. B16F10 cells (5 × 10^5^/100 μL) were injected into the right flank of 5–6-week C57BL/6 mice. When the tumor volume reached 50–100 mm^3^, mice were randomly assigned to various experimental groups, including vehicle control and Thimerosal (CPD1, CPD2) treatment groups (with specific group sizes of n = 6–10 mice per group as detailed in the figure legends for each experiment). Mice received Thimerosal, CPD1, CPD2, anti-PD-1 antibody. Tumor size was measured using vernier calipers, and volume was calculated as 0.5 × length × width^2^. Mice were euthanized when volume tumor approached 2000 mm^3^ or upon observation of ulceration. Tumors were excised, recorded, fixed in 10% neutral buffered formalin (GHRHCH, Guangzhou, China), and embedded in paraffin.

### 2.12 B16F10 Pulmonary Metastasis Model

B16F10 cells (1 × 10^5^/100 μL) were injected intravenously via the tail vein into 5–6 weeks C57BL/6 mice. Thimerosal treatment was initiated on day 1. After 21 days, mice were euthanized, and lungs were harvested, rinsed in PBS, and placed on a white background. The number of surface metastatic nodules was counted manually under a dissecting microscope by an investigator blinded to group assignments. Lungs were then fixed in 10% neutral buffered formalin (GHRHCH, Guangzhou, China) for 24–48 h at room temperature for further histological analysis.

### 2.13 AOM/DSS Colitis-Associated CRC Model

8-week-old mice (Thimerosal n = 9 and PBS n = 8) were intraperitoneally injected (ip) with 5 mg/kg Azoxymethane (AOM, Sigma-Aldrich, St. Louis, MO, USA) on days 1 and 4, and received regular drinking water on days 1–7. Mice were then underwent three cycles of dextran sulfate sodium (DSS, MW 36,000–50,000 Da, MP Biomedical, Santa Ana, CA, USA) administration (1.8%, 2%, and 2%, each for 7 days) interspersed with 14-day periods of regular drinking water. After one month under standard conditions, mice were euthanized on day 101. Thimerosal (16.25 μg/kg) was administered during the late stage (after three DSS cycles). Colons were excised, and tumor number and size were recorded.

### 2.14 Hematoxylin and Eosin, Immunohistochemistry

Tissues obtained from the subcutaneous, lung metastasis and AOM/DSS colitis-associated CRC model described in Sections 2.11–2.13 were fixed, embedded, and sliced into 3 μm slices. The slices were stained using 0.5% hematoxylin and 1% eosin (H&E) (C0105S, Beyotime, Shanghai, China) stains. For immunohistochemistry, the slices were incubated with CD31 (1:100, 77699s, Cell Signaling Technology, Shanghai, China), anti-CD4 (1:1000, ab183685, Abcam, Shanghai, China), anti-CD8 (1:1000, ab209775, Abcam, Shanghai, China), and anti-Ki-67 (1:1000, ab21700, Abcam, Cambridge, UK) overnight at 4°C, appropriate secondary antibodies were applied. Finally, the slices were stained with DAB (ZLI-9019, Zsbio, Beijing, China) at a ratio of reagent A to B = 1:20 and counterstained with 0.5% hematoxylin.

### 2.15 Toxicity Assessment of Thimerosal In Vivo

To evaluate Thimerosal toxicity, 5–6 weeks old C57BL/6 mice were intraperitoneally injected with Thimerosal (16.25 μg/kg/qod) for a total of 10 administrations and then euthanized. The heart, liver and kidney were examined by H&E. For biochemical tests, serum levels of alanine aminotransferase (ALT), aspartate aminotransferase (AST), blood urea nitrogen (BUN), and creatinine (CREA) were measured using an Automatic Biochemical Analyzer (BS-240VET, Mindray, Shenzhen, China).

### 2.16 Treatment of Mice

In initial dose-finding studies, Thimerosal was administered at 4.0625, 8.125, 16.25, and 32.5 μg/kg, qod/ip. Based on the results, 16.25 μg/kg was selected for subsequent experiments. The following treatments were used: Thimerosal at 16.25 μg/kg. Its analogues CPD1 and CPD2 are at 16.25 μg/kg. Colivelin (TP1856, TargetMol, Shanghai, China), a synthetic peptide activator of the STAT3 signaling pathway, at 1 mg/kg, qod/ip. For combination therapy, 200 μg anti-PD-1 (BE0146, RMP1-14, BioXcell, Lebanon, NH, USA) or its Isotype control (BE0089, 2A3, BioXcell, Lebanon, NH, USA), diluted in PBS to a final volume of 100 μL, was administered intraperitoneally on days 8, 11, 13, 15, and 17.

### 2.17 RNA Sequencing and Bioinformatic Analysis

Total RNA was extracted from PBS-or Thimerosal-treated CT26 cells using TRIzol® Reagent (Invitrogen, Carlsbad, CA, USA) and assessed for purity, concentration, and integrity. Libraries were prepared from 1.3 μg total RNA using the TruSeq™ RNA Sample Prep Kit (Illumina) and sequenced on an Illumina HiSeq4000 (150-bp paired-end reads, ~40 million reads/sample).

After sequencing and quality control, clean reads were mapped to the mouse reference genome GRCm39 using HISAT2 (v2.2.0) [27], and gene expression levels were calculated using StringTie (v2.1.7) [28]. Differentially gene expression analysis was performed using DESeq2 (v1.44.0) [29] with significance thresholds set at |log__2__ fold change| > 1.5 and FDR < 0.05.

Functional enrichment analysis of differentially expressed genes was performed through Gene Ontology (GO) and Kyoto Encyclopedia of Genes and Genomes (KEGG) pathway analyses using the enrich GO and enrich KEGG functions from the clusterProfiler (v4.0) [30] R package.

### 2.18 Statistics

Data were presented as mean ± Standard Error of the Mean (SEM). The nonparametric two-tailed *t*-test was used for two-group comparisons; two-way ANOVA was applied for multi-group comparisons with two variables. The log-rank test was applied to analyze survival data. For the analysis of the distribution of tumor grades, the Chi-square test was applied. For *in vivo* studies, animals were randomly assigned to treatment groups, and tumor measurements were performed by an investigator blinded to the group allocation. All data were derived from at least three independent experiments. *p* < 0.05 was defined statistically significant.

## 3 Results

### 3.1 Thimerosal Inhibits Proliferation and Induces Apoptosis of Tumor Cells

**Figure 1:**
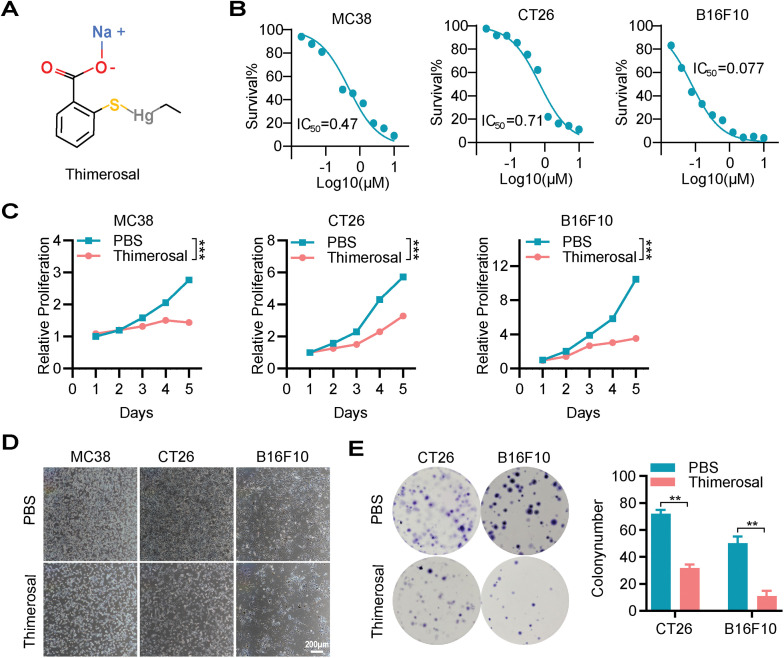

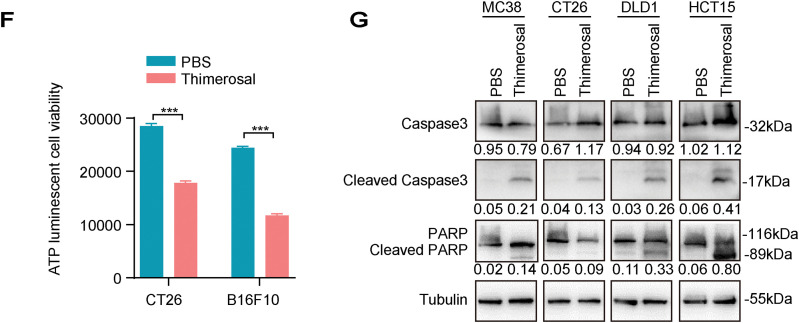
Thimerosal inhibits cell proliferation and induces apoptosis in cancer cells. (**A**) The chemical structure of Thimerosal. (**B**) Dose-response curves of Thimerosal in MC38, CT26, and B16F10 cells after 48 h of treatment; cell viability was determined by CCK-8 assay. (**C**) Viability of MC38, CT26, and B16F10 cells treated with Thimerosal over time, assessed by CCK-8 assay. (**D**) Representative microscopy images of MC38, CT26, and B16F10 cells treated with Thimerosal for 24 h. Scale bar: 200 μm. (**E**) Representative images and quantitative analysis of colony formation in CT26 and B16F10 cells (n = 3). (**F**) ATP content in CT26 and B16F10 cells after 24 h of Thimerosal treatment, measured by ATP assay (n = 4). (**G**) Apoptosis-related proteins in MC38, CT26, DLD1, and HCT15 cells after 48 h Thimerosal treatment, analyzed by Western blot. ((**C**–**G**): MC38 0.5 μM, CT26 1 μM, B16F10 0.1 μM, DLD1 1 μM, HCT15 1 μM). Data are expressed as mean ± SEM. **p < 0.01, ***p < 0.001

**Figure A1:**
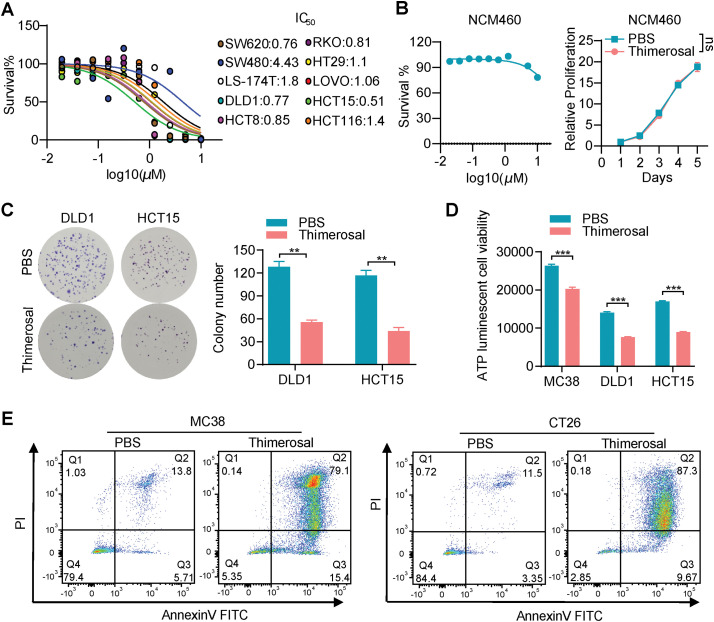

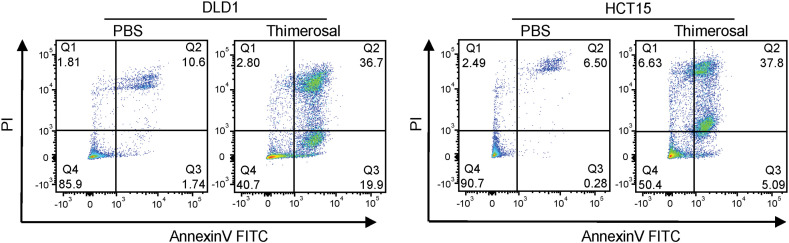
Thimerosal inhibits cell proliferation and induces apoptosis in cancer cells. (**A**) Dose–response curves for Thimerosal in CRC cell lines after 48 h treatment; cell viability was measured by CCK-8 assay. (**B**) CCK-8 assay was used to detect changes in dose–response curves and cell viability of normal colorectal epithelial cells (NCM460) treated with Thimerosal (48 h, days 1–5). (**C**) Representative images and quantitative analysis of colony formation in DLD1 and HCT15 cells (n = 3). (**D**) ATP content in MC38, DLD1, and HCT15 cells after 24 h of Thimerosal treatment, measured by ATP assay (n = 4). (**E**) Apoptosis in tumor cells treated with Thimerosal for 48 h, detected by Annexin V-FITC/PI staining. ((**C**–**E**): MC38 0.5 μM, CT26 1 μM, DLD1 1 μM, HCT15 1 μM). Data are expressed as mean ± SEM. ns: non-significant; **p < 0.01, ***p < 0.001

The chemical structure of Thimerosal is shown in Fig. 1A. To evaluate its cytotoxicity and inhibitory effects, mouse CRC cell lines MC38 and CT26, mouse melanoma cell line B16F10, and multiple human CRC cell lines were treated with Thimerosal. Cells were exposed to various concentrations for different durations, and viability was assessed using CCK-8 assay. Thimerosal inhibited tumor cell growth in a time-and dose-dependent manner (Figs. 1B,C and A1A). Importantly, this growth inhibition was not observed in normal human intestinal epithelial (NCM460) cells (Fig. A1B), indicating selective cytotoxicity. Thimerosal concentrations were selected based on IC_50_ values for each cell line. Microscopic examination further confirmed the growth-inhibitory effects, with treated cells showing reduced density and morphological changes indicative of impaired viability (Fig. 1D).

To further characterize the antiproliferative properties of Thimerosal, colony formation assays were performed. Thimerosal treatment significantly suppressed the colony-forming ability of tumor cells (Figs. 1E and A1C). This finding was supported by ATP assays, which revealed markedly reduced ATP levels in MC38, CT26, B16F10, DLD1, and HCT15 cells after treatment (Figs. 1F and A1D).

To determine whether growth inhibition was associated with apoptosis, cells were analyzed for apoptotic markers after 48 h of treatment. Flow cytometric analysis with Annexin V/PI staining showed a significant increase in the proportion of apoptotic cells (Fig. A1E). Western blot analysis demonstrated increased levels of cleaved caspase-3 and cleaved PARP (Fig. 1G). These results indicate that Thimerosal exerts potent antitumor effects by selectively inhibiting proliferation and inducing apoptosis.

### 3.2 Thimerosal Inhibits the Migration and Invasion of Tumor Cells

**Figure 2:**
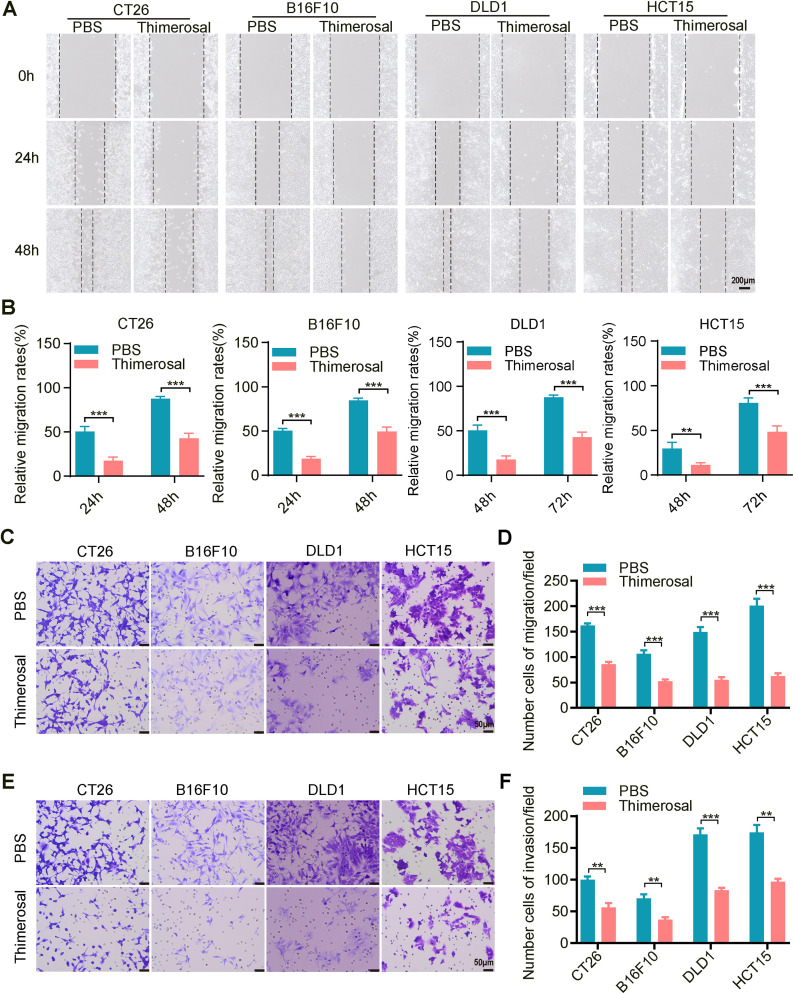
Thimerosal inhibits the migration and invasion of cancer cells. (**A**) Wound-healing assay of various tumor cells treated with Thimerosal. Scale bar: 200 μm. (**B**) Quantification of wound closure, shown as a percentage of initial scratch area. (**C**) Transwell migration assay of cells treated with Thimerosal after 48 h. Scale bar: 50 μm. (**D**) Number of migrated cells: The number of cells passing through the filtered membrane. (**E**) Matrigel-based Transwell invasion assay of cells treated with Thimerosal after 72 h. Scale bar: 50 μm. (**F**) Number of invaded cells.: The number of cells passing through the filtered membrane. ((**A**–**F)**: CT26 1 μM, B16F10 0.1 μM, DLD1 1 μM, HCT15 1 μM). Data are expressed as mean ± SEM. **p < 0.01, ***p < 0.001

Since cell migration and invasion are crucial for tumor metastasis, we evaluated the effects of Thimerosal using wound healing and Transwell assays. Thimerosal treatment significantly reduced the migratory capacity of tumor cells (Fig. 2A,B). Similarly, Transwell migration assays confirmed that Thimerosal markedly inhibited tumor cell migration (Fig. 2C,D). In Matrigel-coated Transwell assays, Thimerosal also effectively suppressed tumor cell invasion (Fig. 2E,F).

### 3.3 Thimerosal Demonstrates Potent Antitumor Activity and Safety In Vivo

**Figure 3:**
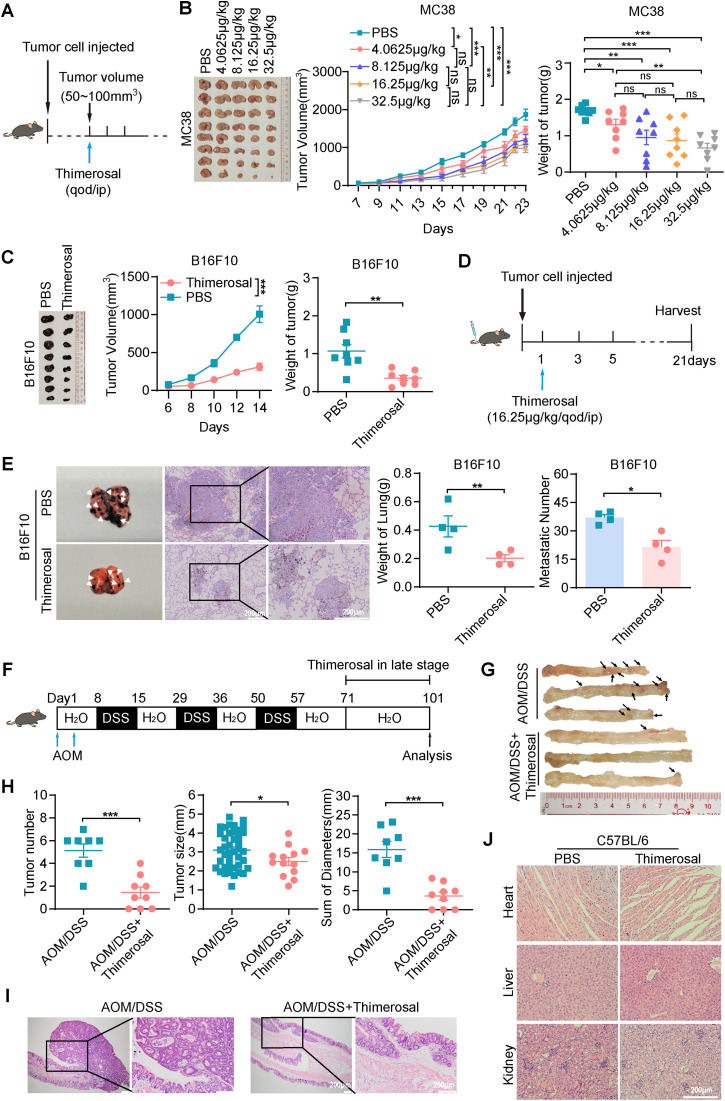
Thimerosal exerts antitumor efficacy on CRC and melanoma *in vivo*. (**A**) Therapy regimen schematic. C57BL/6J mice with MC38 subcutaneous tumors were administered with Thimerosal or PBS every 2 days (n = 8). (**B**) Images of MC38 subcutaneous tumor masses in C57BL/6J mice; corresponding tumor growth curves and tumor weights. (Thimerosal: 4.0625, 8.125,16.25, 32.5 μg/kg, qod/ip) (**C**) Images of B16F10 subcutaneous tumor masses in C57BL/6J mice; corresponding tumor growth curves and tumor weights. (n = 8). (**D**) Therapy regimen schematic for B16F10 lung metastasis model in C57BL/6J mice via tail vein injection, with Thimerosal or PBS administered every 2 days. (**E**) Representative images, weight and number of lung metastases for Thimerosal-treated on day 21 post-challenge (n = 4). White arrows indicate metastatic foci. Scale bar: 200 μm (applicable to all panels). (**F**) Schematic of AOM/DSS-induced colitis-associated CRC model. Thimerosal was administered in the late stage. Mice were euthanized on day 101 (Thimerosal n = 9 and PBS n = 8). (**G**) Representative images of colon tumors. Black arrows indicate tumors. (**H**) Number of tumors; size of tumors; sum of tumor diameters. (**I**) Representative H&E-stained colon sections. Scale bar: 200 μm. (**J**) Representative H&E-stained heart, liver, and kidney sections from mice treated with Thimerosal every 2 days (n = 7). Scale bar: 200 μm (applicable to all panels). ((**C**–**J**): Thimerosal 16.25 μg/kg, qod/ip). Data are expressed as mean ± SEM. ns: non-significant; *p < 0.05, **p < 0.01, ***p < 0.001

**Figure A2:**
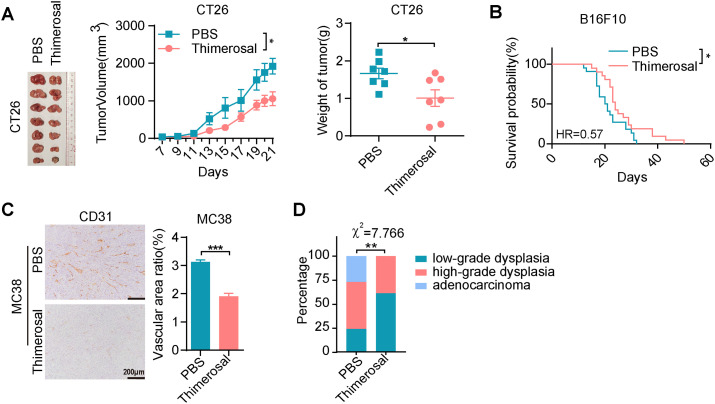
Thimerosal exerts antitumor efficacy on CRC and melanoma *in vivo*. (**A**) Representative images of CT26 subcutaneous tumors, tumor growth curves, and tumor weights in BALB/c mice (n = 7). (**B**) Kaplan–Meier survival curves of B16F10 lung metastasis-bearing C57BL/6J mice treated with PBS (n = 22) or Thimerosal (n = 21). ((**A**,**B**): Thimerosal 16.25 μg/kg, qod/ip). (**C**) CD31 histochemistry of MC38 subcutaneous tumor tissues; CD31-positive vascular area ratio. Scale bar: 200 μm. (**D**) Statistical distribution of tumor grades in the AOM/DSS model. Data are expressed as mean ± SEM. *p < 0.05, **p < 0.01, ***p < 0.001

To evaluate the therapeutic potential of Thimerosal *in vivo*, MC38 cells were subcutaneously injected into C57BL/6 mice. When tumor volumes reached approximately 50–100 mm^3^, mice were randomly divided into five groups: a control group (PBS) and four experimental groups receiving different doses of Thimerosal via intraperitoneal injection (Fig. 3A). Notably, Thimerosal treatment significantly suppressed the growth of MC38-derived subcutaneous tumors at both low and high doses, as indicated by reduced tumor volumes compared with the PBS control group (Fig. 3B). A dose of 16.25 μg/kg was selected for subsequent experiments. Similar suppression of tumor growth was observed in B16F10 and CT26 subcutaneous models (Figs. 3C and A2A).

Metastasis is a major cause of tumor progression and cancer-related mortality. We next evaluated the efficacy of Thimerosal in a lung metastasis model established by intravenous injection of B16F10 cells via the tail vein (Fig. 3D). Thimerosal treatment reduced lung weight, decreased the number of lung metastases (Fig. 3E), and improved survival rates (Fig. A2B). CD31 staining revealed significantly reduced angiogenesis in Thimerosal-treated MC38 tumors (Fig. A2C).

In an AOM/DSS-induced colitis-associated CRC model (Fig. 3F), Thimerosal significantly reduced both tumor number and cumulative diameter (Fig. 3G,H). Histopathological analysis showed that most tumors in the Thimerosal-treated group were adenomas with low-or high-grade intraepithelial neoplasia, whereas tumors in the AOM/DSS group consisted mainly of high-grade intraepithelial neoplasia or adenocarcinoma (Fig. 3I). Tumors were blindly categorized as low-grade dysplasia, high-grade dysplasia, or adenocarcinoma. The distribution of tumor grades differed significantly between PBS-and Thimerosal-treated groups, as determined by the chi-square test (Fig. A2D).

**Figure A3:**
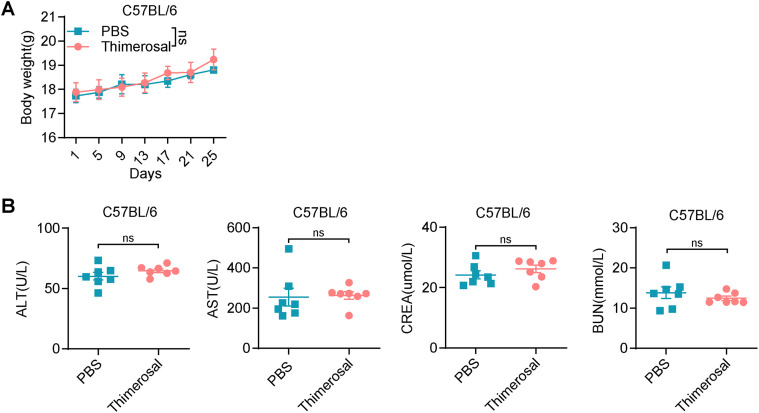
Thimerosal has no obvious toxicity in healthy C57BL/6 mice. (**A**) Body weight curves of C57BL/6J mice treated with PBS or Thimerosal every 2 days. (**B**) Serum levels of ALT, AST, BUN, and CREA (n = 7). (Thimerosal 16.25 μg/kg, qod/ip). Data are expressed as mean ± SEM. ns: non-significant

Safety assessment in healthy C57BL/6 mice revealed no significant body weight changes (Fig. A3A). Histopathological examination of major organs (heart, liver, and kidney) by H&E staining revealed no pathological abnormalities (Fig. 3J). Furthermore, serum levels of ALT, AST, BUN, and CREA remained within normal ranges, with no significant differences between Thimerosal-treated and control groups (Fig. A3B). These results demonstrate that Thimerosal exerts potent antitumor effects, suppressing tumor growth and metastasis in CRC and melanoma models, with minimal toxicity.

### 3.4 Mercury Ions Are Key Functional Sites for Thimerosal to Exert Antitumor Effects

**Figure 4:**
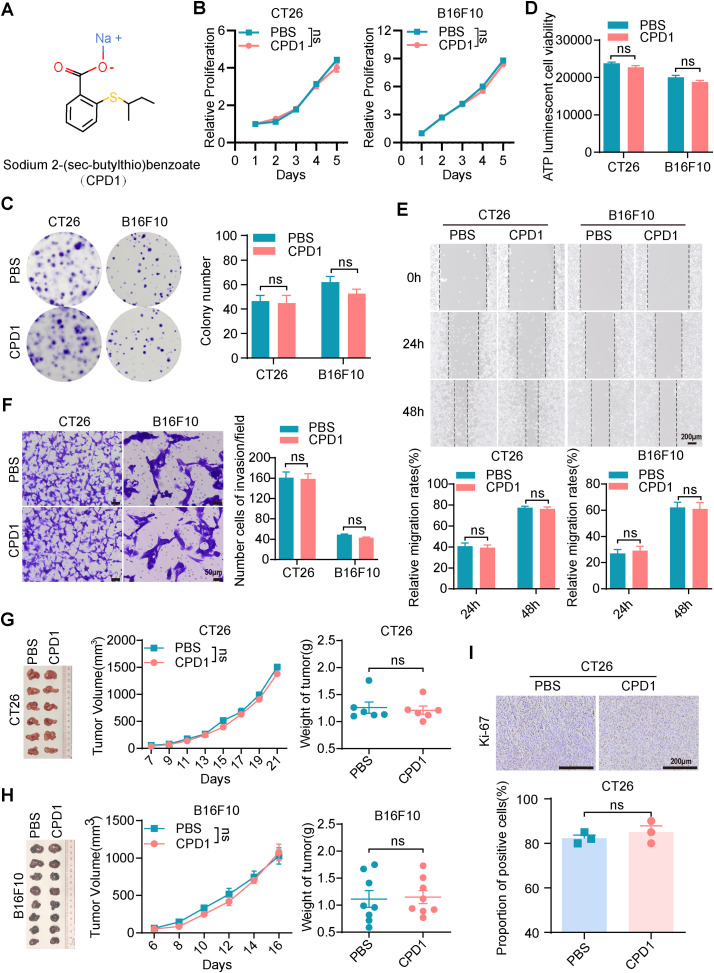
The molecular analogues of Thimerosal have no antitumor efficacy. (**A**) The chemical structure of CPD1, a molecular analogue of Thimerosal. (**B**) Viability of CT26 and B16F10 cells treated with CPD1 over time, measured by CCK-8. (**C**) Representative images and quantitative analysis of colony formation in CT26 and B16F10 cells (n = 3). (**D**) ATP content in CT26 and B16F10 cells after 24 h CPD1 treatment, measured by ATP assay (n = 4). (**E**) Wound-healing assay of CT26 and B16F10 cells treated with CPD1. Scale bar: 200 μm. (**F**) Matrigel-based Transwell invasion assay of cells treated with CPD1 after 48 h; number of invaded cells. Scale bar: 50 μm. (**G**) Images of CT26 subcutaneous tumors, tumor growth curves, and tumor weights in BALB/c mice (n = 6). (**H**) Images of B16F10 subcutaneous tumor masses, tumor growth curves and tumor weight in C57BL/6J mice (n = 8). (**I**) Ki-67 histochemistry of CT26 subcutaneous tumors. Scale bar: 200 μm. ((**B**–**F**): CT26 1 μM, B16F10 0.1 μM; (**G**,**H**): CPD1 16.25 μg/kg, qod/ip). Data are expressed as mean ± SEM. ns: non-significant

**Figure A4:**
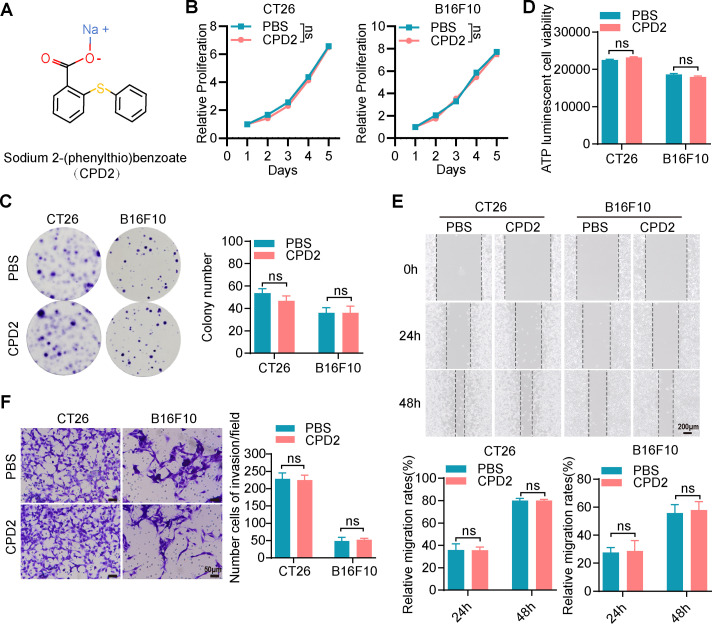

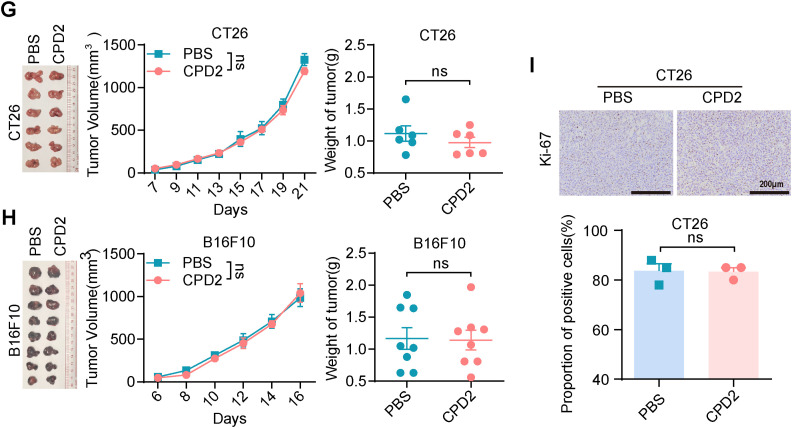
The molecular analogues of Thimerosal have no antitumor efficacy. (**A**) The chemical structure of CPD2, a molecular analogue of Thimerosal. (**B**) Viability of CT26 and B16F10 cells treated with CPD2 over time, measured by CCK-8. (**C**) Representative images and quantitative analysis of colony formation in CT26 and B16F10 cells (n = 3). (**D**) ATP content in CT26 and B16F10 cells after 24 h CPD2 treatment, measured by ATP assay (n = 4). (**E**) Wound-healing assay of CT26 and B16F10 cells treated with CPD2. Scale bar: 200 μm. (**F**) Matrigel-based Transwell invasion assay of cells treated with CPD2 after 48 h; number of invaded cells. Scale bar: 50 μm. (**G**) Images of CT26 subcutaneous tumors, tumor growth curves, and tumor weights in BALB/c mice (n = 6). (**H**) Images of B16F10 subcutaneous tumor masses, tumor growth curves and tumor weight in C57BL/6J mice (n = 8). (**I**) Ki-67 histochemistry of CT26 subcutaneous tumors. Scale bar: 200 μm. ((**B**–**F**): CT26 1 μM, B16F10 0.1 μM; (**G**,**H**): CPDjj2yjy 16.25 μg/kg, qod/ip). Data are expressed as mean ± SEM. ns: non-significant

Given the importance of metal ions in the biological activity of many drugs, we hypothesized that the mercury ions in Thimerosal were critical for its antitumor effects. To explore this, we utilized two structural analogues of Thimerosal (CPD1 and CPD2), in which the mercury group was replaced with an inert methyl group or benzene ring, respectively (Figs. 4A and A4A).

CCK-8 assays showed that CPD1 and CPD2 exerted no inhibitory effect on CT26 or B16F10 growth (Figs. 4B and A4B). Colony formation assays confirmed that both analogues failed to reduce colony numbers (Figs. 4C and A4C). ATP assays showed no significant differences in metabolic activity (Figs. 4D and A4D). Wound healing and Transwell assays demonstrated that neither analogue inhibited migration (Figs. 4E and A4E) or invasion (Figs. 4F and A4F).

In CT26 and B16F10 subcutaneous models, CPD1 and CPD2 showed no significant effects on tumor growth (Figs. 4G,H and A4G,H). Ki-67 staining revealed no significant difference in proliferation index (Figs. 4I and A4I). These results confirm that mercury ions are essential for Thimerosal’s antitumor activity.

### 3.5 Thimerosal Suppresses CRC and Melanoma Progression by Inhibiting JAK1-STAT3 Signaling Activation

**Figure 5:**
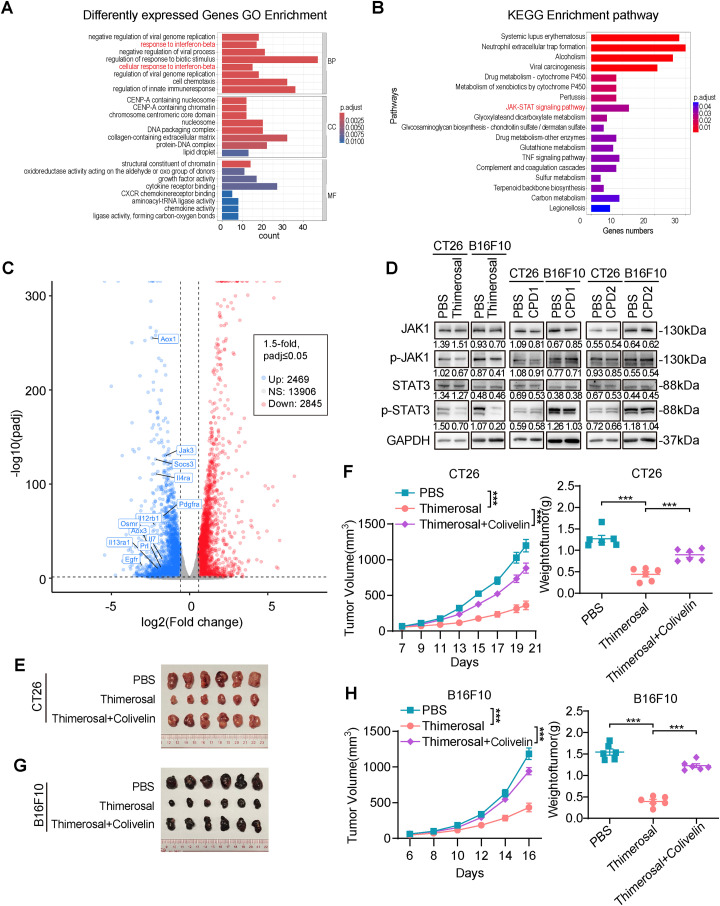
Thimerosal inhibits CRC and melanoma progression by inhibiting JAK1-STAT3 signaling activation. (**A**) GO Analysis of differently expressed genes in CT26 cells treated with PBS or Thimerosal (1 μM) for 48 h (n = 3). (**B**) KEGG analysis. (**C**) Volcano plot of differentially expressed genes. (**D**) Western blot analysis of JAK1, p-JAK1, STAT3, and p-STAT3 levels in CT26 (1 μM) and B16F10 (0.1 μM) cells after 48 h of Thimerosal treatment. (**E**) Images of CT26 subcutaneous tumors in BALB/c mice (n = 6). (**F**) CT26 subcutaneous tumor growth curves, and tumor weights in BALB/c mice (n = 6). (**G**) Images of B16F10 subcutaneous tumors in C57BL/6J mice (n = 6). (**H**) B16F10 subcutaneous tumor growth curves, and tumor weights in C57BL/6J mice (n = 6). ((**E**–**G**): Thimerosal 16.25 μg/kg, qod/ip, Colivelin 1 mg/kg, qod/ip.) Data are expressed as mean ± SEM. ***p < 0.001

To investigate the mechanism underlying the antitumor effects of Thimerosal, transcriptome analysis was performed to identify differentially expressed genes between Thimerosal-treated and untreated CT26 cells. GO analysis showed that these differentially expressed genes were predominantly associated with biological processes such as response to interferon-beta, regulation of response to biological stimulation, and regulation of innate immune response (Fig. 5A). KEGG pathway enrichment analysis and Volcano plot indicated significant enrichment in pathways related to drug metabolism, JAK-STAT signaling, and TNF signaling (Fig. 5B,C). Notably, the interferon response identified by GO enrichment was closely linked to the JAK-STAT signaling pathway identified by KEGG analysis. Given that the JAK-STAT pathway is a key downstream effector of interferon signaling and plays a pivotal role in antitumor responses, we hypothesized that this pathway might mediate Thimerosal’s antitumor effects.

Western blot analysis confirmed that Thimerosal decreased JAK1 and STAT3 phosphorylation (Fig. 5D). In contrast, CPD1 and CPD2 did not alter JAK1/STAT3 pathway activity. In subcutaneous tumor models, Thimerosal monotherapy significantly suppressed tumor growth, while combination with the STAT3 activator Colivelin restored tumor growth to control levels (Fig. 5E–H). This rescue effect supports the conclusion that Thimerosal acts through JAK1/STAT3 inhibition.

### 3.6 Thimerosal Increases CD8^+^ T Cell Infiltration in the Tumor Microenvironment

Thimerosal has been reported to exhibit antigen-stabilizing properties, and transcriptome analysis has linked its activity to interferon response-related genes and the JAK/STAT signaling pathway. Given that the JAK/STAT pathway serves as a crucial downstream effector of interferon signaling and plays a pivotal role in tumor immune responses, we investigated whether Thimerosal modulates the tumor immune microenvironment to influence tumor growth.

**Figure 6:**
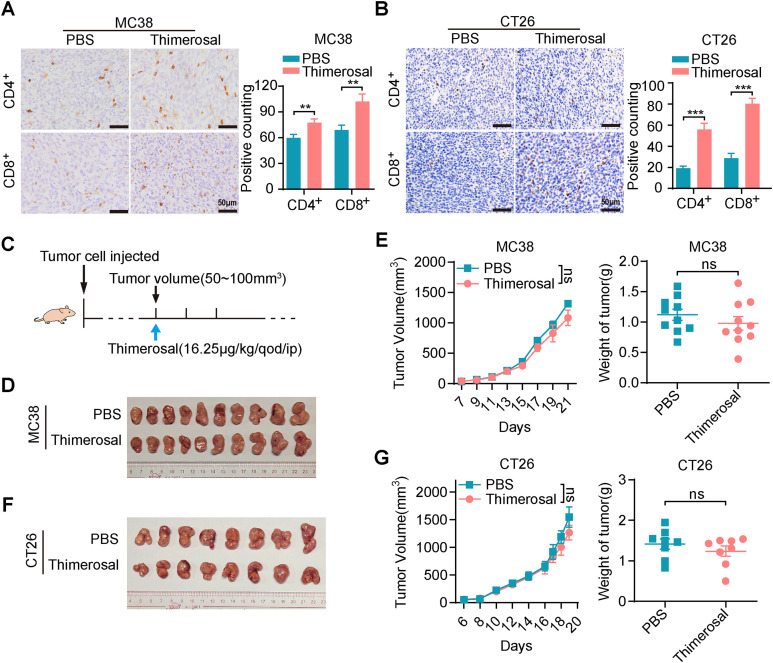
Thimerosal increases CD8^+^ T cell infiltration in the tumor microenvironment. (**A**) CD4/CD8 histochemistry of MC38 subcutaneous tumors. Scale bar: 50 μm. (**B**) CD4/CD8 histochemistry of CT26 subcutaneous tumors. Scale bar: 50 μm. (**C**) Therapy regimen schematic. BALB/c nude mice with subcutaneous tumors were administered Thimerosal or PBS every 2 days. (**D**) Images of MC38 subcutaneous tumors in BALB/c nude mice (n = 10). (**E**) MC38 subcutaneous tumor growth curves, and tumor weights in BALB/c nude mice (n = 10). (**F**) Images of CT26 subcutaneous tumors in BALB/c nude mice (n = 8). (**G**) CT26 subcutaneous tumor growth curves, and tumor weights in BALB/c nude mice (n = 8). ((**D**–**G**): Thimerosal 16.25 μg/kg, qod/ip). Data are expressed as mean ± SEM. ns: non-significant; **p < 0.01, ***p < 0.001

Immunohistochemical analysis revealed significantly increased infiltration of CD4^+^ and CD8^+^ T cells in MC38 and CT26 tumors after Thimerosal treatment (Fig. 6A,B). To further evaluate the role of T cell infiltration in Thimerosal-mediated antitumor effects, a subcutaneous tumor model was established in BALB/c nude mice, which lack functional T cells (Fig. 6C). Thimerosal treatment resulted in a modest reduction in tumor growth rate and tumor weight in MC38 subcutaneous tumors in nude mice compared to the control group (Fig. 6D,E). However, the antitumor efficacy was markedly reduced compared to that observed in immune-competent C57BL/6 mice. Similarly, Thimerosal treatment partially inhibited the growth of CT26 subcutaneous tumors in nude mice, but the effects were less pronounced than those in immune-competent BALB/c mice (Fig. 6F,G). These results indicate that Thimerosal enhances the infiltration of CD4^+^ and CD8^+^ T cells in the tumor microenvironment, which contributes to its antitumor efficacy.

### 3.7 Thimerosal in Combination with Anti-PD-1 Exhibits Enhanced Anti-Tumor Efficacy

**Figure 7:**
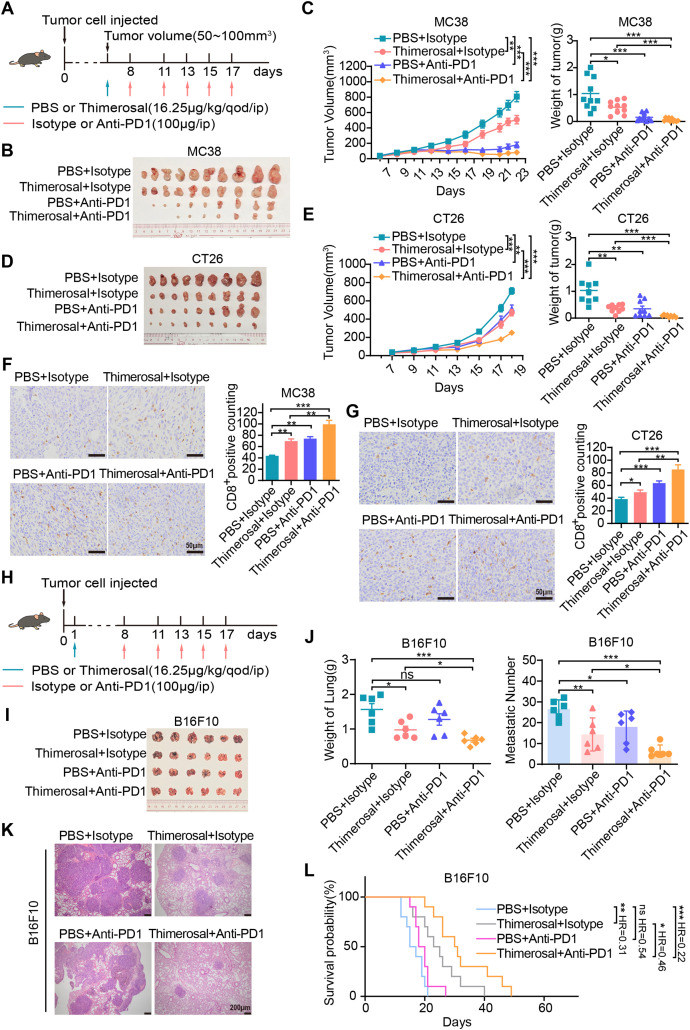
Thimerosal enhances the antitumor efficacy of anti-PD-1 therapy. (**A**) Therapy regimen schematic for (**B**–**G**). Mice with subcutaneous tumors were administered Thimerosal and/or anti-PD-1. (**B**) Images of MC38 subcutaneous tumors in C57BL/6J mice (n = 10). (**C**) MC38 subcutaneous tumor growth curves, and tumor weights in C57BL/6J mice (n = 10). (**D**) Images of CT26 subcutaneous tumors in BALB/c mice (n = 9). (**E**) CT26 subcutaneous tumor growth curves, and tumor weights in BALB/c mice (n = 9). (**F**) CD8 histochemistry of MC38 subcutaneous tumors. Scale bar: 50 μm. (**G**) CD8 histochemistry of CT26 subcutaneous tumors. Scale bar: 50 μm. (**H**) The flowchart of the tail vein injection protocol in C57BL/6J mice with B16F10 metastasis tumors was administered Thimerosal and/or anti-PD-1. (**I**) Images of B16F10 lung metastases in C57BL/6J mice in the four treatment groups on day 21 post-challenge (n = 6). (**J**) Lung weights and metastatic nodules of B16F10 lung metastases in C57BL/6J mice in the four treatment groups on day 21 post-challenge (n = 6). (**K**) HE Images of B16F10 lung metastases in C57BL/6J mice. Scale bar: 200 μm. (**L**) Kaplan–Meier survival curves of B16F10 lung metastasis-bearing C57BL/6J mice treated with Thimerosal and/or anti-PD-1 (n = 10). (Thimerosal 16.25 μg/kg, qod/ip; anti-PD-1 200 μg/ip). Data are expressed as mean ± SEM. ns: non-significant; *p < 0.05, **p < 0.01, ***p < 0.001

We evaluated the combination of Thimerosal with anti-PD-1 in subcutaneous tumor models. Both monotherapies suppressed tumor growth, while the combination showed enhanced efficacy, achieving complete tumor regression in 3 of 10 MC38-bearing mice (Fig. 7A–C). Similar synergy was observed in CT26 models, with the combination being more effective than either agent alone (Fig. 7D,E). Immunohistochemical analysis revealed that Thimerosal promoted the infiltration of CD8^+^ T cells within the tumor stroma (Fig. 7F,G).

The antitumor efficacy of the combination was further evaluated in a B16F10 lung metastasis model established via tail vein injection (Fig. 7H). Both Thimerosal and anti-PD-1 monotherapies partially suppressed metastatic tumor growth. In contrast, the combination therapy significantly reduced lung tumor burden, including metastatic growth and number (Fig. 7I–K), and prolonged the survival time of treated mice (Fig. 7L). These results indicate that the combination of Thimerosal and anti-PD-1 enhances antitumor immunity and improves therapeutic outcomes in both subcutaneous and metastatic tumor models.

## 4 Discussion

In this study, we provide compelling evidence that Thimerosal, an organic compound commonly used as a preservative in vaccines, exhibits significant antitumor efficacy in CRC and melanoma. Using integrated *in vitro* and *in vivo* experimental approaches, we showed that Thimerosal effectively inhibits tumor cell proliferation, invasion, and metastasis, while also enhancing antitumor immune responses. Mechanistically, Thimerosal was found to inhibit the JAK1/STAT3 signaling pathway, which is frequently dysregulated in cancer and promotes tumor progression, metastasis, and immune evasion. This inhibition not only suppresses malignant phenotypes but also promotes immune activation within the tumor microenvironment. Given the established crosstalk between the JAK/STAT pathway and other oncogenic cascades such as PI3K/Akt, future studies should investigate whether Thimerosal’s antitumor effects also involve modulation of these interconnected networks [31]. Notably, Thimerosal exhibits synergistic effects when combined with the immune checkpoint inhibitor anti-PD-1. These findings highlight the dual mechanisms of Thimerosal: direct inhibition of tumor cell signaling pathways and modulation of the tumor microenvironment. This dual functionality supports its potential clinical application in overcoming resistance to immune checkpoint blockade by simultaneously targeting tumor-intrinsic survival pathways and the immunosuppressive microenvironment, addressing a critical limitation in current cancer therapy.

A key discovery is the identification of mercury ions as critical functional sites. Metal ions play vital roles in the function of many bioactive compounds [32–34]. For instance, manganese ions potently activate the STING pathway, converting immunologically “cold” tumors into “hot” tumors [35], and copper ions are essential for tumorigenic signaling in BRAF V600E mutations [36]. Zinc-mediated metalloimmunotherapy enables dual elimination of tumors and intratumoral bacteria in oral squamous cell carcinoma [37]. In this context, we identified mercury ions as crucial for Thimerosal’s antitumor effects. When mercury ions were replaced with inert groups in the molecular structure, the resulting analogues showed no inhibitory effects on malignant phenotypes *in vitro* or *in vivo*. This clearly establishes mercury ions as essential functional components of Thimerosal’s antitumor activity. Based on these findings and mercury’s known high affinity for cysteine thiol groups, we propose a mechanistic hypothesis whereby mercury ions may directly bind to critical cysteine residues within the kinase domain of JAK1. This interaction could disrupt the redox-sensitive enzymatic activity of JAK1, potentially through allosteric inhibition or interference with ATP binding, ultimately leading to inhibition of phosphorylation. This potential direct targeting of JAK1 represents a distinct mechanism from the ROS/Ca²^+^-mediated cell death previously reported for Thimerosal in other cancer types. The differential mechanisms may reflect cell type-specific signaling contexts or differential expression of molecular targets, suggesting that Thimerosal may engage varied pathways in different malignancies. Although controversies regarding its safety persist due to the neurotoxicity of mercury [38], extensive investigations by regulatory authorities have concluded that there is no evidence of harm caused by Thimerosal-containing vaccines [39,40]. These formulations remain in use, offering the advantage of improved vaccine stability [41,42]. Consistent with these findings, our study demonstrated that Thimerosal exhibits significantly higher cytotoxicity against tumor cells than against normal intestinal epithelial cells. Moreover, we confirmed the safety of Thimerosal in mice through serum biochemical indices and histological examination of key organs.

*In vitro*, Thimerosal effectively inhibited tumor cell proliferation and invasion and induced apoptosis. The suppression of migration and invasion may result from interference with epithelial-mesenchymal transition, a process modulated by various non-coding RNAs [43]. Its therapeutic efficacy was further validated *in vivo* across multiple mouse models, including MC38, CT26, and B16F10-derived subcutaneous tumors, B16F10 lung metastases, and AOM/DSS-induced colitis-associated CRC. These results highlight the clinical significance of Thimerosal in treating CRC and melanoma. Compared with previous studies, our work is the first to confirm Thimerosal’s inhibitory effects on solid tumors using diverse animal models.

Mechanistically, transcriptomic analyses revealed significant dysregulation of the JAK/STAT signaling pathway in Thimerosal-treated CT26 cells. This pathway is implicated in various cancers [44,45], including hepatocellular carcinoma [46] and breast cancer [47], where it enhances metastasis and inhibits autophagy. Inhibiting JAK2/STAT3 signaling has been shown to block malignant progression, and numerous studies have supported targeting STAT3 activation as a therapeutic strategy [48]. Our results demonstrated that Thimerosal inhibits JAK1/STAT3 phosphorylation in CT26 and B16F10 cells, providing a mechanistic basis for its antitumor effects. Given the established role of JAK/STAT signaling in regulating cellular metabolism [49], its inhibition by Thimerosal may potentially influence metabolism-related cell death pathways, such as ferroptosis. This potential metabolic reprogramming, combined with its immunomodulation, could create a favorable tumor microenvironment that is highly susceptible to anti-PD-1 therapy, providing a rational basis for their synergistic combination.

Immunotherapy has become a cornerstone for treating CRC and melanoma, but many tumors remain resistant due to an immunosuppressive microenvironment [50–52]. Our findings showed that Thimerosal increases CD8^+^ T cell infiltration in the tumor microenvironment, markedly enhancing the efficacy of PD-1 blockade. Strong evidence links high CD8^+^ T cell infiltration with favorable clinical outcomes [53]. In our study, the combination of Thimerosal and anti-PD-1 produced synergistic effects, reducing tumor growth and prolonging survival in CRC and melanoma models, including B16F10 lung metastases. However, the mechanism by which Thimerosal enhances CD8^+^ T cell infiltration remains unclear. Given that the JAK/STAT3 pathway negatively regulates antitumor immunity, it is plausible that Thimerosal promotes CD8^+^ T cell infiltration via modulation of this pathway, which merits further investigation.

However, it is important to acknowledge the limitations of this study and the translational challenges ahead. First, while our findings demonstrate efficacy across multiple murine models, the translational relevance to human cancers remains to be established due to the lack of data from patient-derived models, such as patient-derived xenografts or organoids. Second, while short-term safety was confirmed at the therapeutic dose, potential long-term or neurotoxic effects with chronic administration were not evaluated, a critical consideration given mercury’s known neurotoxicity at high doses. Third, off-target effects mediated by mercury ions and mechanisms of acquired resistance were beyond the scope of this study. Despite these limitations, the repurposing potential of Thimerosal is considerable due to its novel dual mechanism of action. To address the legitimate concerns regarding its mercury content, future work should prioritize the development of mercury-free analogs that retain its immunomodulatory and JAK-STAT inhibitory properties. Furthermore, initial clinical translation could explore local administration strategies (e.g., intratumoral injection) in phase I trials to minimize systemic exposure while rigorously evaluating safety in oncology patients.

## 5 Conclusions

In summary, Thimerosal demonstrated dual antitumor roles by directly inhibiting tumor cell proliferation and metastasis while modulating the immune microenvironment to enhance CD8^+^ T cell infiltration. Mercury ions were identified as indispensable to its mechanism of action, and JAK1/STAT3 signaling emerged as a key target. Importantly, Thimerosal significantly improved the efficacy of PD-1 blockade therapy in CRC and melanoma models, suggesting its potential as an adjunct to immunotherapy. Given its established safety profile in vaccines, Thimerosal represents a promising candidate for rapid clinical translation in cancer therapy, particularly for CRC and melanoma. This study provides a foundation for future investigations into the clinical and mechanistic aspects of Thimerosal’s antitumor activity.

## Supplementary Materials







## Data Availability

The data that support the findings of this study are available from the corresponding author on reasonable request. The RNA-seq data generated in this study have been deposited in the NCBI Gene Expression Omnibus (GEO) database under accession number GSE307702.

## Supplementary Materials

The supplementary material is available online at https://www.techscience.com/doi/10.32604/or.2025.071092/s1.

## Abbreviations

CRC: Colorectal cancer
JAK1: Janus kinase 1
p-JAK1: Phosphorylation of JAK1
STAT3: Signal transducer and activator of transcription 3
p-STAT3: Phosphorylation of STAT3
ip: Intraperitoneal Injections
AOM: Azoxymethane
DSS: Dextran sulfate sodium
H&E: Hematoxylin and eosin stains
CPD1: Sodium 2-((sec-butyl) thio) benzoate
CPD2: Sodium 2-((phenyl)thio) benzoate
GO: Gene Ontology Enrichment Analysis
KEGG: Kyoto Encyclopedia of Genes and Genomes.
