# Supplementary material for: Thimerosal Inhibits Tumor Malignant Progression through Direct Action and Enhancing the Efficacy of PD-1-Based Immunotherapy
Source: Oncol Res. 2026 Jan 19;34(2):20. doi: 10.32604/or.2025.071902 (PMC12848756; doi:10.32604/or.2025.071902)
Supplement: Supplementary file 1 [file OncolRes-34-71902-s001.zip › OR_71902-s001/B16F10-STR.pdf]

Cell Line Authentication Service

STR Profiling Report

Sample Frome: Southern Medical University

Sample Type: Cell Line

Testing Method: STR Genotyping

Report Time: January 16, 2025

Add: Room 518, No.2, Zhongxing Creative Park, Lane 1015, Longteng Road, Songjiang District, Shanghai / Tel:+86-18521538068

COMPANY STATEMENT

1. THIS REPORT IS ONLY RESPONSIBLE FOR THE SAMPLES ANALYZED.
2. THE TESTING RESULTS AND THE ORGANIZATION NAME WILL NOT BE USED FOR ADVERTISEMENT, COMMERCIAL EXHIBITIONS, COMMERCIAL PERFORMANCE AND OTHER COMMERCIAL ACTIVITIES.
3. OBJECTIONS SHOULD BE RAISED WITHIN FIFTEEN DAYS AFTER THE RECEIPT OF THIS REPORT.
4. THE PAPER REPORT WITH CONTENT ALTERING, ADDING OR WITHOUT THE STAMPED SEAL OF THE COMPANY ARE INVALID.

Testing Company: Shanghai Biowing Applied Biotechnology Co. Ltd

Address: Room 502, NO.1015 Longteng Rd, Songjiang District, Shanghai

Tel: +86-18521538068

Contact: Shuangning Zhu

E-mail: zhusn@biowing.com.cn

Cell Line Authentication – STR Profiling Report

Sample code

Table 1. Sample Code

Customer's code	Company Code
B16-F10	20250113-03

Sample Number:1

Sample Type: Cell line

Testing Type: STR

Testing Method:

DNA was extracted by a commercial kit from CORNING (AP-EMN-BL-GDNA-250G). The ten STRs including one human locus were amplified by multiplex PCR and separated on ABI 3730XL Genetic Analyzer. The signals were then analyzed by the software GeneMapper..

Data Interpretation:

Cell lines were authenticated using Short Tandem Repeat (STR) analysis asdescribed in 2021 in ANSI Standard (ASN-0002) by the ATCC Standards Development Organization (SDO) and in Capes-Davis et al., Match criteria for human cell line authentication: Where do we draw the line? Int J Cancer.2013;132(11):2510-9.

DSMZ tools was used to carry on the cell line comparison, which contains >8235 cell lines STR data from ATCC, DSMZ, JCRB, ECACC, GNE, RIKEN and EXPASY databases. If the cell is not included in the above cell library, users need to compared with other databases.

Add: Room 518, No.2, Zhongxing Creative Park, Lane 1015, Longteng Road, Songjiang District, Shanghai / Tel:+86-18521538068

Test Results

1. STR profile

Table 2. STR and Amelogenin Genotyping Results of Cell line.

Loci	Sample information				Cell Bank information		
	Sample name： B16-F10				Cell line name： B16-F10		
	Allele1	Allele2	Allele3	Allele4	Allele1	Allele2	Allele3
18-3	15	16			15	16	
4-2	20.3	21.3			20.3	21.3	
6-7	15				15		
19-2	13				13		
1-2	19	20			19	20	
7-1	26.2				26.2		
8-1	16	17			16	17	
1-1	17	18			17	18	
3-2	14	15			14	15	
2-1	16				16		
15-3	22.3	23.3			22.3	23.3	
6-4	18	19			18	19	
13-1	17.1	18.1			17	18	
11-2	16	17			16	17	
17-2	15	16	17		15	16	17
12-1	17	18			17	18	
5-5	16	20			16	20	
X-1	15	16			15	16	
TH01							
D4S2408							

Add: Room 518, No.2, Zhongxing Creative Park, Lane 1015, Longteng Road, Songjiang District, Shanghai / Tel:+86-18521538068

2. database annotation

Figure 1. STR matching analysis

Accession	Name	Nº Markers	Score	STR 1-1	STR 1-2	STR 2-1	STR 3-2	STR 4-2	STR 5-5	STR 6-4	STR 6-7	STR 7-1	STR 8-1	STR 11-2	STR 12-1	STR 13-1	STR 15-3	STR 17-2	STR 18-3	STR 19-2	STR X-1
NA	Query	NA	NA					20.3,21.3	16,20	18,19	15				17,18		22.3,23.3		15,16		28
CVCL_0158	B16-F1	8	100.00%	17,18	19,20	16	14,15	20.3,21.3	16,20	18,19	15	26.2	16,17	16,17	17,18	17,18	22.3,23.3	15,16,17	15,16	13	28
CVCL_0159	B16-F10	8	100.00%					20.3,21.3	16,20	18,19	15				17,18		22.3,23.3		15,16		28

Note: The STR online match analysis of the test cell against EXPASY database, showing cell number (Cell No.) and cell name.

3. Authentication

☐ The submitted sample profile is mouse, but not a match for any profile in the EXPASY.

☑ The submitted profile is exact match for the following mouse cell line(s) in the EXPASY STR database (8 core loci plus Amelogenin): **B16-F10**.

☐ The submitted profile is similar to the following EXPASY mouse cell line: /.

- **Note:** Cell lines are considered to related, derived from a common ancestry, when >80% of the alleles in its STR profile match profiles from tissue or other cell line samples from that donor or from database. Cell lines with between a 55% to 80% (similar) match require further profiling for investigation of relatedness.

Appendix:

1. Genotyping Strategy and Site Distribution

Table S1. Experimental Strategy and Sites

	Pannal1	Pannal2	Pannal3	Pannal4
1	18-3	1-2	2-1	TH01
2	4-2	7-1	15-3	D5S818
3	6-7	8-1	6-4	17-2
4	19-2	1-1	13-1	12-1
5		3-2	11-2	5-5
6				X-1

The allele match algorithm compares the 18 core loci only, D5S818, TH01 is a human site, which is used to detect whether the cell is contaminated by human sources.

Shanghai Biowing Applied Biotechnology Co. Ltd
STR Profiling
Cell Line Authentication

Technician: Xiuchuan He

Checked by: Chenqian Zhang

Issued by: Min Wang

Issue date: January 16, 2025

Add: Room 518, No.2, Zhongxing Creative Park, Lane 1015, Longteng Road, Songjiang District, Shanghai / Tel:+86-18521538068

Figure 2. STR profiles of sample cell line

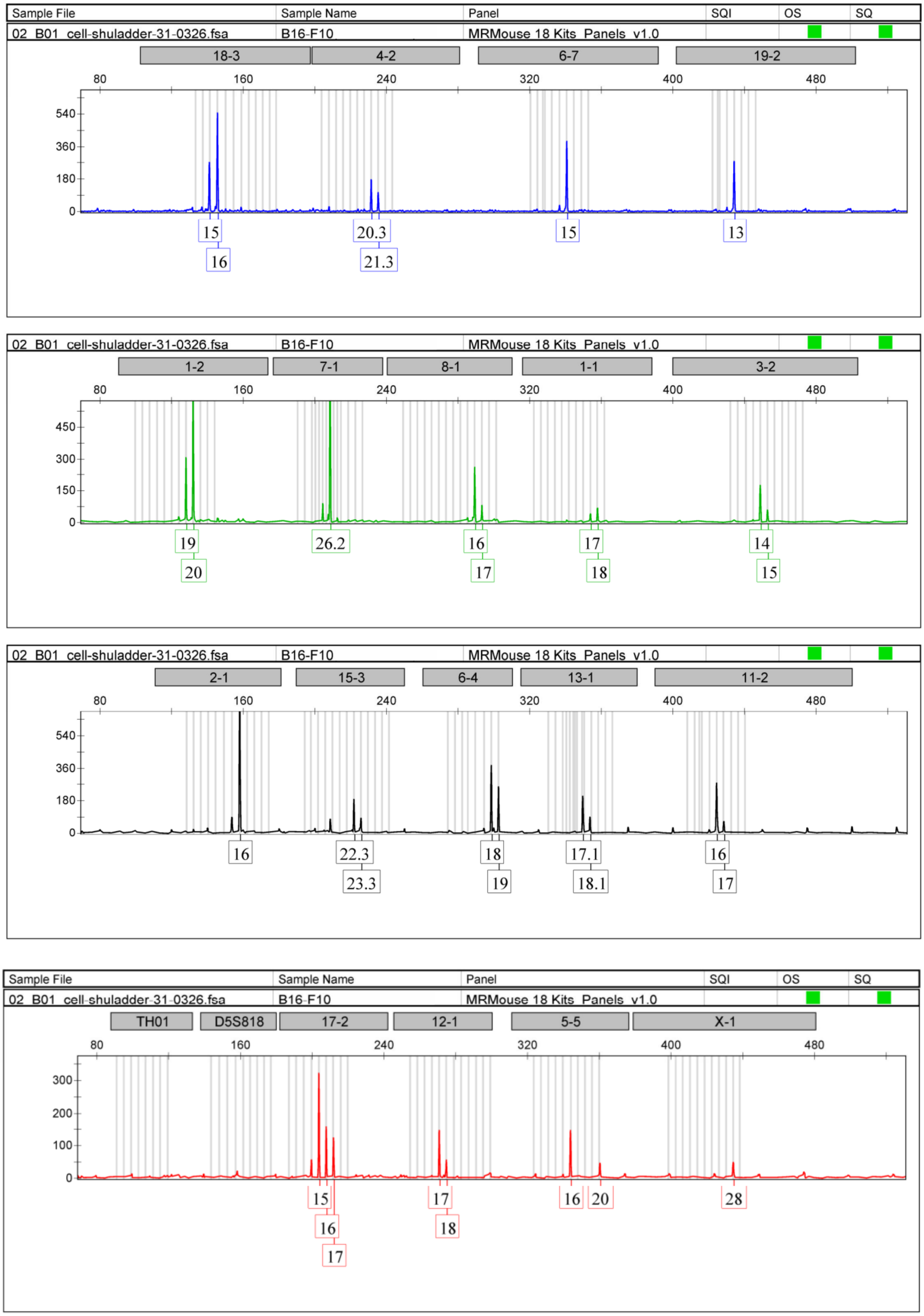

Add: Room 518, No.2, Zhongxing Creative Park, Lane 1015, Longteng Road, Songjiang District, Shanghai / Tel:+86-18521538068
