# Supplementary material for: Thimerosal Inhibits Tumor Malignant Progression through Direct Action and Enhancing the Efficacy of PD-1-Based Immunotherapy
Source: Oncol Res. 2026 Jan 19;34(2):20. doi: 10.32604/or.2025.071902 (PMC12848756; doi:10.32604/or.2025.071902)

细胞遗传质量鉴定检测

Cell Line Authentication Service

STR 基因型检测报告

送检编号： HCT-8

检品名称： 细胞系

报告编号： NO.20220307-STR06

报告日期： 2022-03-07

Cell Line Authentication and Identification Service 邮箱：18521538068@163.com 服务热线：86+18521538068
联系地址：上海市松江区广富林街龙腾路 1015 弄 2 号楼 518 室

报 告 说 明

1. 本报告只对送检的来样负责。
2. 检验报告上的检验结果和检验单位名称，未经同意不得用于广告、评优及商业宣传。
3. 对本报告有异议，请于收到报告之日起十五日内以书面方式提出，逾期不予受理。
4. 对纸质检验报告涂改、增删，或未加盖检验单位印章的复印件均无效。

Cell Line Authentication and Identification Service 邮箱：18521538068@163.com 服务热线：86+18521538068
联系地址：上海市松江区广富林街龙腾路 1015 弄 2 号楼 518 室

样 品 信 息

样品编号：

客户样本编号	公司编号
HCT-8	20220302-01

样品数量： 1

样品性状： 细胞系

检测项目： STR

检测方法： 用 Axygen 的基因组抽提试剂盒提取 DNA，采用 20- STR 扩增方案扩增，在 ABI 3730XL 型遗传分析仪上对 STR 位点和性别基因 Amelogenin 进行检测。

Cell Line Authentication and Identification Service 邮箱：18521538068@163.com 服务热线：86+18521538068
联系地址：上海市松江区广富林街龙腾路 1015 弄 2 号楼 518 室

检 测 结 果

(一) 检验基本情况

	多等位基因	匹配细胞系	细胞库	EV 值	匹配说明
20220302-01	无	HCT-8	EXPASY	1.00	完全匹配

样本基因型检验结果

- 多等位基因指三等位及以上基因现象。
- 本次检测各细胞分型结果良好。

(二) 各样本描述

- 20220302-01：该株细胞 DNA 分型在细胞系检索中找到<u>**完全匹配**</u>的细胞系，EXPASY 数据库显示细胞名为 <u>**HCT-8**</u>，细胞号对应 <u>**CVCL_2478**</u>。本次检测在该细胞系中<u>**没有发现多等位基因**</u>。

EV	Cell No.	Cell name	Locus names									Figures
			D5S818	D13S317	D7S820	D16S539	VWA	TH01	AM	TPOX	CSF1PO	
	Query (Your Cell)		*13,13*	*8,11*	*10,12*	*12,13*	*18,19*	*7,9.3*	*X,Y*	*8,11*	*12,12*	
1.00(36/36)	357	HCT-15	13,13	8,11	10,12	12,13	18,19	7,9.3	X,Y	8,11	12,12	-
1.00(36/36)	CCL-225	HCT-15	13,13	8,11	10,12	12,13	18,19	7,9.3	X,Y	8,11	12,12	-

备注：待测细胞系与收录于 ATCC, DSMZ, JCRB 和 RIKEN 数据库的细胞系 STR 数据进行比对，未收录于以上细胞库的细胞系将无法匹配。

（三）样本分型结果

细胞 20220302-01 的 STR 位点和 Amelogenin 位点的基因分型结果						
Loci	送检细胞 STR 信息			细胞库细胞 STR 信息		
	送检细胞名：HCT-8			细胞库细胞名：HCT-8		
	Allele1	Allele2	Allele3	Allele1	Allele2	Allele3
D5S818	13	13		13	13	
D13S317	8	11		8	11	
D7S820	10	12		10	12	
D16S539	12	13		12	13	
VWA	18	19		18	19	
TH01	7	9.3		7	9.3	
AMEL	X	Y		X	Y	
TPOX	8	11		8	11	
CSF1PO	12	12		12	12	
D12S391	19	22				
FGA	22	22				
D2S1338	17	25				
D21S11	29	32.2				
D18S51	11	17				
D8S1179	15	15				
D3S1358	17	17				
D6S1043	11	13				
PENTAE	7	14				
D19S433	14	16				
PENTAD	9.1	14				

Cell Line Authentication and Identification Service 邮箱：18521538068@163.com 服务热线：86+18521538068
联系地址：上海市松江区广富林街龙腾路 1015 弄 2 号楼 518 室

其 他 说 明

(一)　分型方案及位点分布

	方案 1	方案 2	方案 3	方案 4
1	TH01	TPOX	D3S1358	AMEL
2	D12S391	VWA	D13S317	D5S818
3	D7S820	D8S1179	D6S1043	D2S1338
4	CSF1PO	PENTAD	D16S539	D21S11
5	FGA		D19S433	D18S51
6	PENTAE			

实验方案及位点

(二)　STR 数据库比对

本公司采用 DSMZ tools 进行细胞系比对，其中包含来自于 ATCC, DSMZ, JCRB 和 RIKEN 数据库的 2455 个细胞系 STR 数据。如果待检测细胞未收录于以上细胞库或这是自行建立的新细胞系将无法进行比对，用户需根据细胞分型结果自行与其他数据库进行比对。

主要实验人员：张佳男

复核人: 张晨茜

负责人： 王敏

签发日期：2022 年 3 月 7 日

Cell Line Authentication and Identification Service 邮箱：18521538068@163.com　服务热线：86+18521538068
联系地址：上海市松江区广富林街龙腾路 1015 弄 2 号楼 518 室

Sample File	Sample Name	Panel	SQI	OS	SQ
82_B11_CellLineAuthentication-1-1127.FSA	HCT-8	STR Profile 1-human			

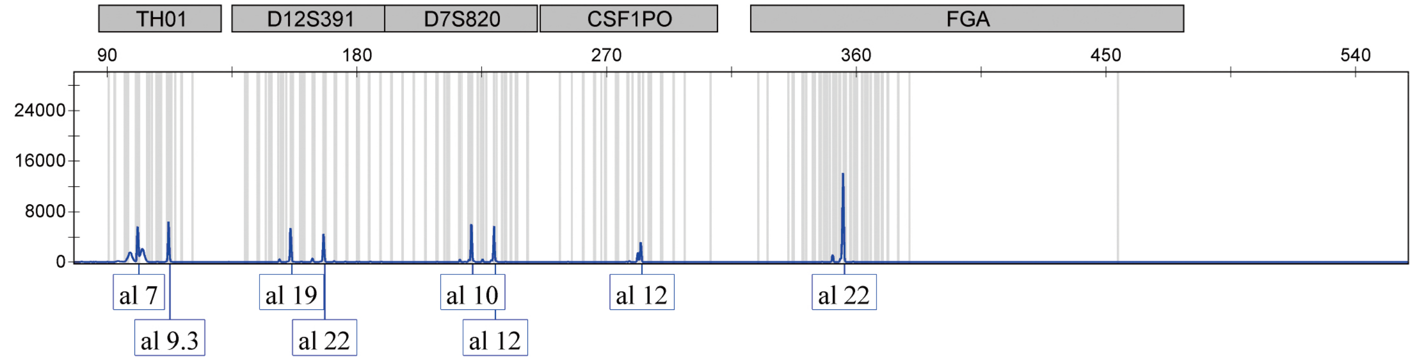

Sample File	Sample Name	Panel	SQI	OS	SQ
82_B11_CellLineAuthentication-1-1127.FSA	HCT-8	STR Profile 1-human			

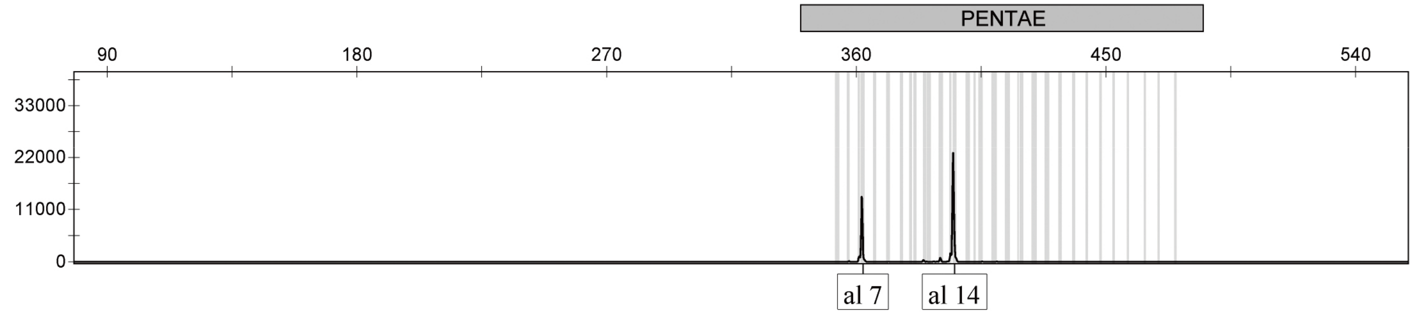

Sample File	Sample Name	Panel	SQI	OS	SQ
10_B02_CellLineAuthentication-2-1127.FSA	HCT-8	STR Profile 2-human			

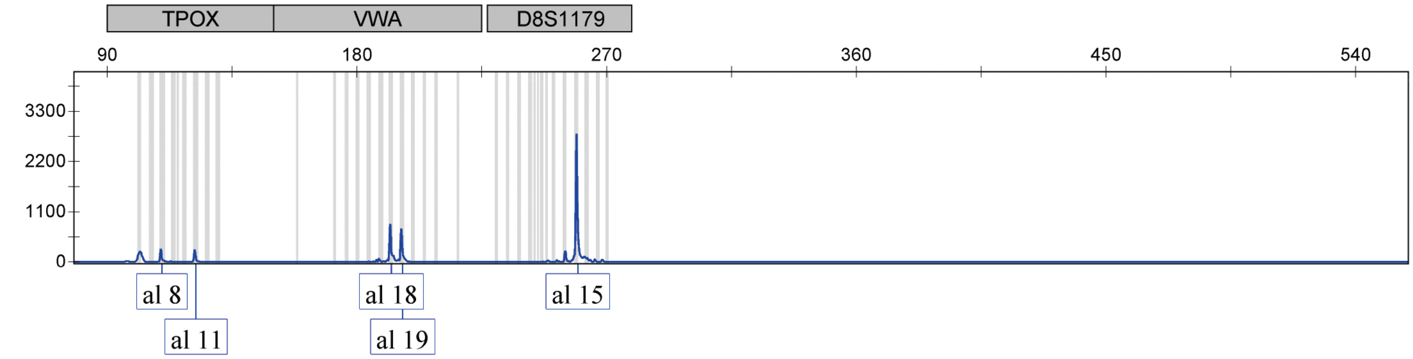

Sample File	Sample Name	Panel	SQI	OS	SQ
10 B02 CellLineAuthentication-2-1127.FSA	HCT-8	STR Profile 2-human			

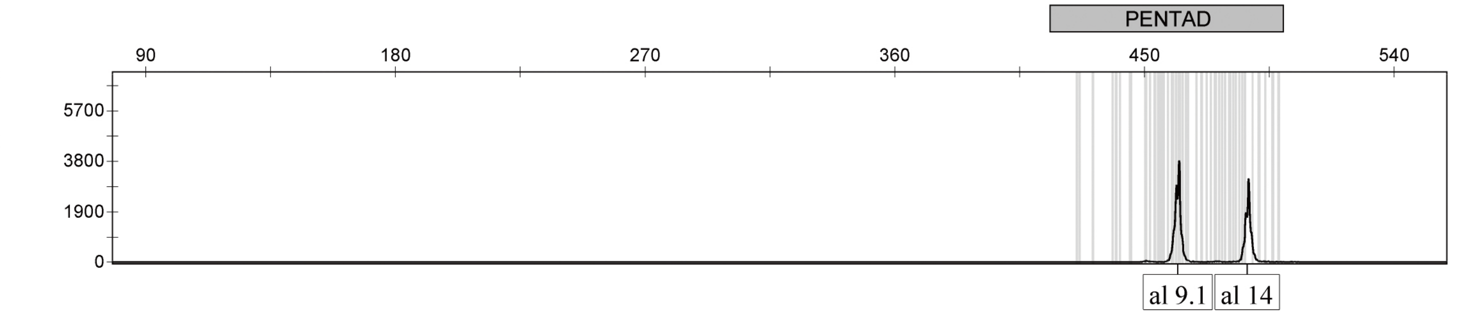

50 B07 CellLineAuthentication-2-1127.FSA	HCT-8	STR Profile 3-human			

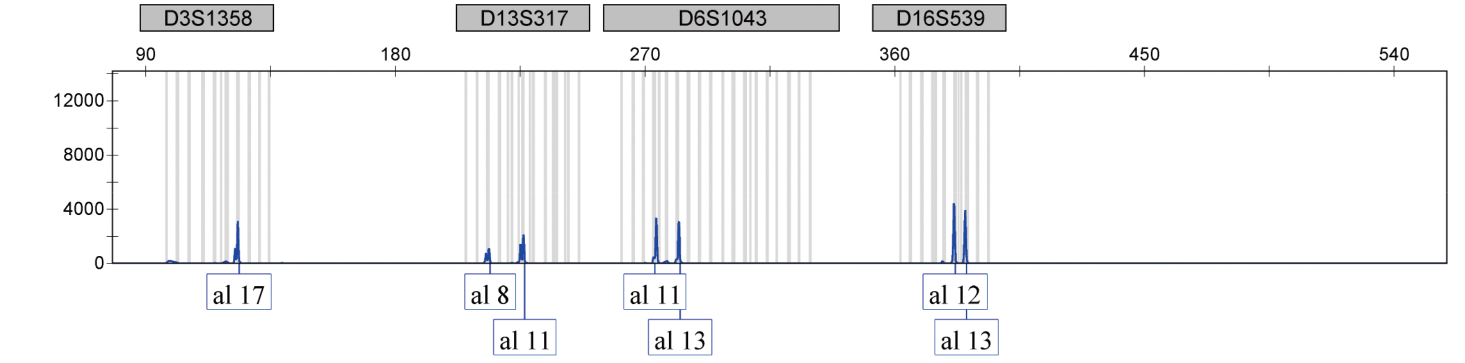

50 B07 CellLineAuthentication-2-1127.FSA	HCT-8	STR Profile 3-human			

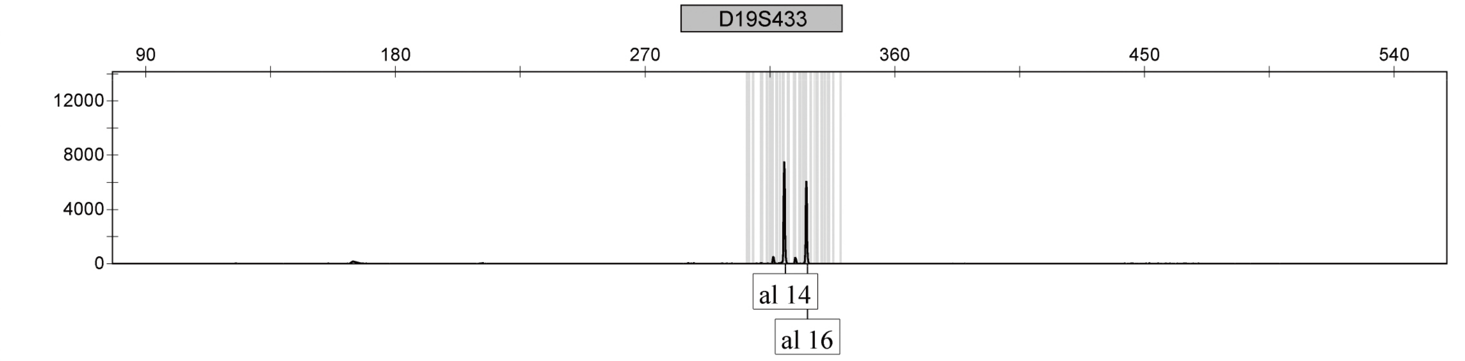

Sample File	Sample Name	Panel	SQI	OS	SQ
10_B02_XiBaoJianDing1122Pi-2(1-5)-1129.FSA	HCT-8	STR Profile 4-human			

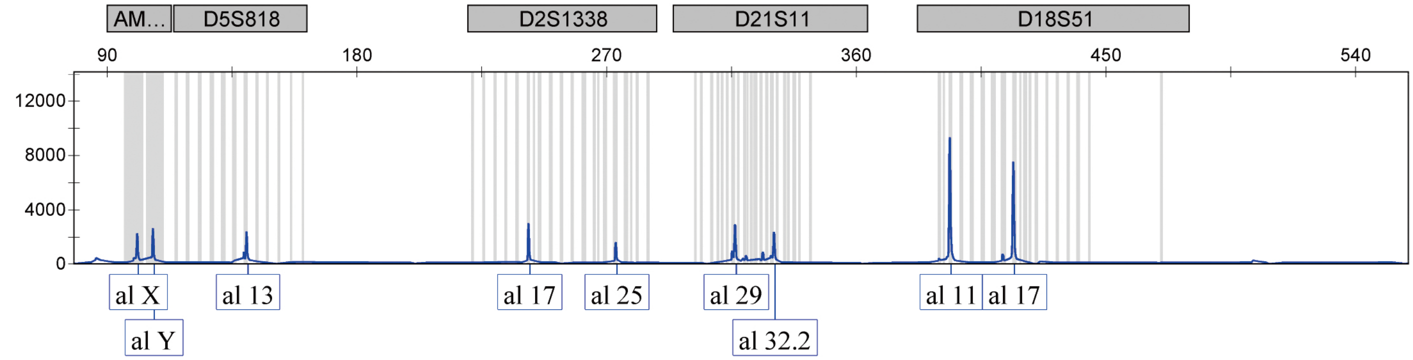

Sample File	Sample Name	Panel	SQI	OS	SQ
10_B02_XiBaoJianDing1122Pi-2(1-5)-1129.FSA	HCT-8	STR Profile 4-human			

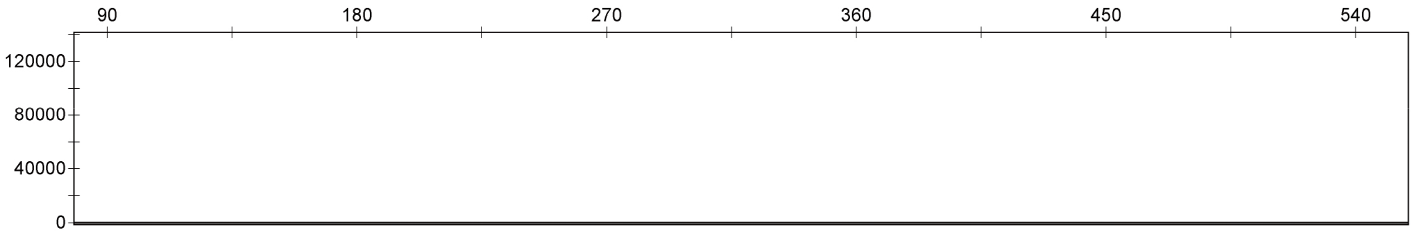

Supplement: Supplementary file 1 [file OncolRes-34-71902-s001.zip › OR_71902-s001/HCT-8-STR.pdf]
