# Supplementary material for: Thimerosal Inhibits Tumor Malignant Progression through Direct Action and Enhancing the Efficacy of PD-1-Based Immunotherapy
Source: Oncol Res. 2026 Jan 19;34(2):20. doi: 10.32604/or.2025.071902 (PMC12848756; doi:10.32604/or.2025.071902)
Supplement: Supplementary file 1 [file OncolRes-34-71902-s001.zip › OR_71902-s001/HCT116-STR.pdf]

Report of Human Cell Line Authentication

Delivery Date: Jul 08th, 2020

Analysis Date: Jul 14th, 2020

I. Sample

Sample Name: 'JD3748', labeled as '116', and was received on Jul 08th, 2020

II. Method and Procedure

1. PCR is amplified with STR Multi-amplification Kit (PowerPlex 21D System);
2. PCR products are assayed with 3730 DNA Analyzer (Applied Biosystems®).
3. Amplification of gene COX1 and electrophoresis are employed to survey the species of the sample.

III. Results

1. The STR profiles of the cell line sample are in the attached table and figure.
2. The search result in ATCC and DSMZ databases.
3. The electrophoresis figure of gene COX1.

116: ①No loci has tri-alleles or tetra-alleles. Contamination of other human cell lines are not found (Figure 1 & Table 1). ②Compared the STR data of cell line in the databases of ATCC and DSMZ, the alleles of 116 were 89% matched with the alleles of HCT 116 cells found in both cell banks. (Figure 2 & Figure 3) ③The sample is a human cell line. Contamination of other species cells are not found in the sample (Figure 4).

To all above, the sample is a single cell line, and it is derived from a common ancestry with HCT 116 cell line.

Operator: Wanting Jiang

Auditor: Xuanyi Liang

GUANGZHOU CELLCOOK BIOTECH CO.,LTD
14 JUL 2020
CELL STR PROFILING
PASS

Guangzhou Cellcook Biotech Co., Ltd

Figure 1. STR profiles of 116 cell line

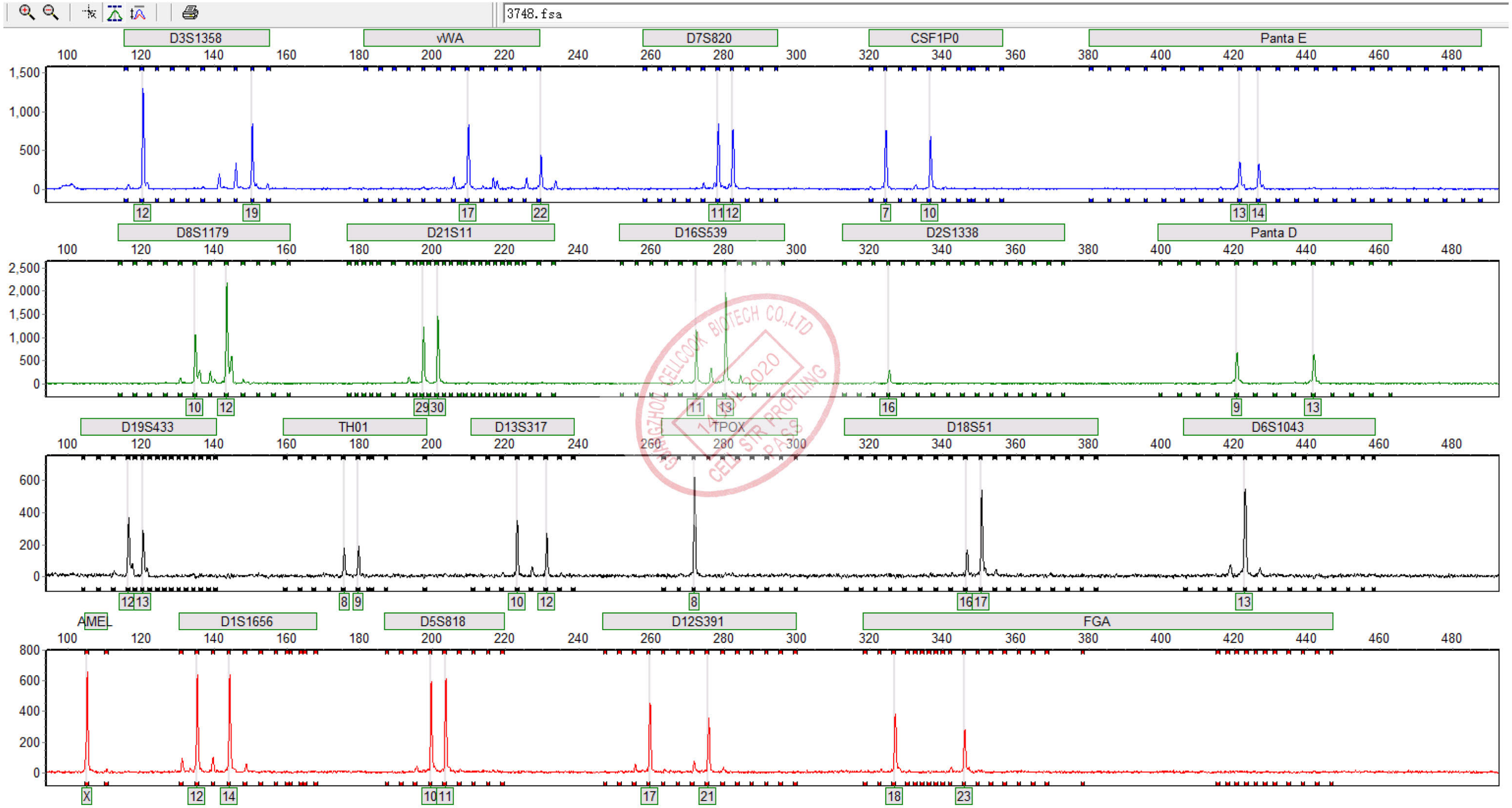

Table 1. STR profiles of 116 cell line

21	Allele1	Allele2
D3S1358	12	19
vWA	17	22
D7S820	11	12
CSF1PO	7	10
Penta E	13	14
D8S1179	10	12
D21S11	29	30
D16S539	11	13
D2S1338	16	
Penta D	9	13
D19S433	12	13
TH01	8	9
D13S317	10	12
TPOX	8	
D18S51	16	17
D6S1043	13	
AMEL	X	
D1S1656	12	14
D5S818	10	11
D12S391	17	21
FGA	18	23

Figure 2. Search result in ATCC database

SEARCH THE STR DATABASE

As part of our continuing efforts to characterize and authenticate the cell lines in the Cell Biology collection, ATCC has developed a comprehensive database of short tandem repeat (STR) DNA profiles for all of our human cell lines. View our brief tutorial before starting.

1. STR Profiling Analysis
2. Matching Algorithm
3. Interrogating the Database

Showing 1 - 2 Of 2

PageSize: 100

Add to Cart	%Match	ATCC® Number	Designation	D5S818	D13S317	D7S820	D16S539	vWA	TH01	AMEL	TPOX	CSF1PO
☐	89.0	CCL-247	HCT 116Colon CarcinomaHuman	10,11	10,12	11,12	11,13	17,22	8,9	X,Y	8,9	7,10
☐	88.0	CRL-2780	ATRFLOXColon CarcinomaHuman	10,12	10,12	11,12	11,13	17,22	8,9	X	8	7,9

Figure 3. Search result in DSMZ database

Result of STR matching analysis by your data.

- DSMZ Profile Database -

A graphical presentation is shown at the bottom of this page.

EV	Cell No.	Cell name	Locus names									Figures
			D5S818	D13S317	D7S820	D16S539	VWA	TH01	AM	TPOX	CSF1PO	
	Query (Your Cell)		*10,11*	*10,12*	*11,12*	*11,13*	*17,22*	*8,9*	*X,X*	*8,8*	*7,10*	
0.89(32/36)	CCL-247	HCT 116	10,11	10,12	11,12	11,13	17,22	8,9	X,Y	8,9	7,10	-
0.89(32/36)	CRL-2780	ATRFLOX [Mutatect]	10,12	10,12	11,12	11,13	17,22	8,9	X,X	8,8	7,9	-
0.84(32/38)	581	HCT-116	10,11	10,12	11,12	11,13,12	17,17,21	8,8	X,X	8,8	7,10	-

Figure 4. Authentication of the species of the sample

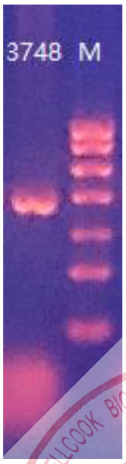

M: Marker. As the size of 700, 600, 500, 400, 300, 200 and 100bp from up to down.

Eight species are checked, as follow: *Homo sapiens* 391bp, *Cricetulus griseus*315bp, *Macaca mulatta*287bp, *Cercopithecus aethiops*222bp, *Rattus norvegicus*196bp, *Canis familiaris*172bp, *Mus musculus*150bp, *Bos Taurus*102bp

JD3748: The sample. The band size is 391bp which matches the size of human.
