# Supplementary material for: Thimerosal Inhibits Tumor Malignant Progression through Direct Action and Enhancing the Efficacy of PD-1-Based Immunotherapy
Source: Oncol Res. 2026 Jan 19;34(2):20. doi: 10.32604/or.2025.071902 (PMC12848756; doi:10.32604/or.2025.071902)
Supplement: Supplementary file 1 [file OncolRes-34-71902-s001.zip › OR_71902-s001/LOVO-STR.pdf]

Cell Line Authentication Service

STR Profiling Report

Sample From: Southern Medical University

Sample Type: Cell Line

Testing Method: STR Genotyping

Report Time: January 16, 2025

COMPANY STATEMENT

1. THIS REPORT IS ONLY RESPONSIBLE FOR THE SAMPLES ANALYZED.
2. THE TESTING RESULTS AND THE ORGANIZATION NAME WILL NOT BE USED FOR ADVERTISEMENT, COMMERCIAL EXHIBITIONS, COMMERCIAL PERFORMANCE AND OTHER COMMERCIAL ACTIVITIES.
3. OBJECTIONS SHOULD BE RAISED WITHIN FIFTEEN DAYS AFTER THE RECEIPT OF THIS REPORT.
4. THE PAPER REPORT WITH CONTENT ALTERING, ADDING OR WITHOUT THE STAMPED SEAL OF THE COMPANY ARE INVALID.

Testing Company: Shanghai Biowing Applied Biotechnology Co. Ltd

Address: Room 502, NO.1015 Longteng Rd, Songjiang District, Shanghai

Tel: +86-18521538068

Contact: Shuangning Zhu

E-mail: zhusn@biowing.com.cn

Cell Line Authentication – STR Profiling Report

Sample code

Table 1. Sample Code

Customer's code	Company Code
LOVO	20250113-02

Sample Number :1

Sample Type: Cell line

Testing Type: STR

Testing Method:

DNA was extracted by a commercial kit from CORNING (AP-EMN-BL-GDNA-250G). The twentyone STRs including Amelogenin locus were amplified by six multiplex PCR and separated on ABI 3730XL Genetic Analyzer. The signals were then analyzed by the software GeneMapper.

Data Interpretation:

Cell lines were authenticated using Short Tandem Repeat (STR) analysis asdescribed in 2021 in ANSI Standard (ASN-0002) by the ATCC Standards Development Organization (SDO) and in Capes-Davis et al., Match criteria for human cell line authentication: Where do we draw the line? Int J Cancer.2013;132(11):2510-9.

Test Results

1. STR profile

Table 2. STR and Amelogenin Genotyping Results of Cell line.

Loci	Sample information			Cell Bank information		
	Sample name: LOVO			Cell line name: LoVo		
	**Allele1**	**Allele2**	**Allele3**	**Allele1**	**Allele2**	**Allele3**
D5S818	11	13		11	13	
D13S317	8	11		8	11	
D7S820	9.3	10	11	9.3	10	11
D16S539	9	12		9	12	
VWA	17	18		17	18	
TH01	9.3	9.3		9.3	9.3	
AMEL	X	Y		X	Y	
TPOX	8	9		8	9	
CSF1PO	10	11	13 14	10	11	13 14
D12S391	20	22	23			
FGA	18	20				
D2S1338	17	18				
D21S11	29	31.2				
D18S51	13	17	18			
D8S1179	10	10				
D3S1358	14	17	18			
D6S1043	11	17				
PENTAE	10	16				
D19S433	14	15				
PENTAD	10	14				
D1S1656	13	14	16.3			

2. database annotation

Figure 1. STR matching analysis

EV	Cell No.	Cell name	Locus names								
			D5S818	D13S317	D7S820	D16S539	VWA	TH01	AM	TPOX	CSF1PO
	Query (Your Cell)		11,13,,	8,11,,	9.3,10,11,	9,12,,	17,18,,	9.3,9.3,,	X,Y,,	8,9,,	10,11,13,14
1.0(36/36)	CVCL_0399	LoVo	['11', '13']	['8', '11']	['9.3', '10', '11']	['9', '12']	['17', '18']	['9.3', '9.3']	['X', 'Y']	['8', '9']	['10', '11', '13', '14']

Note: The STR online match analysis of the test cell against DSMZ/ATCC/EXPASY database, showing cell number (Cell No.) and cell name.

3. Explanation of Test Results

☐ The submitted sample profile is human, but not a match for any profile in the DSMZ STR database.

☑ The submitted profile is exact match for the following human cell line(s) in the DSMZ STR database (8 core loci plus Amelogenin): **LoVo**.

☐ The submitted profile is similar to the following DSMZ human cell line: /.

- **Note:** Cell lines are considered to related, derived from a common ancestry, when ≥80% of the alleles in its STR profile match profiles from tissue or other cell line samples from that donor or from database. Cell lines with between a 55% to 80% (similar) match require further profiling for investigation of relatedness.

Figure 2. STR profiles of sample cell line

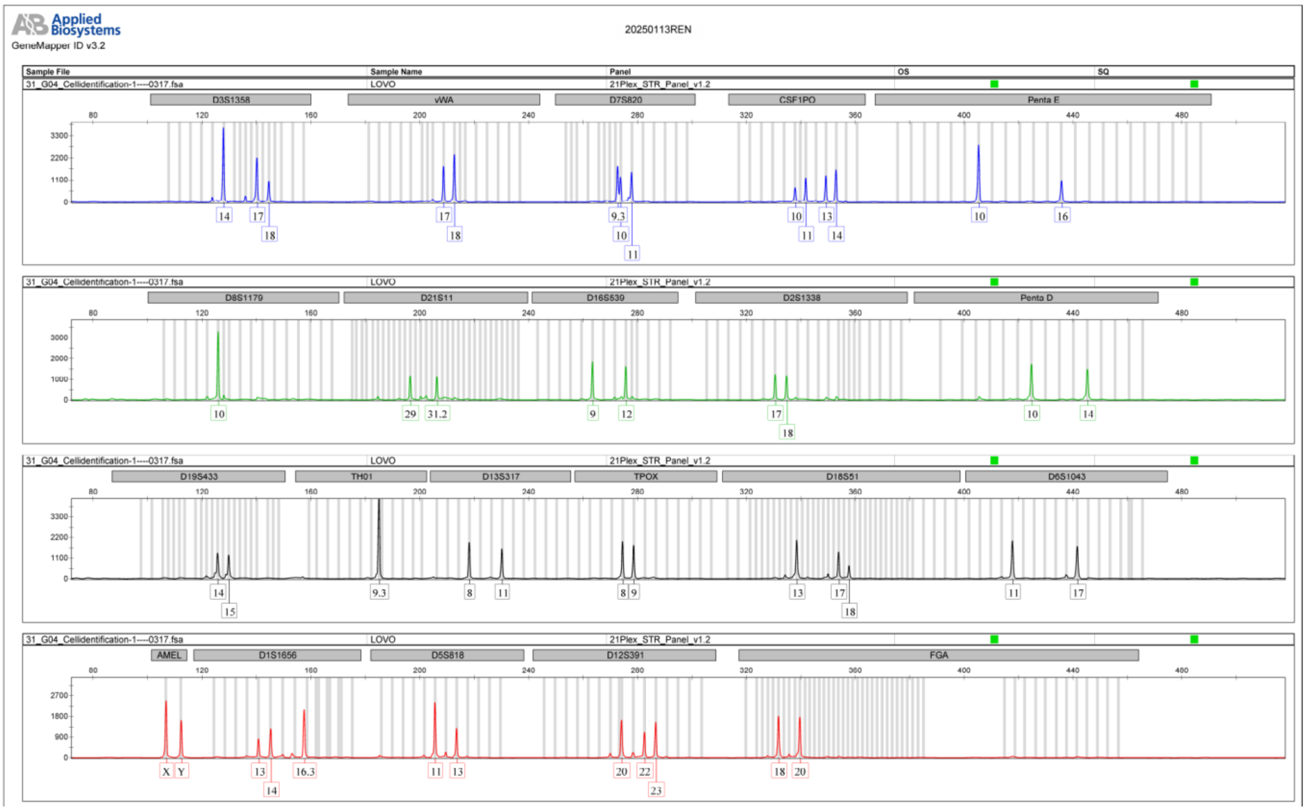

Appendix

1. Genotyping Strategy and Site Distribution

Table S1. Experimental Strategy and Sites

	Strategy 1	Strategy 2	Strategy 3	Strategy 4
1	D3S1358	D8S1179	D19S433	AMEL
2	VWA	D21S11	TH01	D1S1656
3	D7S820	D16S539	D13S317	D5S818
4	CSF1PO	D2S1338	TPOX	D12S391
5	PENTAE	PENTAD	D18S51	FGA
6			D6S1043	

The allele match algorithm compares the 8 core loci plus amelogenin only, even though alleles from all lociwill be reported when available.

2. STR tools was used to carry on the cell line comparison, which contains more than 8000 cell lines STR data from ATCC, DSMZ, JCRB, ECACC, GNE and RIKEN databases. If the cell is not included in the above cell library, users need to compared with other databases.

Technician: Xiuchuan He

Checked by: Chenqian Zhang

Issued by: Min Wang

Issue date: January 16, 2025
