# Supplementary material for: Thimerosal Inhibits Tumor Malignant Progression through Direct Action and Enhancing the Efficacy of PD-1-Based Immunotherapy
Source: Oncol Res. 2026 Jan 19;34(2):20. doi: 10.32604/or.2025.071902 (PMC12848756; doi:10.32604/or.2025.071902)
Supplement: Supplementary file 1 [file OncolRes-34-71902-s001.zip › OR_71902-s001/LS_174T-STR.pdf]

Report of Human Cell Line Authentication

Ⅰ. Sample

Sample Name: labeled as ‘LS 174T’

Ⅱ. Method and Procedure

1. PCR is amplified with STR Multi-amplification Kit (PowerPlex™16HS System);
2. PCR products are assayed with 3100 DNA Analyzer (Applied Biosystems®).
3. Amplification of gene COX1 and electrophoresis are employed to survey the species of the sample.

Ⅲ. Results

1. The STR profiles of the cell line sample are in the attached table and figure.
2. The search result in ATCC and DSMZ databases.

The electrophoresis figure of gene COX1.

LS 174T: ①Five loci have tri-alleles (D3S1358, D21S11, vWA, D8S1179 and FGA). Contamination of other human cell lines are not found (Figure 1 & Table 1). ② Compared the STR data of LS 174T cell line in the databases of ATCC and DSMZ, all the alleles of LS 174T were 84% matched with the alleles of LS 174T cells found in ATCC cell bank (Figure 2&3). ③The sample is a human cell line. Contamination of other species cells are not found in the sample (Figure 4).

To all above, the sample is a single cell line, and it is derived from a common ancestry with LS 174T cell line.

Operator: Xiaohua Mo　　　　　Auditor: Xuanyi Liang

Guangzhou Cellcook Biotech Co., Ltd

GUANGZHOU CELLCOOK BIOTECH CO.,LTD 19 MAY 2020 CELL STR PROFILING

（Notice:This authentication report is restricted to the cell sold from Guangzhou Cellcook Biotech Co., Ltd, and the date with seal is the date of delivery. ）

Figure 1. STR profiles of LS 174T cell line

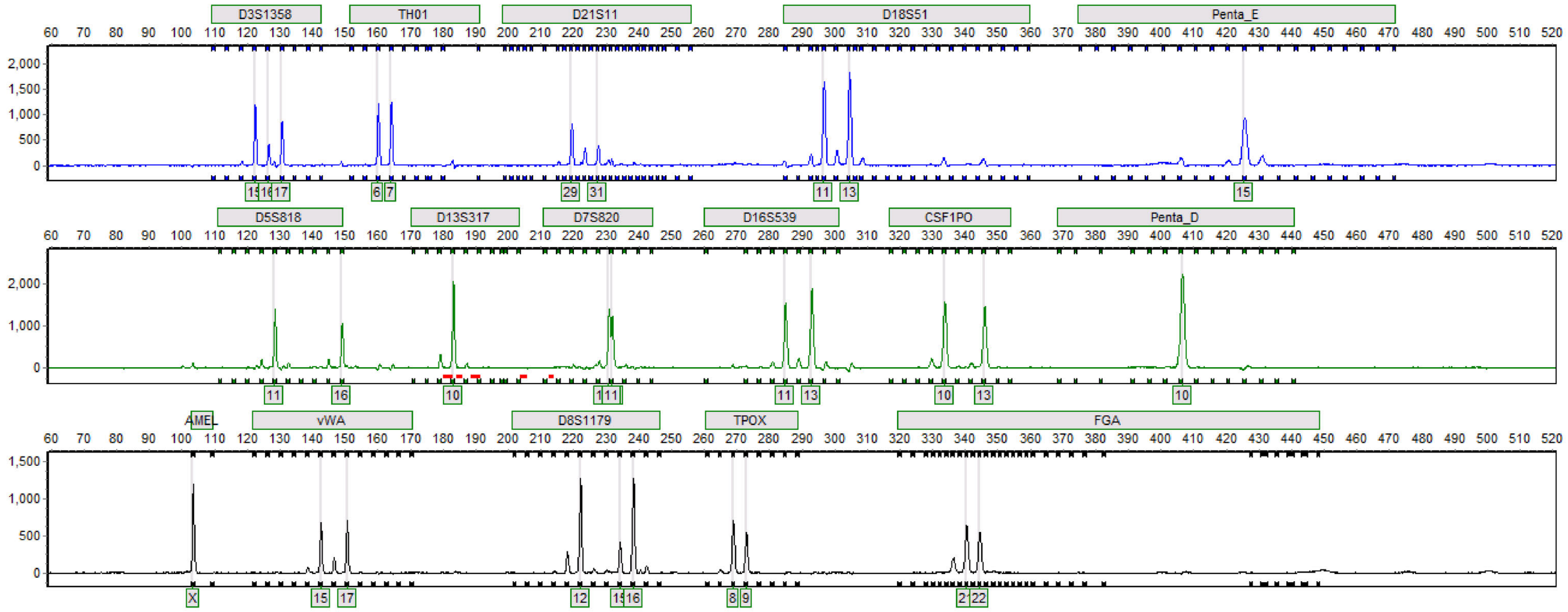

Table 1. STR profiles of LS 174T cell line

	Allele1	Allele2	Allele3
D3S1358	15	16	17
TH01	6	7	
D21S11	29	31	
D18S51	11	13	
Penta_E	15		
D5S818	11	16	
D13S317	10		
D7S820	10.3	11	
D16S539	11	13	
CSF1PO	10	13	
Penta_D	10		
AMEL	x		
vWA	15	17	
D8S1179	12	15	16
TPOX	8	9	
FGA	21	22	

Figure 2. Search result in ATCC database

SEARCH THE STR DATABASE

As part of our continuing efforts to characterize and authenticate the cell lines in the Cell Biology collection, ATCC has developed a comprehensive database of short tandem repeat (STR) DNA profiles for all of our human cell lines. View our brief tutorial before starting.

1. STR Profiling Analysis
2. Matching Algorithm
3. Interrogating the Database

Showing 1 - 1 Of 1

PageSize: 100 ▼

Add to Cart	%Match	ATCC® Number	Designation	D5S818	D13S317	D7S820	D16S539	vWA	TH01	AMEL	TPOX	CSF1PO
☐	84.0	CL-188	LS 174TColon AdenocarcinomaHuman	11,15,16	10	10.3,11	11,13	15,17,18	6,7	X	8,9	10,13,14

Figure 3. Search result in DSMZ database

Result of STR matching analysis by your data.

- DSMZ Profile Database -

A graphical presentation is shown at the bottom of this page.

EV	Cell No.	Cell name	Locus names									Figures
			D5S818	D13S317	D7S820	D16S539	VWA	TH01	AM	TPOX	CSF1PO	
	Query (Your Cell)		*11,16*	*10,10*	*10.3,11*	*11,13*	*15,17*	*6,7*	*x,x*	*8,9*	*10,13*	
0.78(28/36)	CL-187	LS 180	10,15	10,10	11,9.3	11,13	15,18	6,7	X,X	8,9	10,13	-
0.74(28/38)	403	MT-3	12,17	10,11	11,12,10.3	11,13	14,17,13	6,7	X,X	8,9	10,13	-
0.68(28/41)	759	LS-174T	11,15,14	10,11,9	10.3,11	11,12,13	15,18,16	6,7	X,X	8,9	10,14,11	-
0.61(22/36)	197	L-428	11,12	14,14	11,11	11,12	15,15	7,9.3				

Figure 4. Authentication of the species of the sample

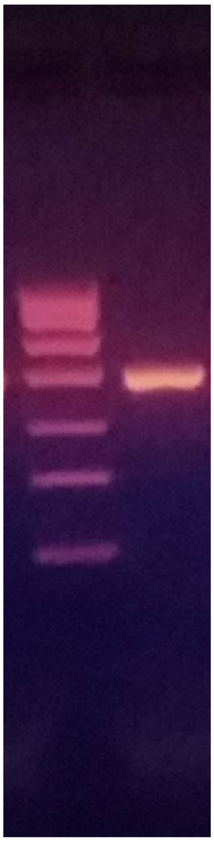

M: Marker. As the size of 700, 600, 500, 400, 300, 200 and 100bp from up to down.

Nine species are checked, as follow: *Homo sapiens* 391bp, *Cricetulus griseus*315bp, *Macaca mulatta*287bp, *Cercopithecus aethiops*222bp, *Rattus norvegicus*196bp, *Canis familiaris*172bp, *Mus musculus*150bp, *Bos Taurus*102bp, IC 70bp

The sample: The band size is 391bp which matches the size of human.
