# Supplementary material for: Thimerosal Inhibits Tumor Malignant Progression through Direct Action and Enhancing the Efficacy of PD-1-Based Immunotherapy
Source: Oncol Res. 2026 Jan 19;34(2):20. doi: 10.32604/or.2025.071902 (PMC12848756; doi:10.32604/or.2025.071902)
Supplement: Supplementary file 1 [file OncolRes-34-71902-s001.zip › OR_71902-s001/NCM460-STR.pdf]

# Cell Line Authentication Service

## STR Profiling Report

**Sample Code:** NCM460

**Sample Type:** Cell Line

**Testing Method:** STR Genotyping

**Report Time:** October 13, 2023


Add: Room 205,.885 Wangjiashe Rd,Songjiang Dist,Shanghai/ Tel:+86-18521538068
Homepage: http://www.biowing.com.cn/ E-mail: zhusn@biowing.com.cn

# COMPANY STATEMENT

1. THIS REPORT IS ONLY RESPONSIBLE FOR THE SAMPLES ANALYZED.
2. THE TESTING RESULTS AND THE ORGANIZATION NAME WILL NOT BE USED FOR ADVERTISEMENT, COMMERCIAL EXHIBITIONS, COMMERCIAL PERFORMANCE AND OTHER COMMERCIAL ACTIVITIES.
3. OBJECTIONS SHOULD BE RAISED WITHIN FIFTEEN DAYS AFTER THE RECEIPT OF THIS REPORT.
4. THE PAPER REPORT WITH CONTENT ALTERING, ADDING OR WITHOUT THE STAMPED SEAL OF THE COMPANY ARE INVALID.

**Testing Company:** Shanghai Biowing Applied Biotechnology Co. Ltd

**Address:** Room 205, NO.885 Wangjiashe Road, Songjiang District, Shanghai

**Tel:** +86-18521538068

**Contact:** Shuangning Zhu

**E-mail:** zhusn@biowing.com.cn

# Cell Line Authentication – STR Profiling Report

## Sample code

Table 1. Sample Code

| Customer's code | Company Code |
|---|---|
| NCM460 | 20231010-01 |

**Sample Number** :1

**Sample Type**: Cell line

**Testing Type**: STR

**Testing Method**:

DNA was extracted by a commercial kit from CORNING (AP-EMN-BL-GDNA-250G). The twenty STRs including Amelogenin locus were amplified by six multiplex PCR and separated on ABI 3730XL Genetic Analyzer. The signals were then analyzed by the software GeneMapper.

**Data Interpretation:**

Cell lines were authenticated using Short Tandem Repeat (STR) analysis asdescribed in 2012 in ANSI Standard (ASN-0002) by the ATCC Standards Development Organization (SDO) and in Capes-Davis et al., Match criteria for human cell line authentication: Where do we draw the line? Int J Cancer.2013;132(11):2510-9.



**Add:** Room 205,.885 Wangjiashe Rd,Songjiang Dist,Shanghai/ Tel:+86-18521538068
Homepage: http://www.biowing.com.cn/ E-mail: zhusn@biowing.com.cn

# Test Results

## 1. STR profile

Table 2. STR and Amelogenin Genotyping Results of Cell line.

| Loci | **Sample information** | | | **Cell Bank information** | | |
|---|---|---|---|---|---|---|
| | Sample name: NCM460 | | | Control name: NCM460 | | |
| | **Allele1** | **Allele2** | **Allele3** | **Allele1** | **Allele2** | **Allele3** |
| D5S818 | 10 | 10 | | 10 | 10 | |
| D13S317 | 9 | 11 | | 9 | 11 | |
| D7S820 | 11 | 12 | | 11 | 12 | |
| D16S539 | 9 | 11 | | 9 | 11 | |
| VWA | 14 | 17 | | 14 | 17 | |
| TH01 | 6 | 7 | | 6 | 7 | |
| AMEL | X | Y | | X | Y | |
| TPOX | 8 | 11 | | 8 | 11 | |
| CSF1PO | 9 | 12 | | 9 | 12 | |
| D12S391 | 21 | 22 | | 21 | 22 | |
| FGA | 19 | 24 | | 19 | 24 | |
| D2S1338 | 17 | 23 | | 17 | 23 | |
| D21S11 | 30 | 32.2 | | 30 | 30 | |
| D18S51 | 13 | 13 | | 13 | 13 | |
| D8S1179 | 13 | 16 | | 13 | 16 | |
| D3S1358 | 16 | 16 | | 16 | 16 | |
| D6S1043 | 18 | 19 | | 18 | 19 | |
| PENTAE | 18 | 18 | | 18 | 20 | |
| D19S433 | 14 | 15.2 | | 14 | 15.2 | |
| PENTAD | 11 | 12 | | 11 | 12 | |
| D1S1656 | 14 | 16 | | 14 | 16 | |


**Add:** Room 205,.885 Wangjiashe Rd,Songjiang Dist,Shanghai/ Tel:+86-18521538068
Homepage: http://www.biowing.com.cn/ E-mail: zhusn@biowing.com.cn

## 2. database annotation

Figure 1. STR matching analysis

| EV | Cell No. | Cell name | Locus names | | | | | | | | | Figures |
|---|---|---|---|---|---|---|---|---|---|---|---|---|
| | | | D5S818 | D13S317 | D7S820 | D16S539 | VWA | TH01 | AM | TPOX | CSF1PO | |
| | *Query (Your Cell)* | | *10,10* | *9,11* | *11,12* | *9,11* | *14,17* | *6,7* | *X,Y* | *8,10,11* | *9,12* | |
| 0.70(26/37) | JCRB1197 | PL509 | 9,10 | 9,11 | 11,12 | 11,12 | 14,17 | 6,6 | X,Y | 8,8 | 10,12 | - |
| 0.70(26/37) | RCB0222 | NB1RGB | 7,10 | 9,11 | 12,13 | 9,11 | 14,18 | 6,9 | X,Y | 8,11 | 10,12 | - |

**Note:** The STR online match analysis of the test cell against DSMZ/ATCC/EXPASY database, showing cell number (Cell No.) and cell name.

## 3. Authentication

☑ The submitted sample profile is human, but not a match for any profile in the DSMZ. As the STR database of **NCM460** was not logged in, the matching result cannot be displayed. The sample that perfectly matches the control cell line **NCM460,** is unique and not cross-contaminated or misidentified, if the paper was to publish, these data could be submitted to the magazine.

☐ The submitted profile is an exact match for the following human cell line(s) in the DSMZ STR database (8 core loci plus Amelogenin): /.

☐ The submitted profile is similar to the following DSMZ human cell line: /.

- **Note:** Cell lines are considered to related, derived from a common ancestry, when ≥80% of the alleles in its STR profile match profiles from tissue or other cell line samples from that donor or from database. Cell lines with between a 55% to 80% (similar) match require further profiling for investigation of relatedness.



**Add:** Room 205,.885 Wangjiashe Rd,Songjiang Dist,Shanghai/ Tel:+86-18521538068
Homepage: http://www.biowing.com.cn/ E-mail: zhusn@biowing.com.cn

Figure 2. STR profiles of sample cell line (sample)

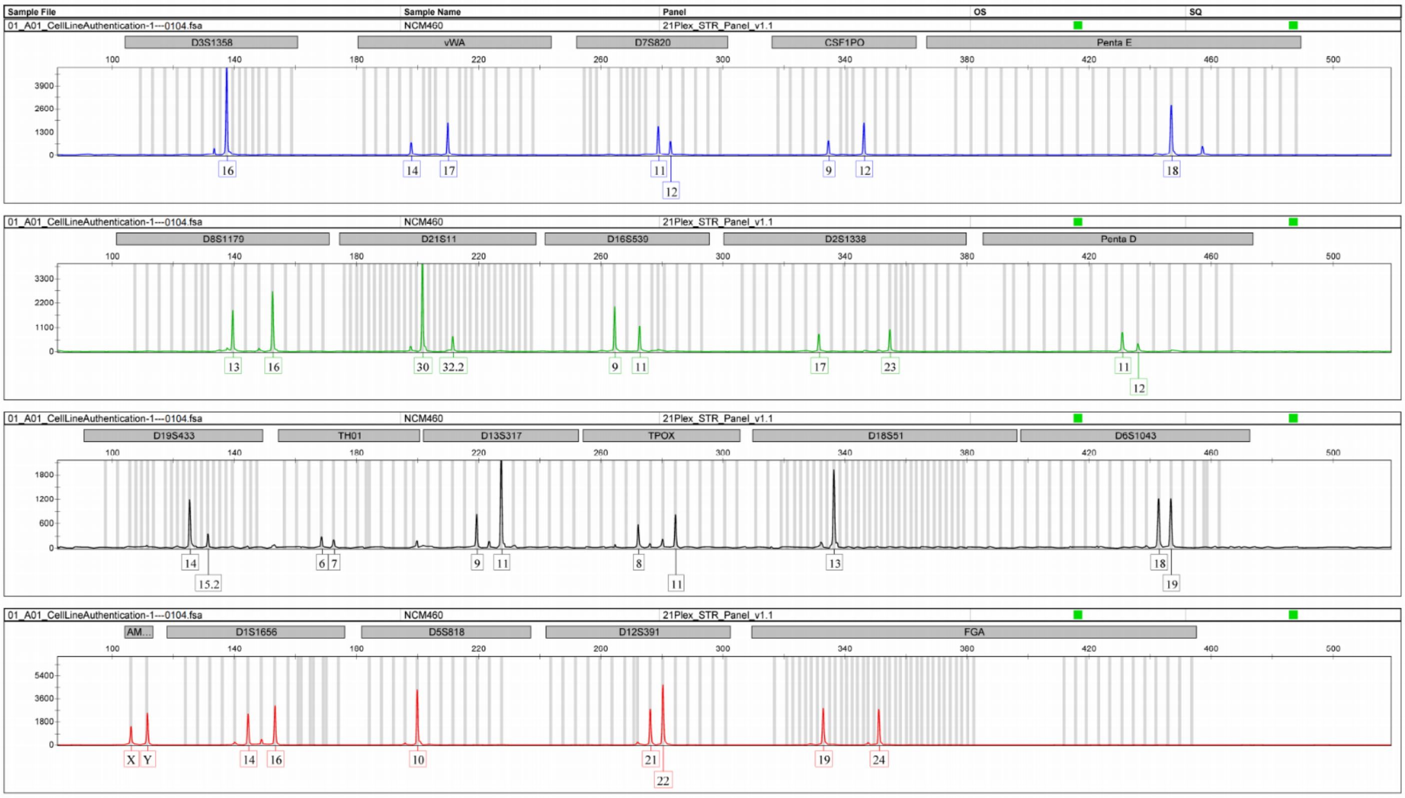


Figure 3. STR profiles of control cell line (NCM460)

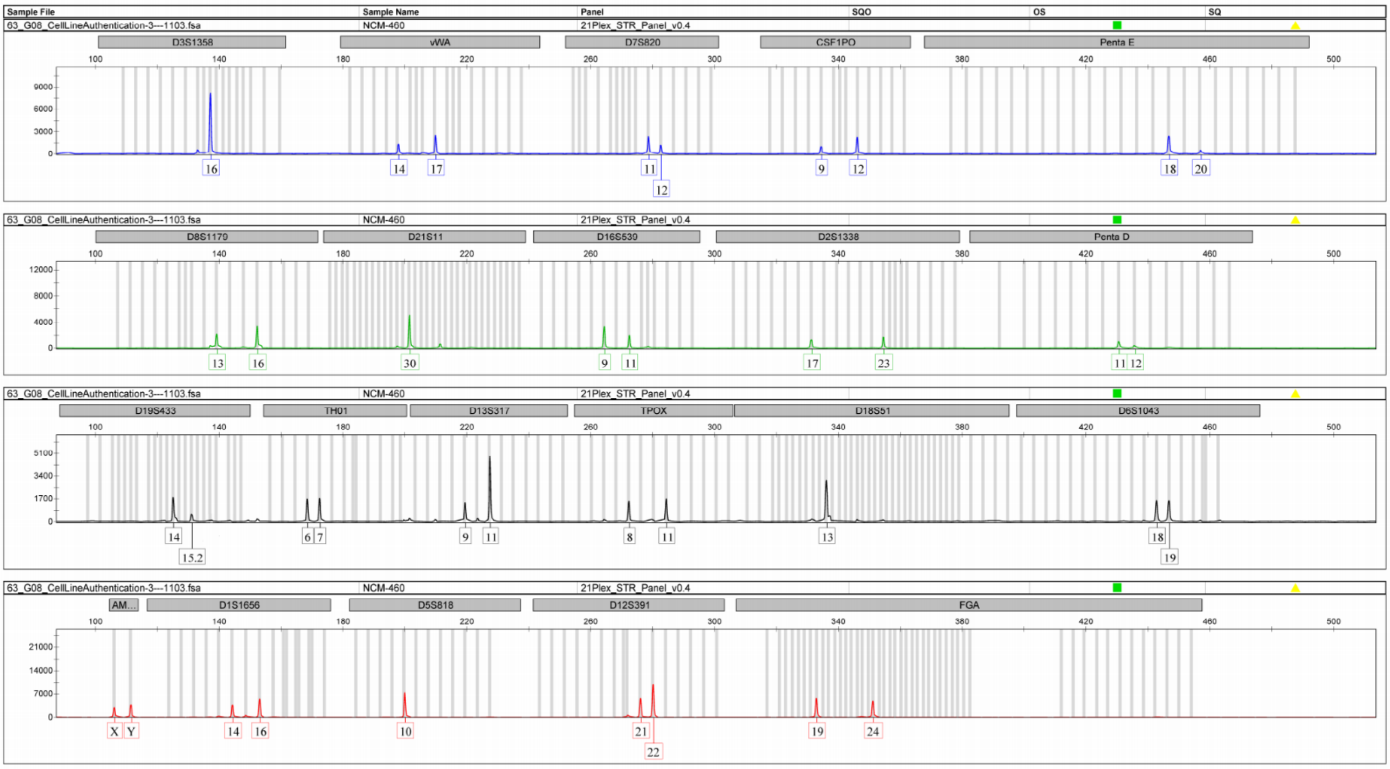




**Add:** Room 205,.885 Wangjiashe Rd,Songjiang Dist,Shanghai/ Tel:+86-18521538068
Homepage: http://www.biowing.com.cn/ E-mail: zhusn@biowing.com.cn

# Appendix

1. Genotyping Strategy and Site Distribution

Table S1. Experimental Strategy and Sites

| | Strategy 1 | Strategy 2 | Strategy 3 | Strategy 4 |
|---|---|---|---|---|
| 1 | D3S1358 | D8S1179 | D19S433 | AMEL |
| 2 | VWA | D21S11 | TH01 | D1S1656 |
| 3 | D7S820 | D16S539 | D13S317 | D5S818 |
| 4 | CSF1PO | D2S1338 | TPOX | D12S391 |
| 5 | PENTAE | PENTAD | D18S51 | FGA |
| 6 | | | D6S1043 | |

*The allele match algorithm compares the 8 core loci plus amelogenin only, even though alleles from all lociwill be reported when available.*

2. DSMZ tools was used to carry on the cell line comparison, which contains 2455 cell lines STR data from ATCC, DSMZ, JCRB ,ECACC，GNE and RIKEN databases. If the cell is not included in the above cell library, users need to compared with other databases.

**Technician:** Jianan Zhang

**Checked by:** Chenqian Zhang

**Issued by**: Wang Min

**Issue date:** October 13, 2023


**Add:** Room 205,.885 Wangjiashe Rd,Songjiang Dist,Shanghai/ Tel:+86-18521538068
Homepage: http://www.biowing.com.cn/ E-mail: zhusn@biowing.com.cn
