# Supplementary material for: Thimerosal Inhibits Tumor Malignant Progression through Direct Action and Enhancing the Efficacy of PD-1-Based Immunotherapy
Source: Oncol Res. 2026 Jan 19;34(2):20. doi: 10.32604/or.2025.071902 (PMC12848756; doi:10.32604/or.2025.071902)
Supplement: Supplementary file 2 [file OncolRes-34-71902-s002.docx]

**Figure 1G**

Caspase3 32kDa

Cleaved Caspase3 17kDa

PARP 116kDa

Cleaved PARP 89kDa


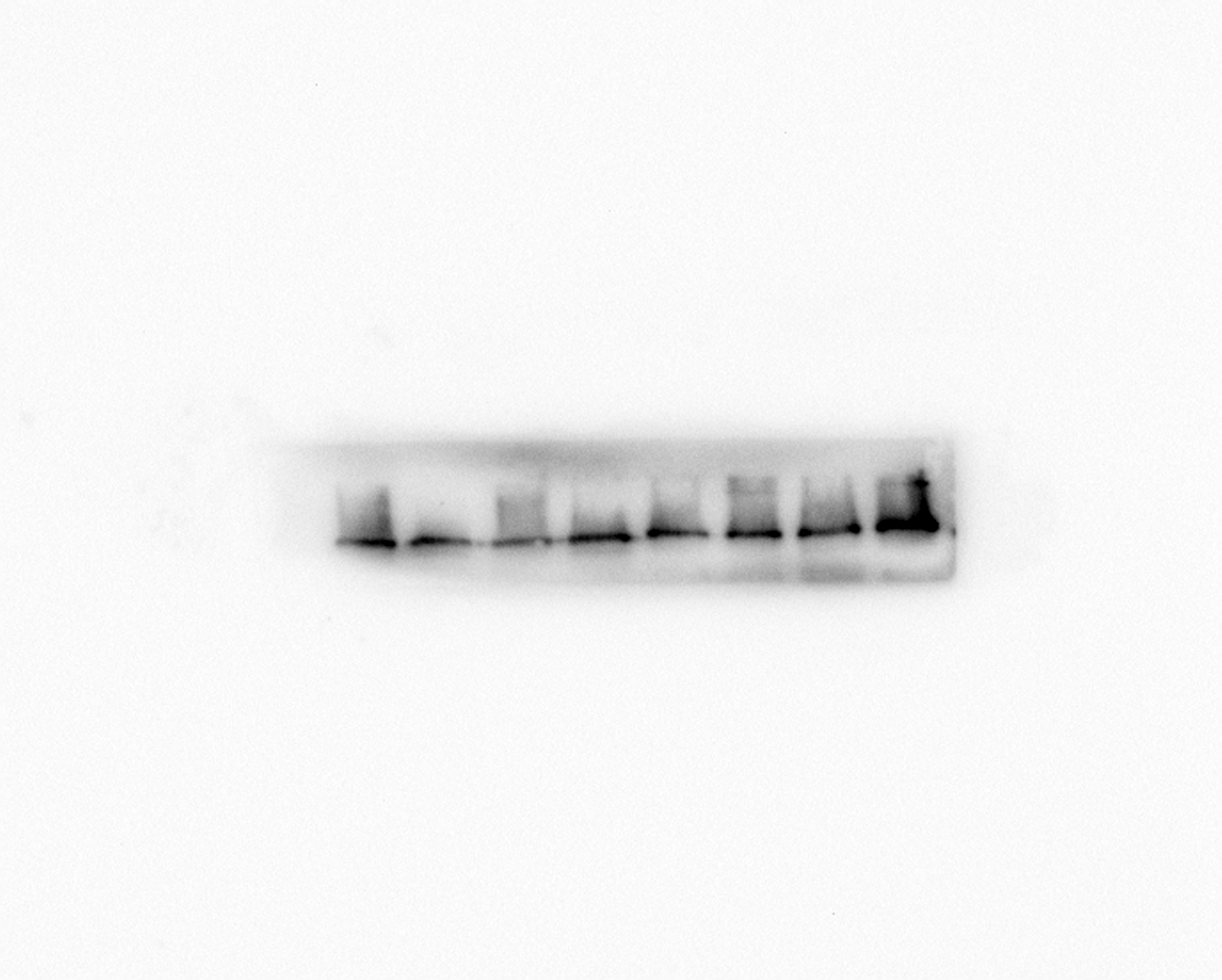

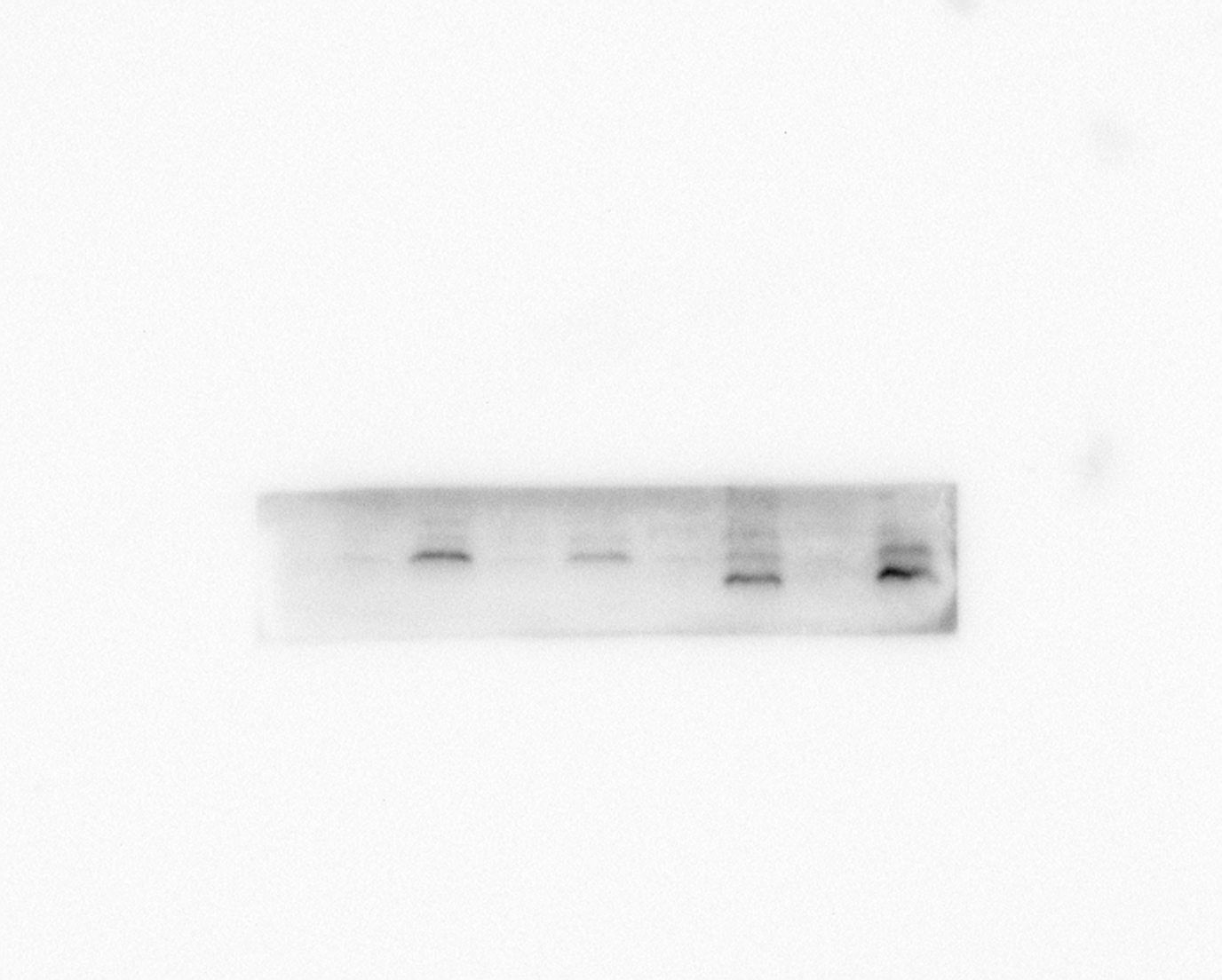

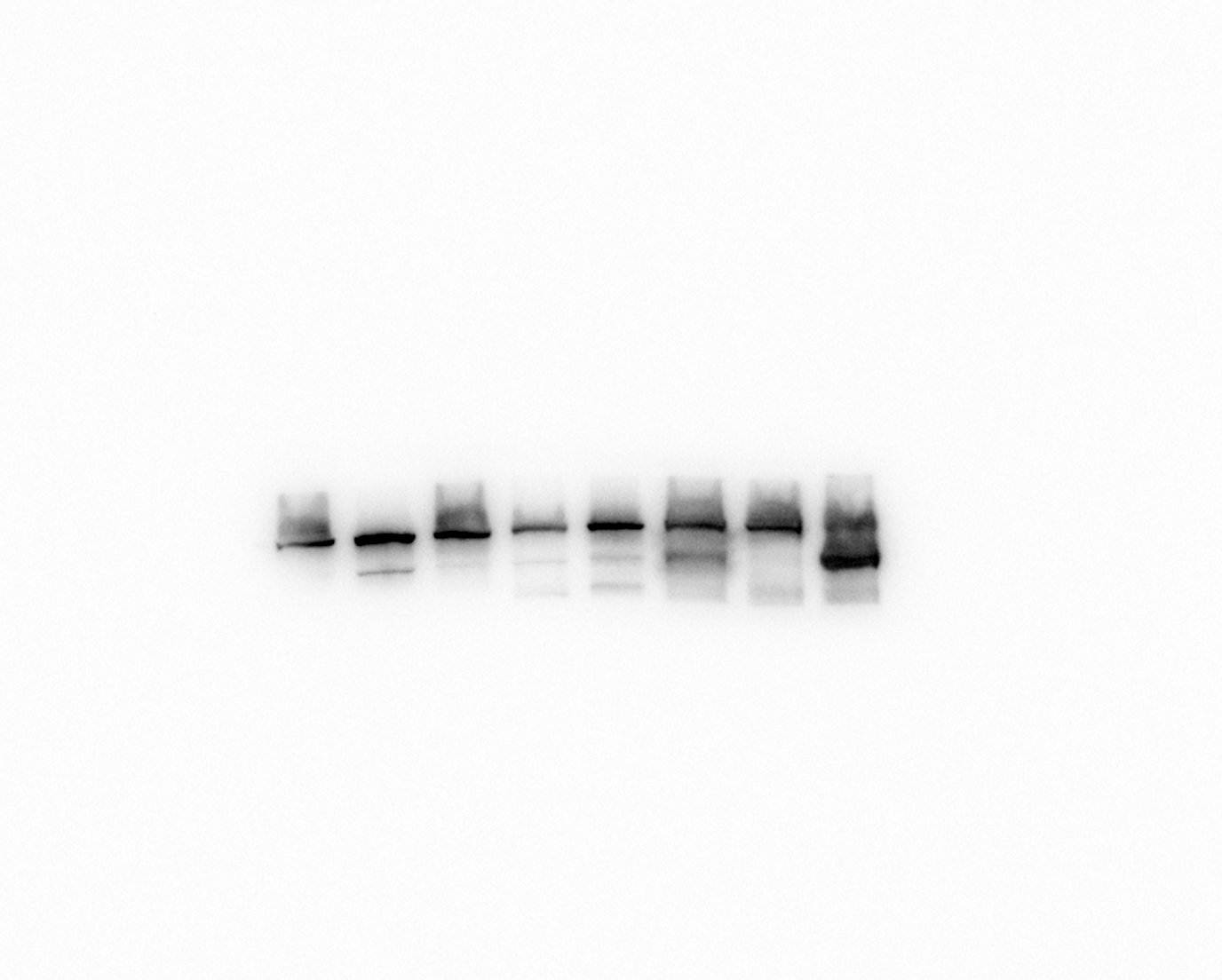

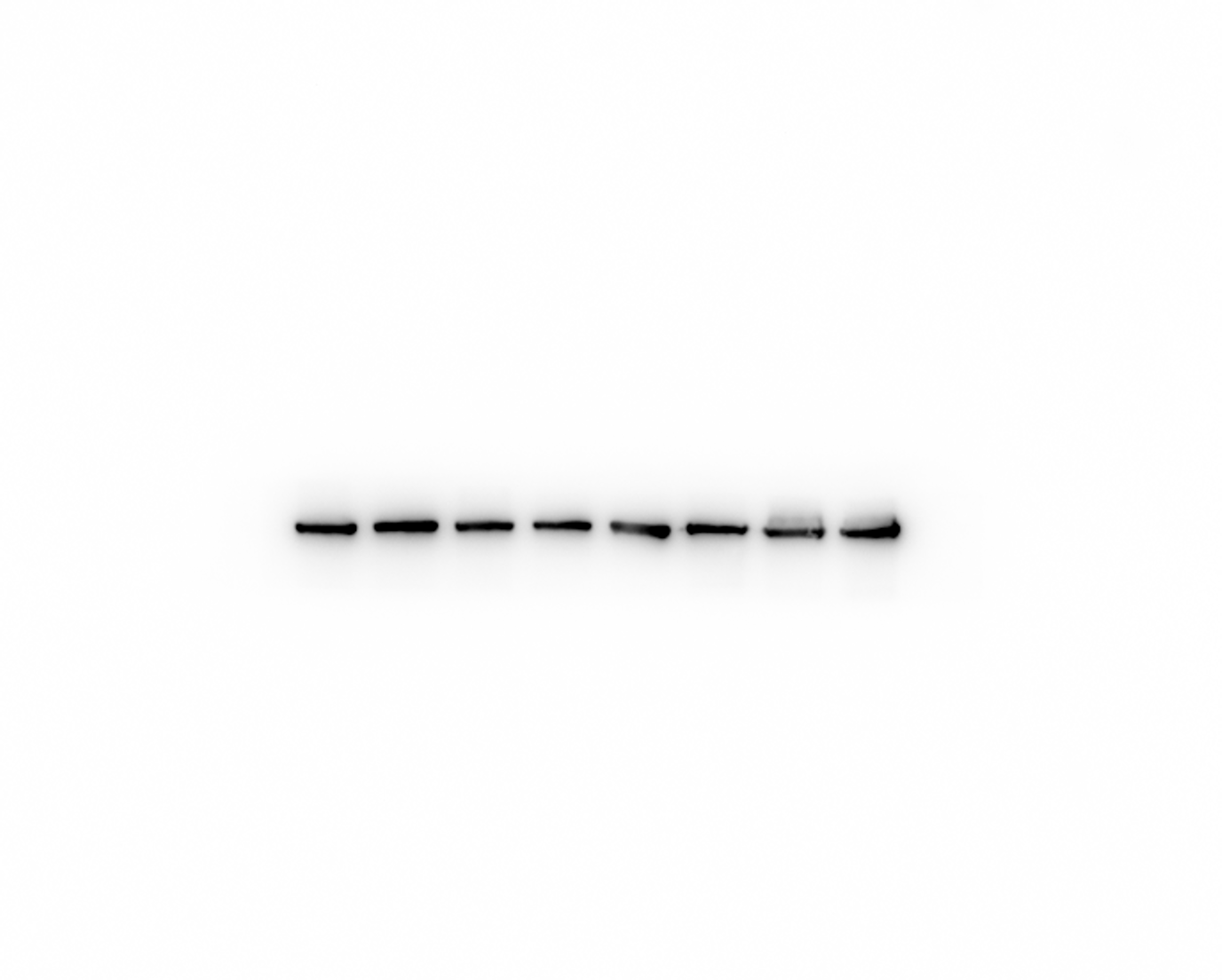


Tubulin 55kDa

MC38 CT26 DLD1 HCT15

Thimerosal

PBS

Thimerosal

PBS

Thimerosal

PBS

Thimerosal

PBS


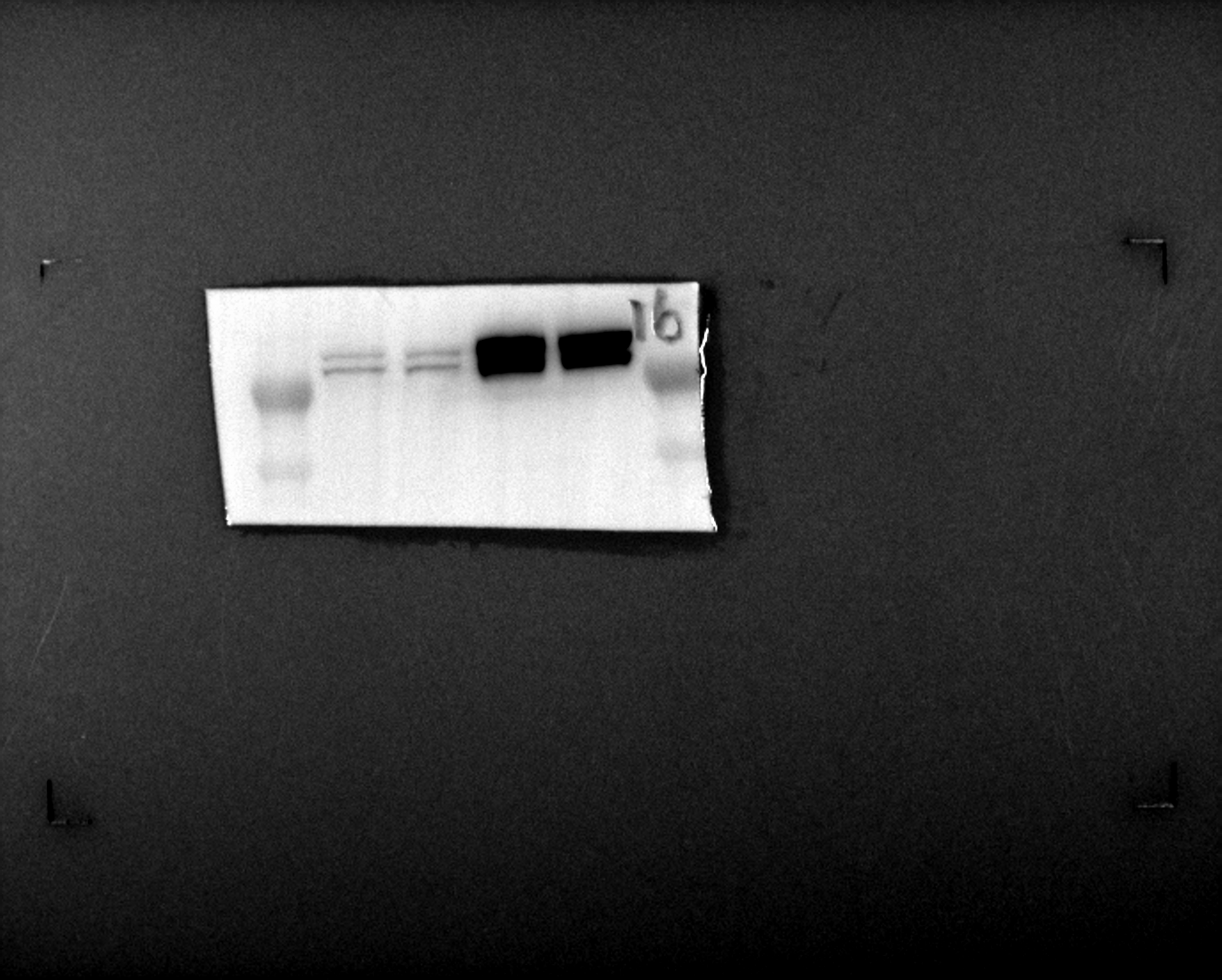

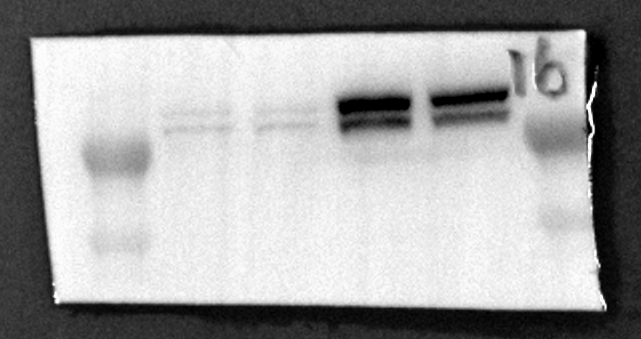

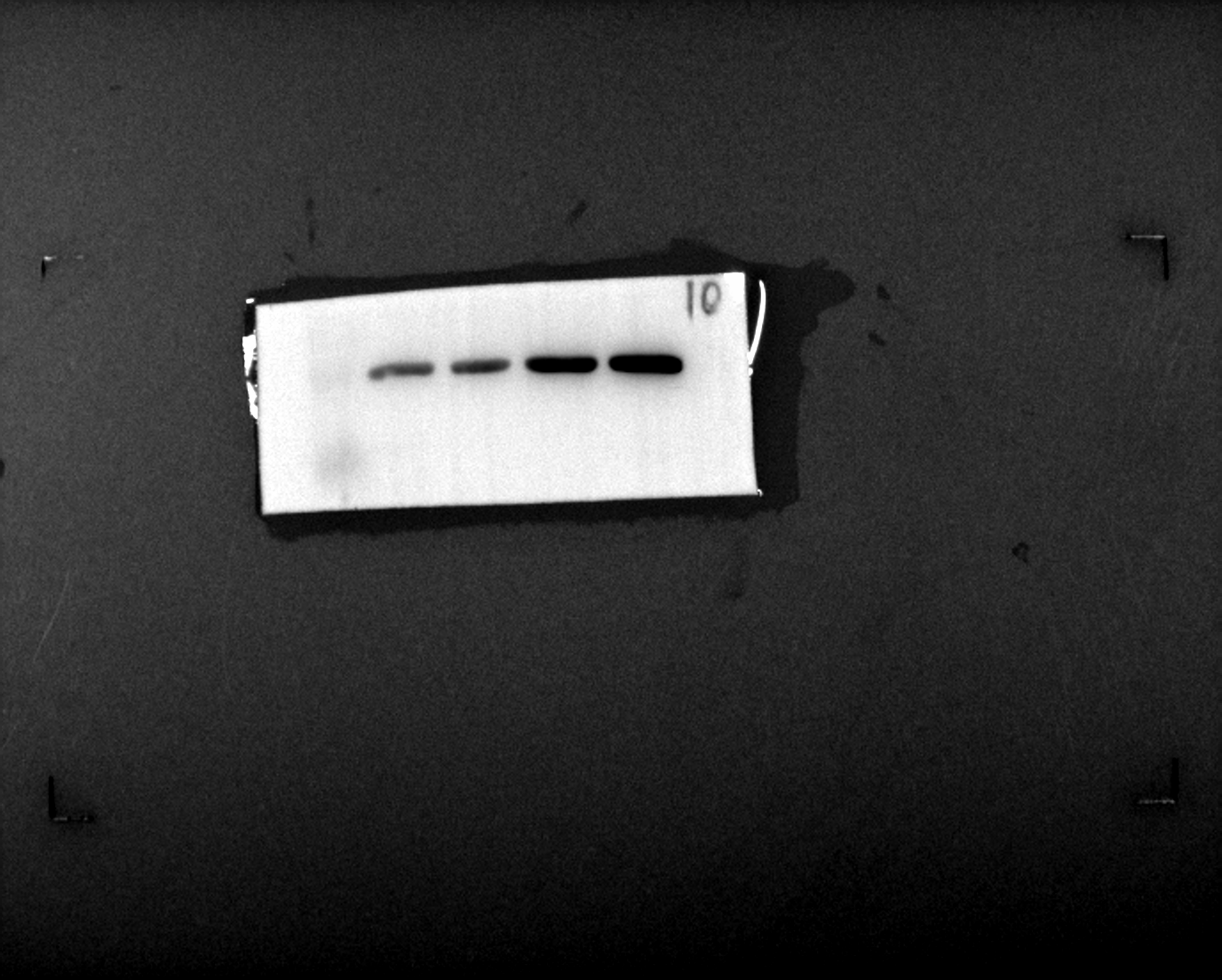

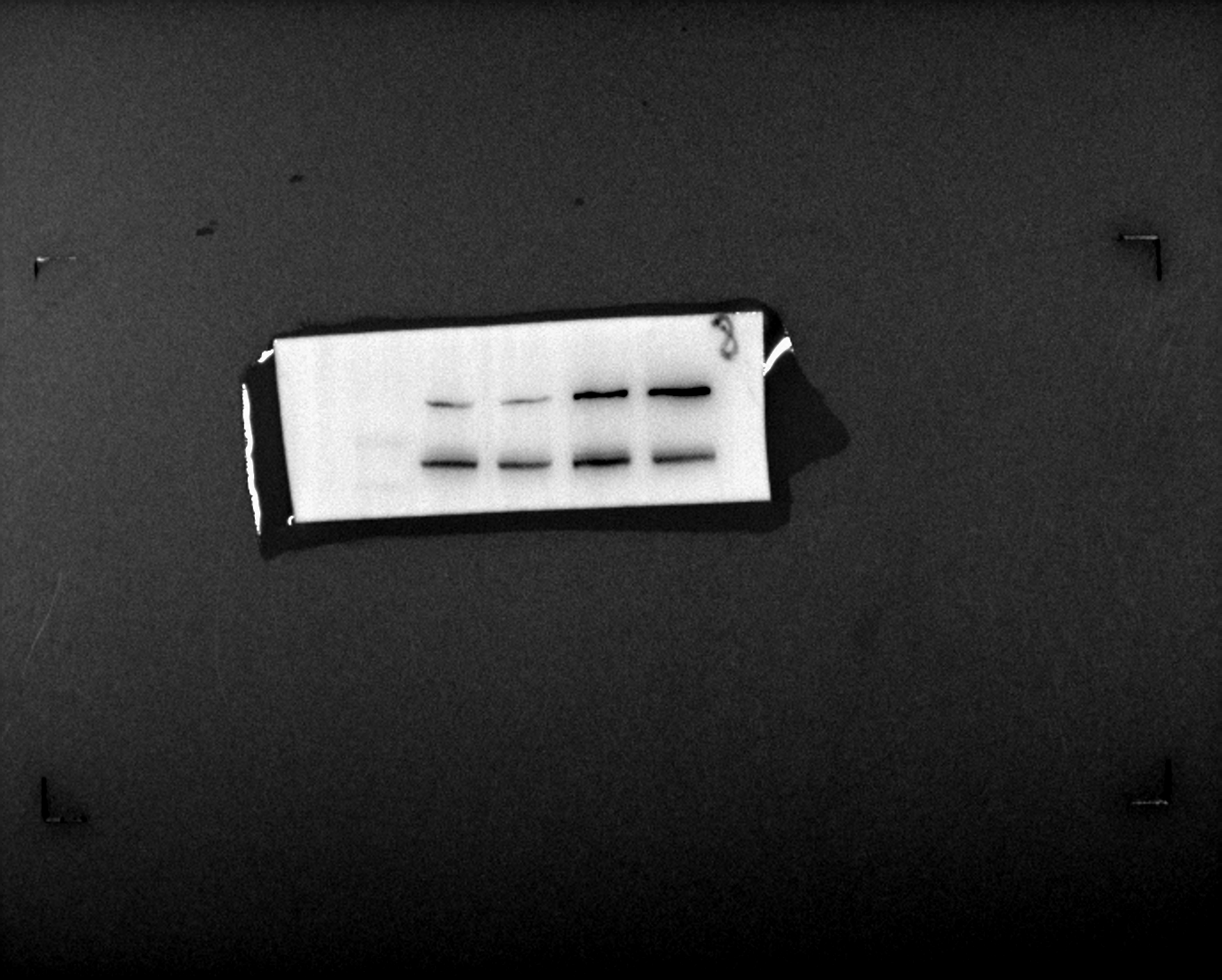

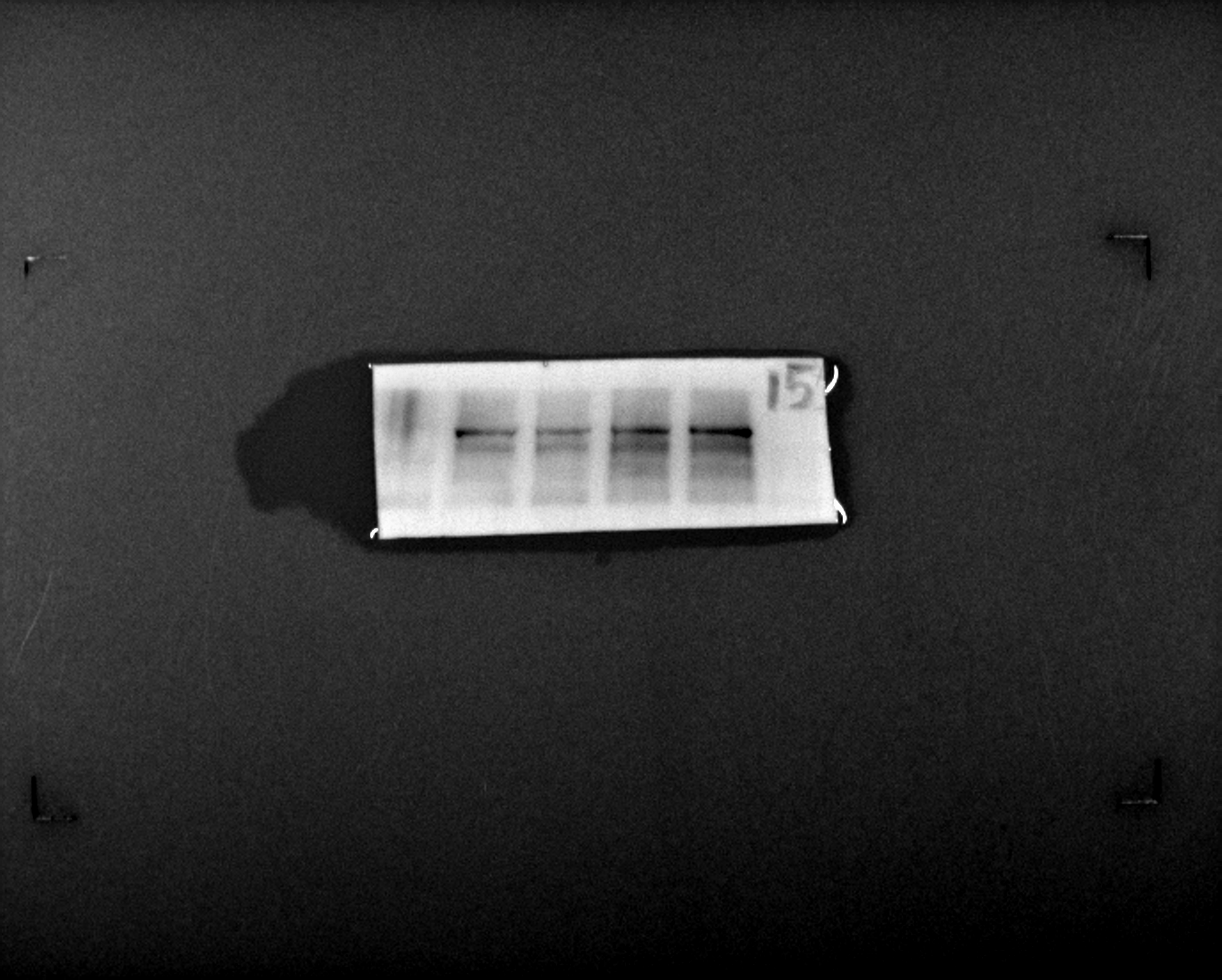

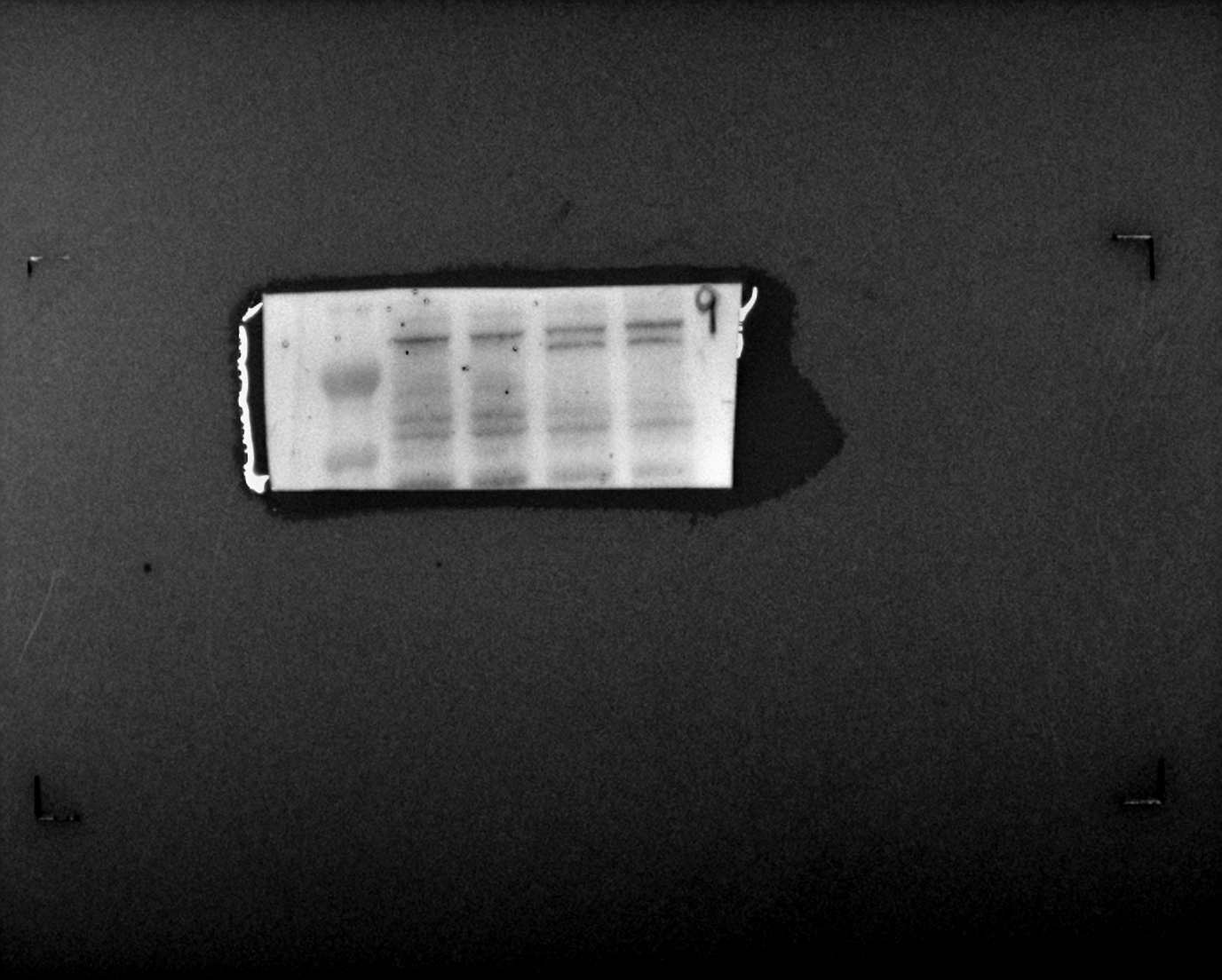


STAT3 88kDa


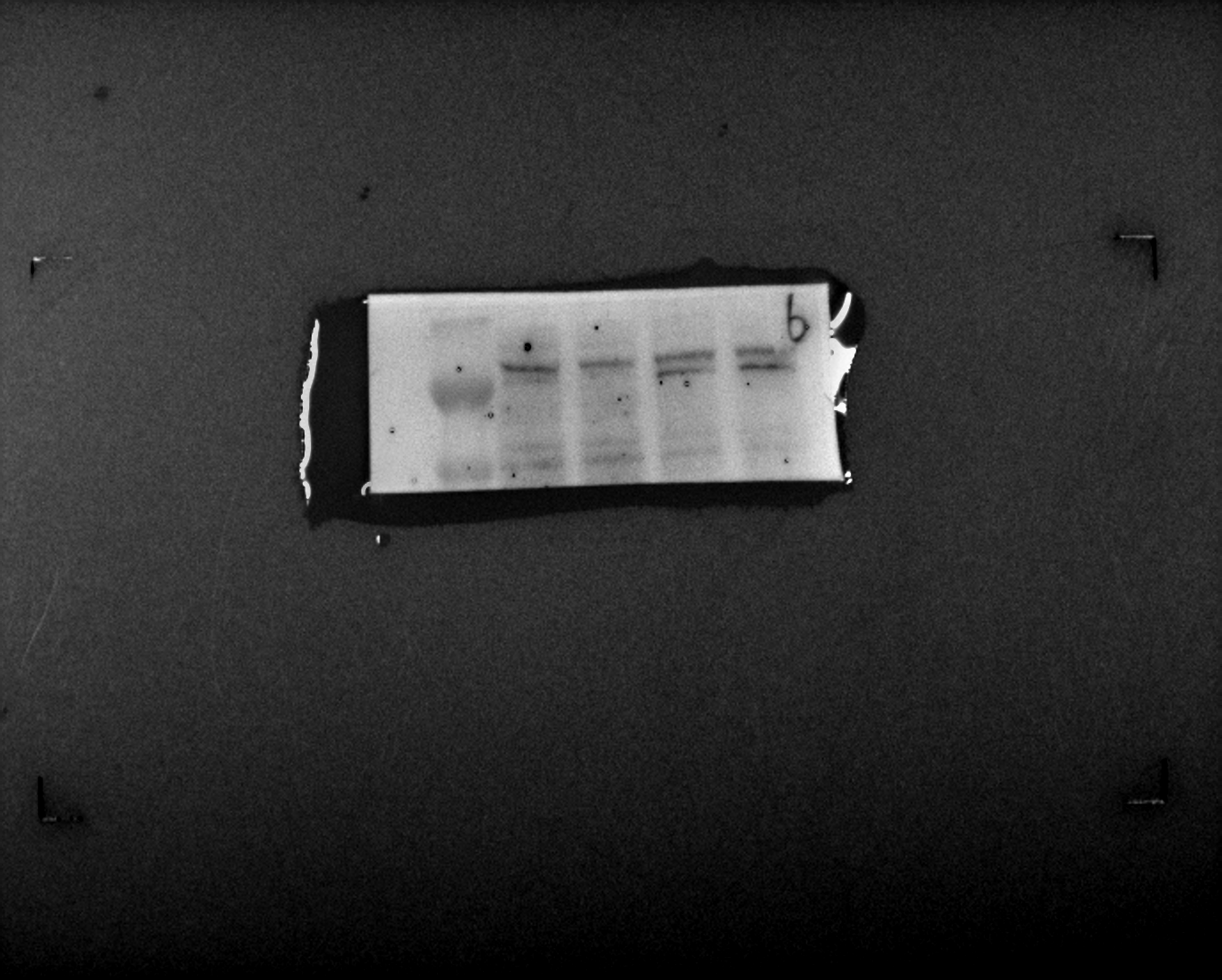


p-STAT3 88kDa


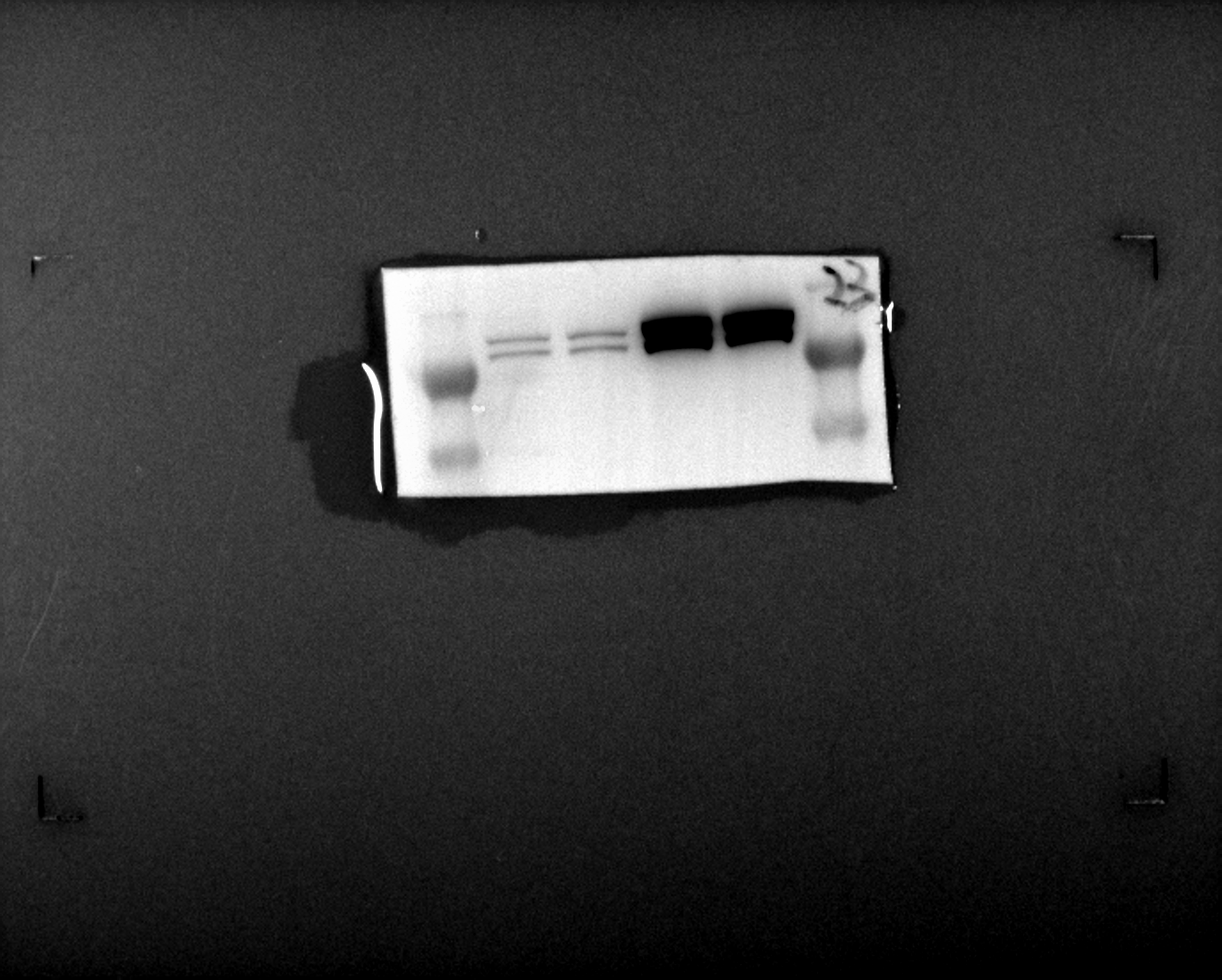

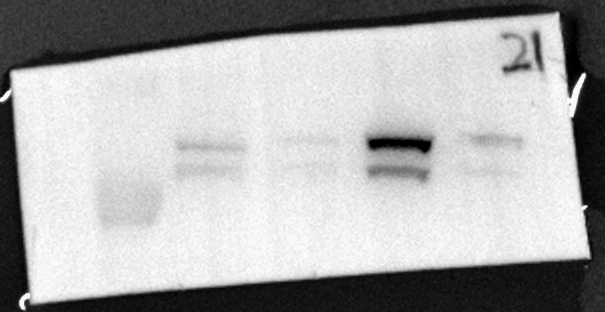


70

p-STAT3 88kDa


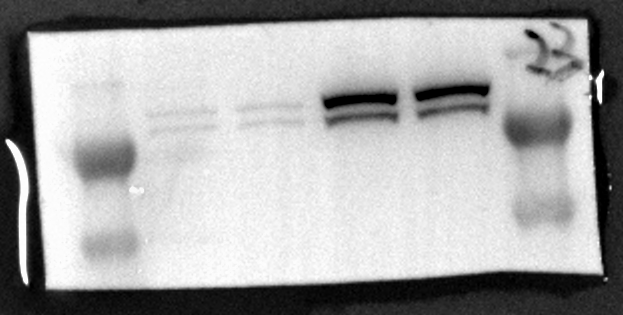


GAPDH 37kDa


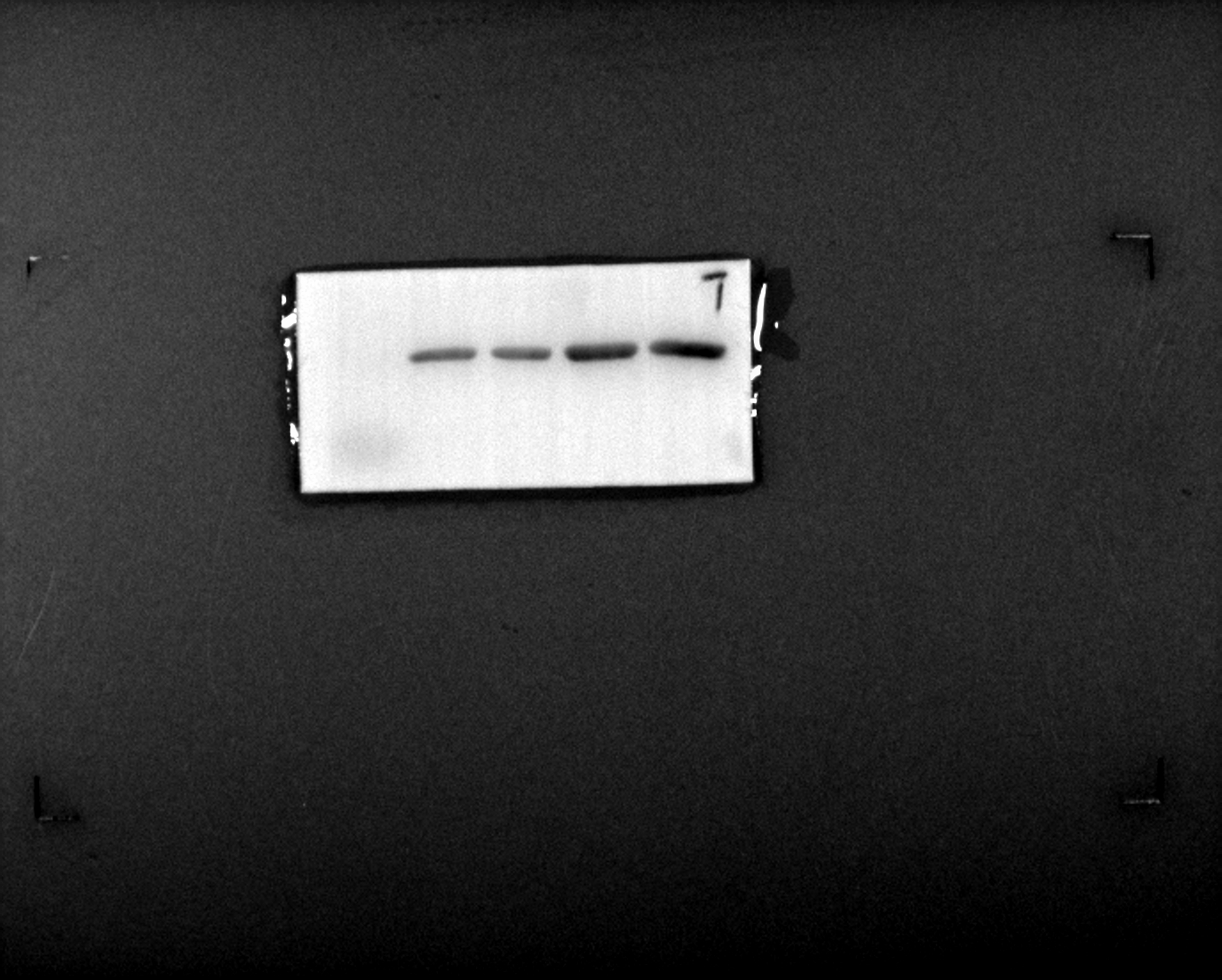

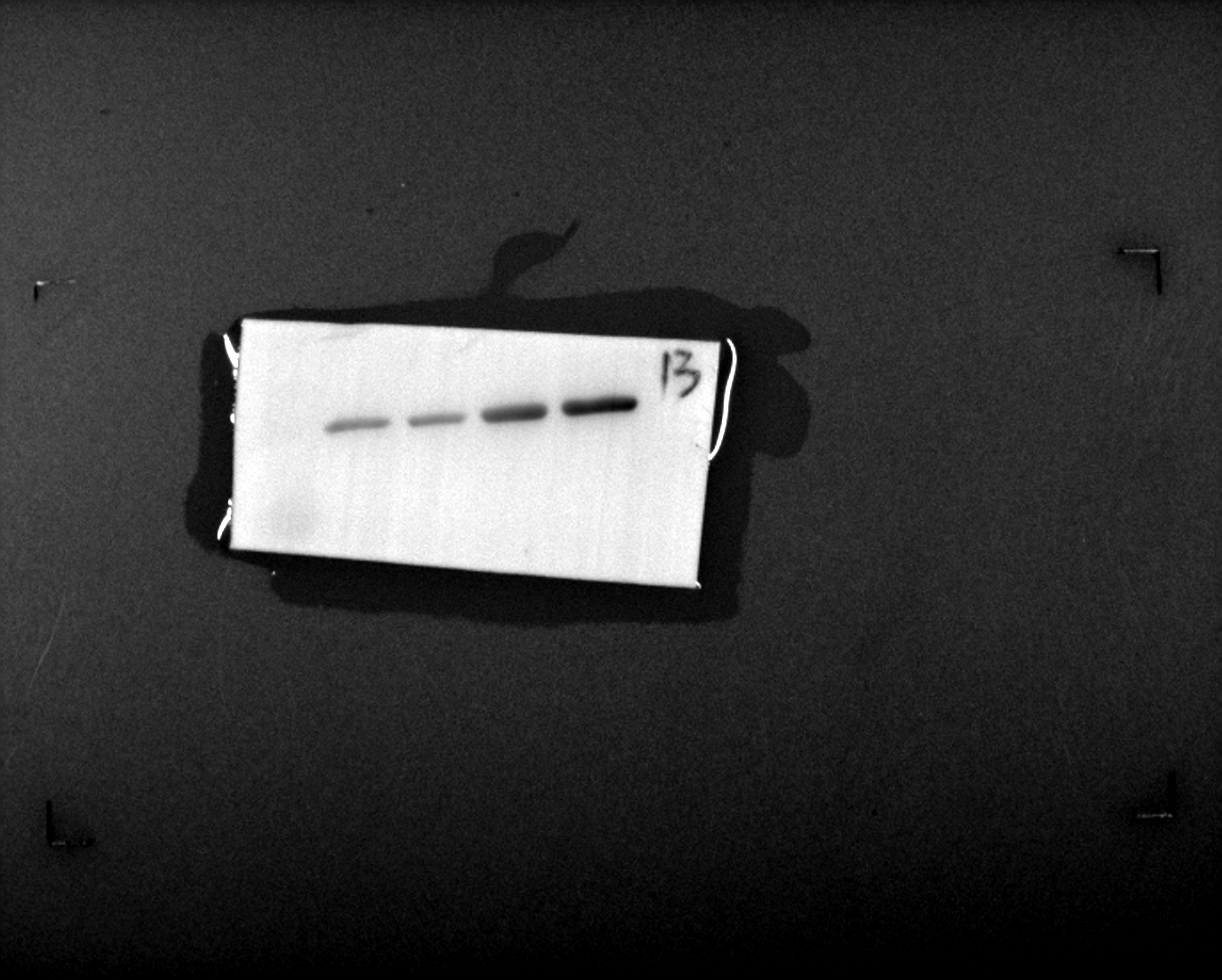


35

25

**Figure 5D**

p-JAK1 130kDa


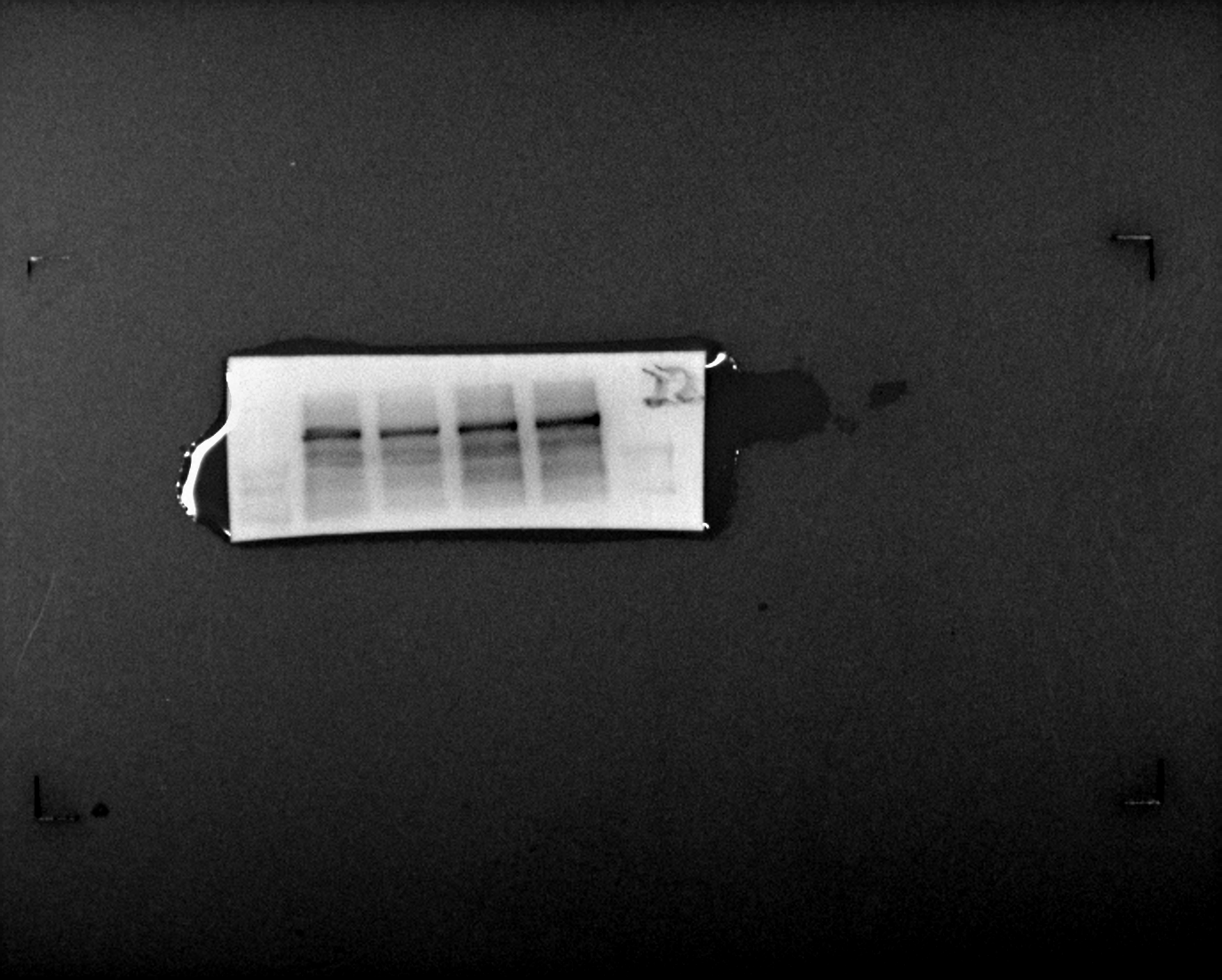

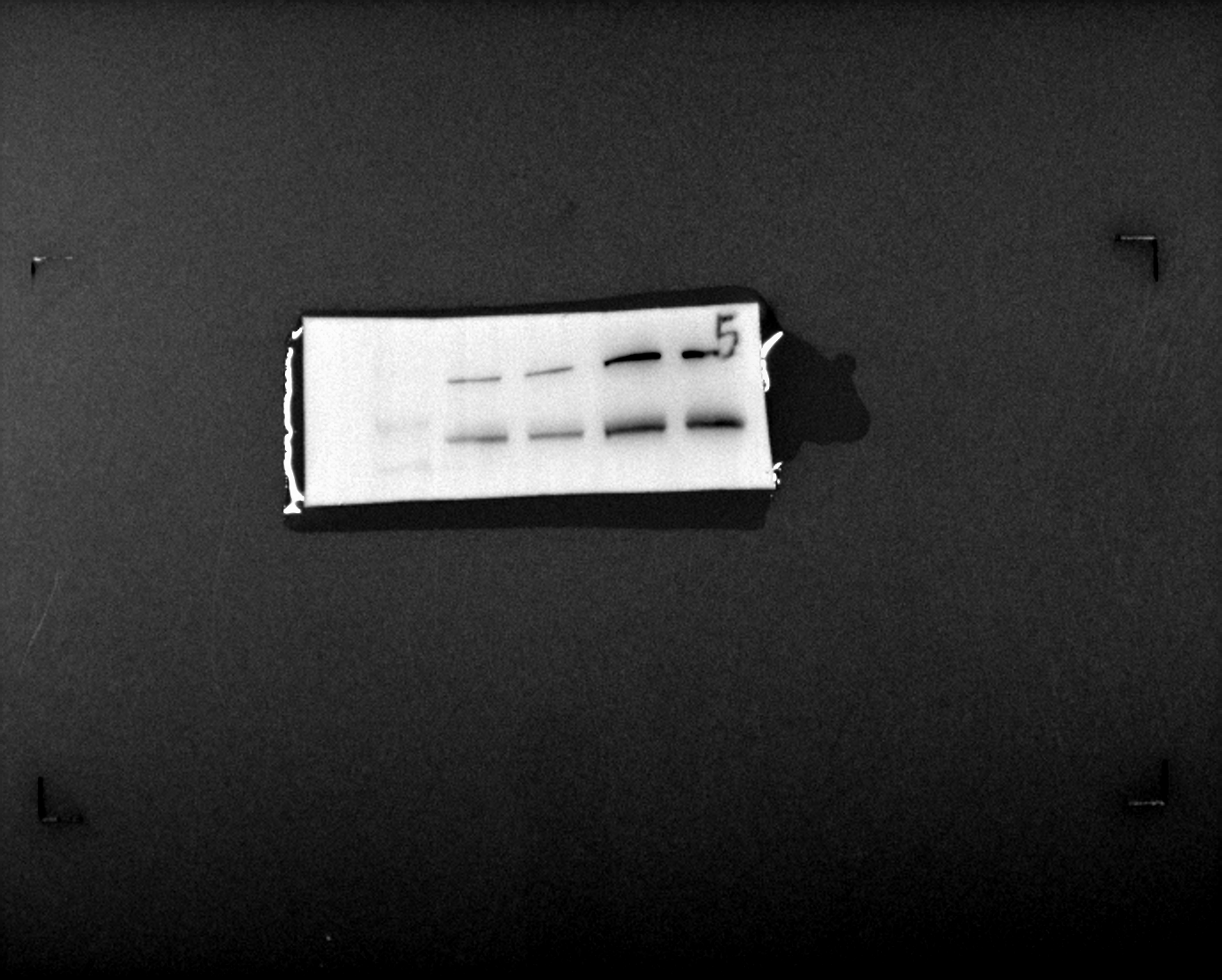


JAK1 130kDa

PBS

CPD2

PBS

CPD2

PBS

CPD1

PBS

CPD1

PBS

Thimerosal

PBS

Thimerosal


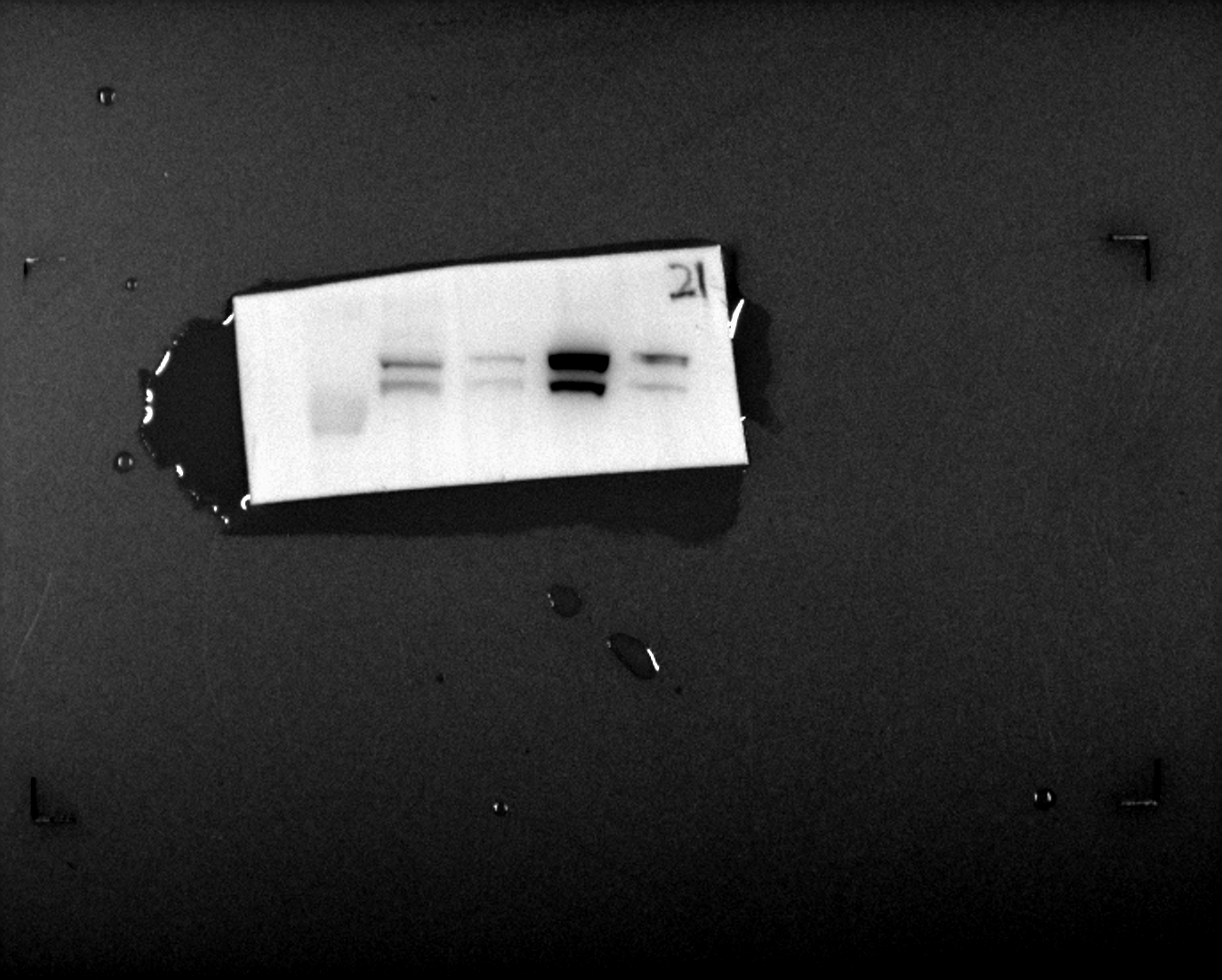


70


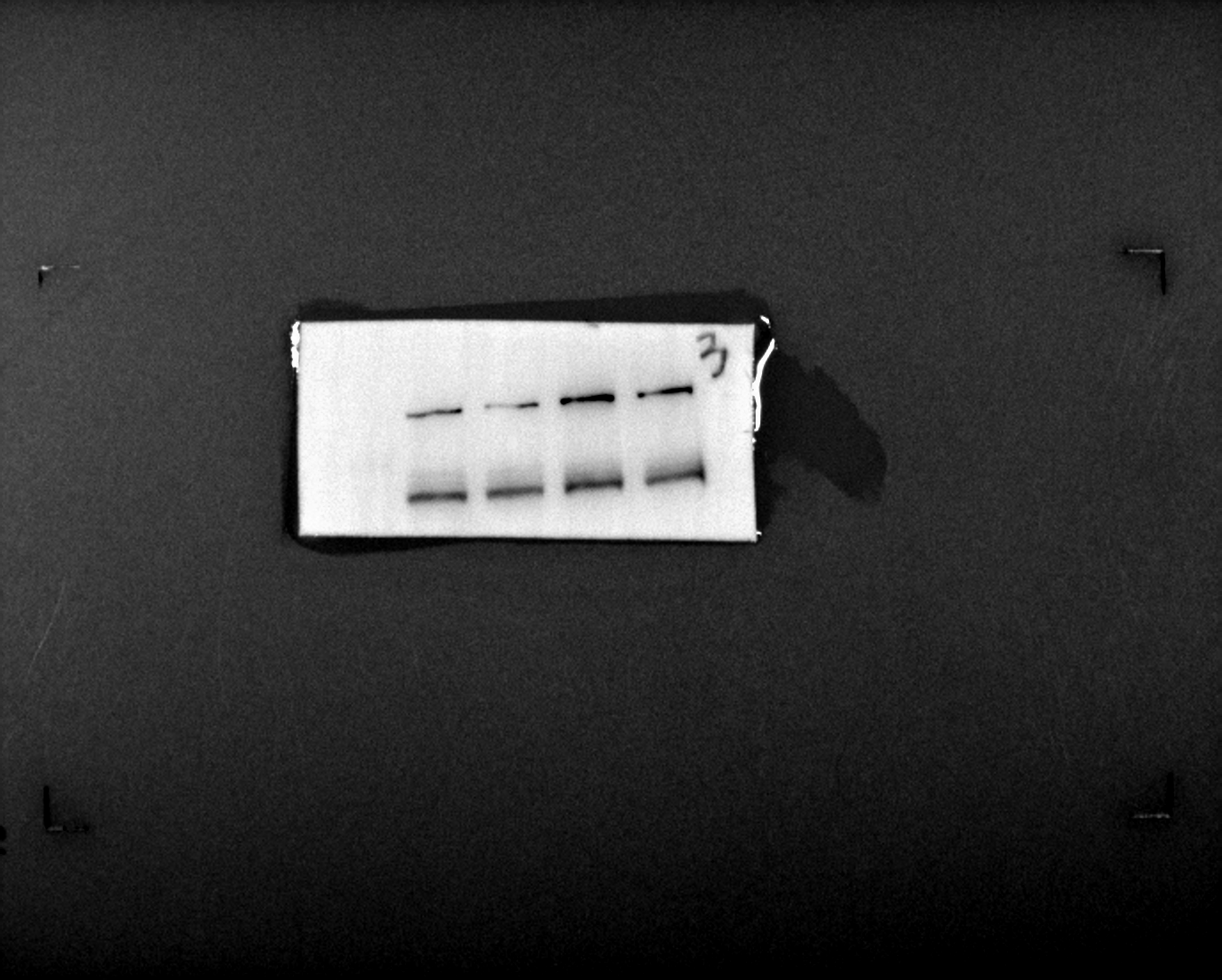


150

100


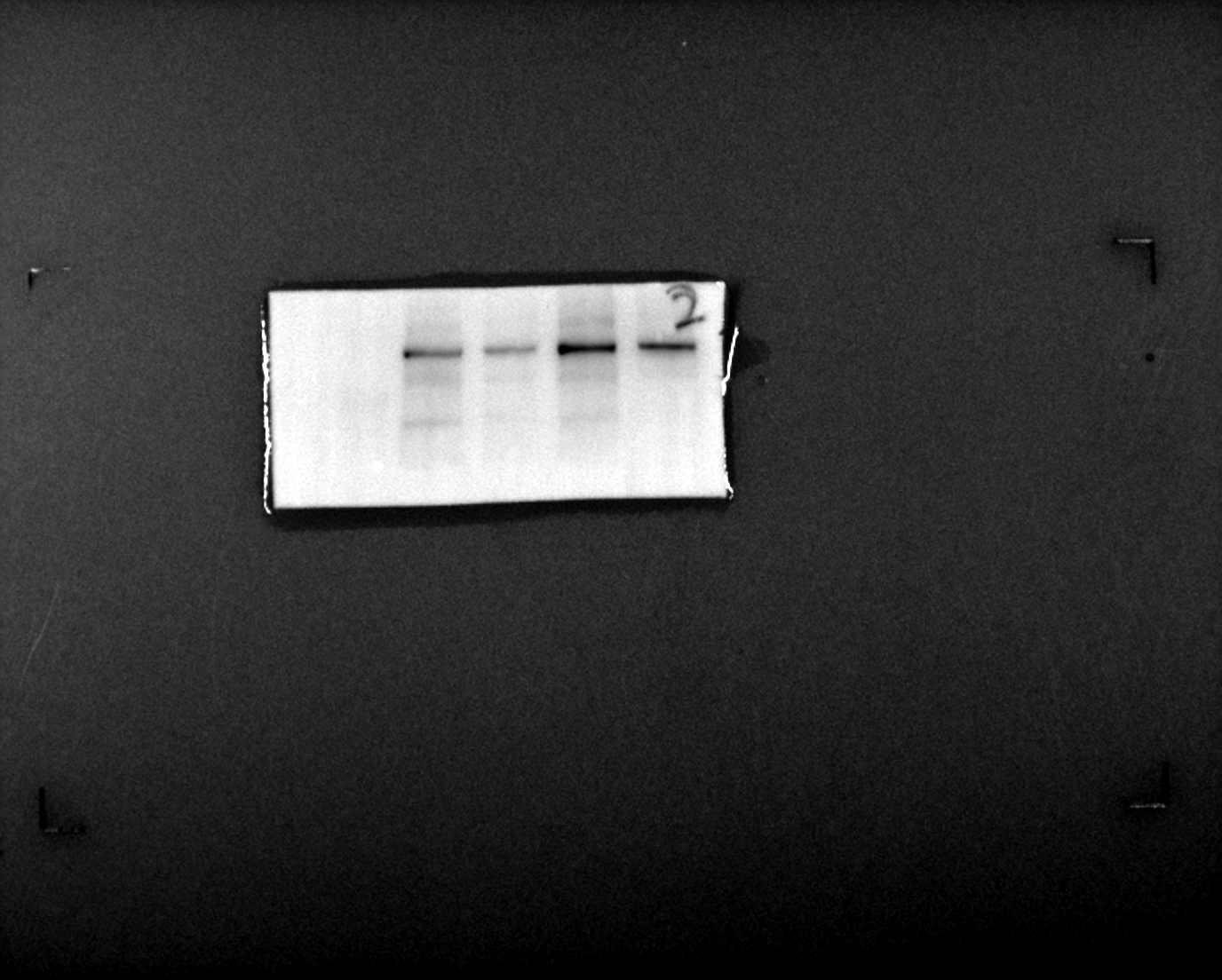


CT26

B16F10

CT26

B16F10

CT26

B16F10


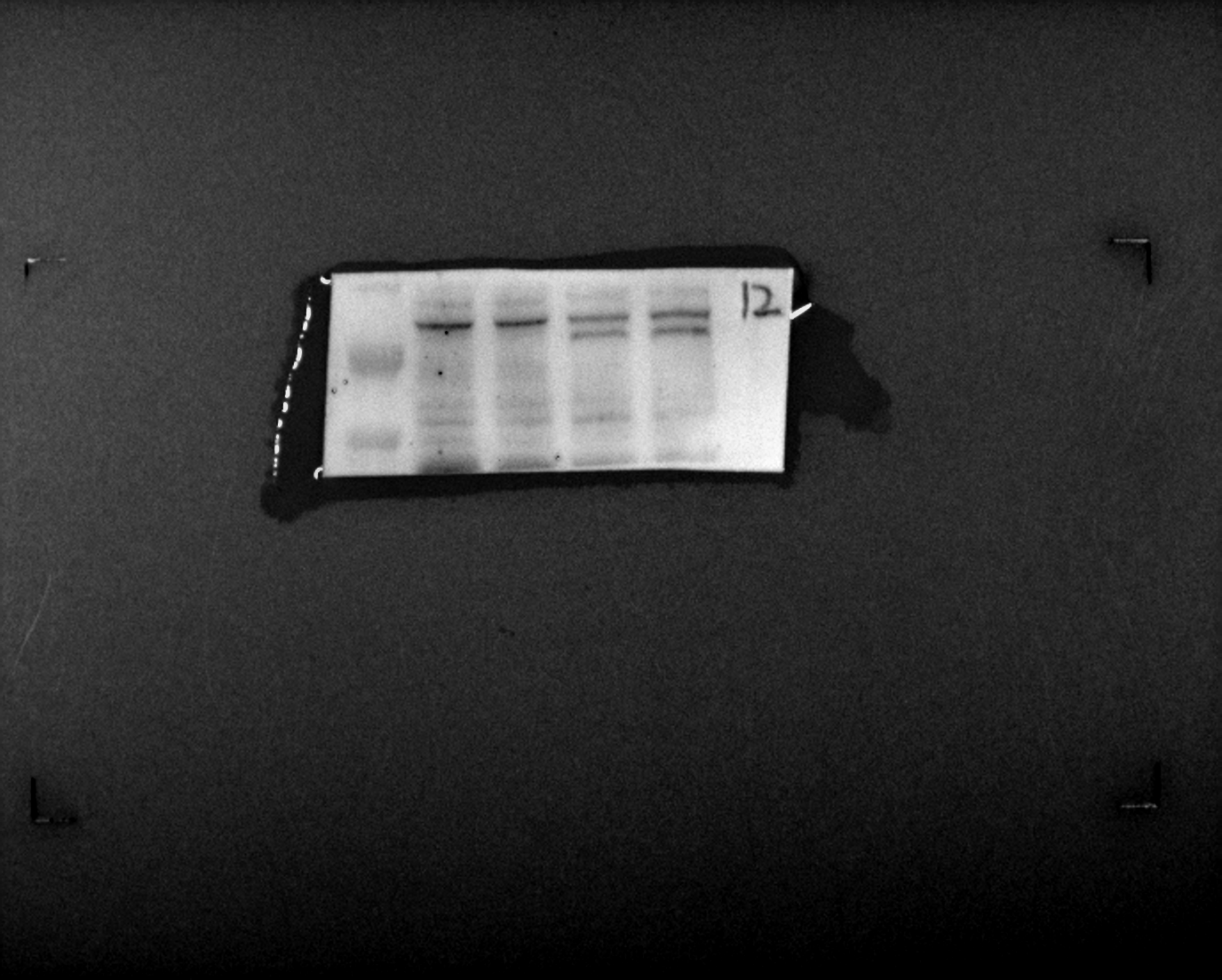


70

50
