# Supplementary material for: Thimerosal Inhibits Tumor Malignant Progression through Direct Action and Enhancing the Efficacy of PD-1-Based Immunotherapy
Source: Oncol Res. 2026 Jan 19;34(2):20. doi: 10.32604/or.2025.071902 (PMC12848756; doi:10.32604/or.2025.071902)
Supplement: Supplementary file 3 [file OncolRes-34-71902-s003.pdf]

Mycoplasma test

Sample information

Number of samples: 14

Sample properties: cell culture medium

Testing items: Mycoplasma detection

Detection method: PCR method was used to amplify Mycoplasma specific sequence and agarose electrophoresis analysis results.

Experimental process

(I) Sample preparation

Sample category: Culture supernatant

Take 1 ml of the culture supernatant sample in a clean sterilized 1.5 ml centrifuge tube, centrifuge at 13,000 rpm to collect the precipitate, add an appropriate amount of lysate at 95 °C for 5 min, and centrifuge to retain the supernatant for testing.

(II) Experimental system

PCR SYSTEM (ul)	Negative control	Positive control	Sample1	Sample2	Sample3	Sample4	Sample5	Sample6
PCR-Mix	10	10	10	10	10	10	10	10
PrimeF	1	1	1	1	1	1	1	1
PrimeR	1	1	1	1	1	1	1	1
RNA Free Water	6	6	6	6	6	6	6	6

Negative control	2	-	-	-	-	-	-	-
Positive Control	-	2	-	-	-	-	-	-
SampleDNA	-	-	2	2	2	2	2	2
Total volume	20	20	20	20	20	20	20	20

PCR SYSTEM (ul)	Sample7	Sample8	Sample9	Sample10	Sample11	Sample12	Sample13	Sample14
PCR-Mix	10	10	10	10	10	10	10	10
PrimeF	1	1	1	1	1	1	1	1
PrimeF	1	1	1	1	1	1	1	1
RNA Free Water	6	6	6	6	6	6	6	6
Negative control	-	-	-	-	-	-	-	-
Positive Control	-	-	-	-	-	-	-	-
SampleDNA	2	2	2	2	2	2	2	2
Total volume	20	20	20	20	20	20	20	20

Primer sequence

mycoplasma F: ACACCATGGGAGCTGGTAAT

mycoplasma R: CTTCWTCGACTTYCAGACCCAAGGCA

PCR reaction detection is performed according to the above system configuration.

(III) Circular Program

94℃ 2min	1cycle
94℃ 30sec	34cycle
55℃ 2min	
72℃ 1min	

4°C 5min

The PCR reaction procedure was set according to the above cycle, the product was 2% agarose gel electrophoresis, the PCR product and maker were all sampled for 5ul, and the gel imager was observed and photographed.

Test results

(I) Experimental results

The electrophoresis results are shown below:

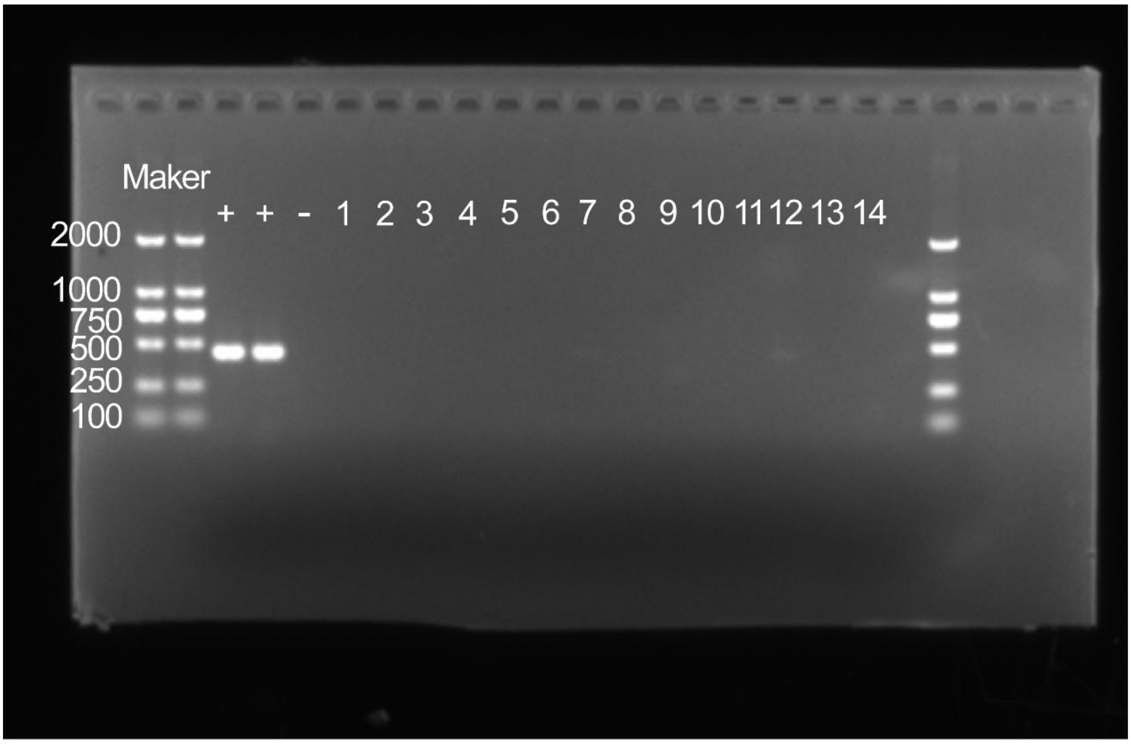

Negative PCR products have no bands, and positive PCR products have 450bp bands, indicating that the experiment is successful. The test product has no bands, which indicates no Mycoplasma contamination, and a 450bp band indicates Mycoplasma contamination.

(II) Conclusion

sample	Cell Lines	Mycoplasma contamination
1	CT26	Negative
2	MC38	Negative
3	B16F10	Negative
4	NCM460	Negative
5	HCT116	Negative
6	SW480	Negative
7	SW620	Negative
8	DLD1	Negative
9	HCT8	Negative
10	HCT15	Negative
11	HT29	Negative
12	RKO	Negative
13	LOVO	Negative
14	LS-174T	Negative

Report date: 2025.08.01
